# Lung cancer risk prediction using interpretable ensemble models on lifestyle and clinical data

**DOI:** 10.1371/journal.pone.0357291

**Published:** 2026-09-03

**Authors:** Shahid Mohammad Ganie, Pijush Kanti Dutta Pramanik, Zhongming Zhao

**Affiliations:** 1 Department of Health Information Management and Technology, College of Applied Medical Sciences, King Faisal University, Al-Ahsa, Saudi Arabia; 2 School of Computer Applications and Technology, Galgotias University, Greater Noida, Uttar Pradesh, India; 3 Center for Precision Health, McWilliams School of Biomedical Informatics, The University of Texas Health Science Center at Houston, Houston, Texas, United States of America; The University of Texas, MD Anderson Cancer Center, UNITED STATES OF AMERICA

## Abstract

Lung cancer remains one of the leading causes of cancer-related mortality worldwide, where early detection and reliable risk assessment are critical for improving outcomes. This study develops and evaluates a range of ensemble learning models for lung cancer prediction using lifestyle and clinical indicators, with an emphasis on both predictive performance and interpretability. Five base learners—logistic regression, k-nearest neighbors, naïve Bayes, support vector machine, and linear discriminant analysis—were used to construct multiple boosting and bagging models. Building on these, voting and stacking ensembles were designed by selectively combining high-performing models. All approaches were evaluated on the original dataset as well as on balanced and upsampled variants derived through synthetic augmentation. Model performance was assessed using accuracy, precision, recall, F1-score, Matthews correlation coefficient (MCC), and AUC-ROC. The results show that ensemble approaches consistently outperform individual models, with voting and stacking demonstrating superior performance over boosting and bagging methods. The stacking model achieved the strongest overall performance across all evaluated models. On the original dataset, which provides a more realistic representation of practical deployment conditions, it attained an accuracy of 93.53%. Performance further improved on the balanced and upsampled datasets on the upsampled dataset. To enhance transparency, SHAP and LIME were employed to provide global and local interpretability, respectively, enabling identification of key clinical factors and patient-specific risk drivers. The analysis highlights both alignment with known clinical patterns and dataset-driven variations, supporting informed interpretation of model outputs. The results suggest that stacking-based ensembles can improve risk prediction from lifestyle and clinical indicators while maintaining model transparency through SHAP and LIME explanations. These findings highlight the potential of interpretable ensemble learning as a decision-support tool for lung cancer risk assessment.

## 1. Introduction

The modern lifestyle has induced many critical and life-threatening diseases in humankind around the world. Among these, cancer continues to be the leading cause of mortality, responsible for around 10 million deaths in 2020, which equates to one in every six fatalities globally [1]. Lung cancer is one of the most common types of cancer [2]. According to the World Health Organization (WHO), 1.8 million people died worldwide in 2020 due to lung cancer. Hence, it has become a significant challenge for healthcare professionals and patients. It is noted that if the disease is detected at an early stage, a person's chances of survival can be increased.

For the early detection of lung cancer, it is crucial to accurately distinguish between benign (non-cancerous) and malignant (cancerous) cells [3]. Various lung diagnosis methods exist that utilise imaging techniques, including CT (computed tomography), isotope, X-ray, and MRI (magnetic resonance imaging), as well as molecular screening, among others [4]. These image-analysis-based diagnostic methods still face the challenge of distinguishing benign from malignant cancers, as they share similarities. Importantly, these techniques employ a reactive approach, i.e., they can detect a cancerous cell once it has become such. Instead, a proactive approach would be a better tactic, in which the probability of a person getting cancer, based on her lifestyle data, is predicted. Machine learning techniques provide an opportunity to identify patterns in lifestyle and clinical data that may support earlier risk assessment.

Machine learning has shown significant potential for medical professionals and researchers to discover hidden prospects in medical, clinical, and demographic data and better serve human beings [5,6]. In the healthcare sector, machine learning is useful for analysing diseases with acceptable accuracy, allowing doctors to make better medical decisions, which in turn leads to improved diagnosis and prognosis. Machine learning techniques are successfully applied to predict and diagnose fatal diseases, such as cancer, heart disease, and brain tumours. However, traditional machine learning approaches may struggle to capture increasingly complex and non-linear relationships present in large heterogeneous healthcare datasets [7].

Traditional machine learning algorithms, such as logistic regression (LR), naive Bayes (NB), K-nearest neighbour (KNN), support vector machine (SVM), linear discriminant analysis (LDA), etc., have some limitations in cancer prediction due to the disease's complexity, dynamicity, and the heterogeneity of data [8]. Some of the significant limitations are summarized below.

- Limited feature selection: Feature selection poses a significant challenge in cancer prediction using conventional machine learning algorithms. Cancer is a complex ailment impacted by multiple variables, including age, gender, lifestyle choices, genetic mutations, and environmental influences. Identifying the most relevant features that can effectively forecast cancer occurrence remains challenging.
- Inefficiency in handling imbalanced data: Cancer data shows an imbalance, where individuals diagnosed with cancer represent a smaller population than those without cancer. Conventional machine learning algorithms may show bias toward the dominant class, leading to suboptimal performance in cancer prediction.
- Inefficiency in handling non-linearity: Cancer data often exhibits non-linear characteristics, indicating that feature-outcome associations are not linear. Conventional machine learning algorithms, such as logistic regression and linear discriminant analysis, assume linear associations between features and outcomes. However, when applied to cancer prediction tasks, this assumption may produce suboptimal performance.
- Overfitting: Conventional machine learning algorithms are prone to overfitting, where the model's excessive complexity captures noise rather than underlying patterns. Overfitting can result in suboptimal generalization performance and diminished predictive accuracy in cancer prediction.
- Interpretability: Machine learning algorithms are often characterized as black-box models, offering limited transparency into mechanisms governing cancer prediction. The importance of interpretability in clinical decision-making lies in helping clinicians understand factors that contribute to cancer prediction.

Employing more sophisticated machine learning methodologies, such as deep learning and ensemble techniques, may help address these constraints. In recent years, ensemble learning paradigms, such as boosting, bagging, voting, and stacking, have attracted increasing attention from the research community. These approaches have shown promising results, demonstrating improved accuracy in the prediction of diseases [9].

Ensemble learning represents a machine learning paradigm that combines the predictions of multiple individual models to improve overall accuracy and reduce the risk of overfitting [10]. Several benefits are associated with the use of ensemble learning techniques in cancer prediction. Some of them are mentioned below.

- Increased accuracy: The integration of multiple models through ensemble learning can enhance cancer prediction accuracy by leveraging individual strengths. Each model exhibits distinct strengths and weaknesses, and through their combination, the ensemble model can yield higher accuracy compared to any single model.
- Robustness: Ensemble learning techniques can enhance model robustness by mitigating the effects of outliers and noisy data. Outliers can significantly impact the efficacy of an individual model. However, in ensemble models, the influence of outliers diminishes due to the higher detection likelihood of other models within the ensemble.
- Better generalization: Ensemble learning techniques mitigate overfitting by combining multiple models with distinct biases and assumptions. The ensemble model exhibits a decreased propensity to conform to data noise, thereby capturing fundamental patterns and leading to enhanced generalization performance.
- Flexibility: Ensemble learning is a versatile approach that can be applied to Decision Trees (DTs), neural networks, and support vector machines. It can be tailored to address diverse cancer prediction tasks, including diagnosis, prognosis, and risk stratification.

It is imperative to recognize that the straightforward application of ensemble learning does not inherently yield the anticipated advantages. The selection of ensemble techniques and foundational models should be informed by the specific characteristics of the problem, dataset properties, and available computational resources [10]. When applied appropriately, ensemble learning has the potential to markedly enhance the performance of machine learning models [11].

Although ensemble models improve predictive accuracy, they often lack interpretability and obscure the prediction process. Furthermore, while explainable artificial intelligence (XAI) is increasingly prevalent in healthcare, its application in ensemble models for lung cancer prediction remains limited [12–14]. Most research has focused on elucidating individual models with XAI rather than complex ensemble systems, thereby constraining the comprehensibility of these models in real-world clinical contexts. Despite advancements in predictive models within healthcare, there remains a paucity of models that integrate the accuracy of ensemble learning with the interpretability of XAI for lung cancer. Given the multitude of interconnected factors influencing these conditions, understanding model predictions is essential for clinicians to tailor treatments. Traditional ensemble methods, although effective, often function as “black boxes,” concealing the rationale behind predictions. This opacity diminishes their utility in clinical settings, where providing clear, patient-specific insights for ethical, informed decision-making is crucial.

To address this challenge, this study proposes an XAI-enhanced stacking model that not only delivers high predictive performance for lung cancer risk assessment but also facilitates transparent interpretation of model outputs. In the domain of clinical decision-making, where transparency and accountability are critical, interpretable AI methods such as SHAP (SHapley Additive exPlanations) and LIME (Local Interpretable Model-agnostic Explanations) play a pivotal role. SHAP values provide both global and local insights into feature significance, facilitating a comprehensive understanding of the factors influencing lung cancer risk predictions. LIME, on the other hand, provides localised explanations, enabling the interpretation of specific predictions on an instance-by-instance basis. By integrating these explainability techniques, we ensure statistical rigor in performance validation, thereby rendering our approach more reliable and explainable for clinical adoption.

The major contributions of this paper are as follows:

- We develop and evaluate multiple ensemble learning strategies (boosting, bagging, voting, and stacking) for lung cancer prediction using lifestyle and clinical data.
- We perform a systematic comparison of base learners (LR, KNN, NB, SVM, and LDA) and demonstrate how ensemble methods leverage their complementary strengths.
- We analyze model performance across original, balanced, and upsampled datasets, highlighting the impact of data preprocessing on predictive outcomes.
- We show that the stacking ensemble achieves the most consistent overall performance across multiple evaluation metrics, while also identifying trade-offs with respect to individual metrics.
- We incorporate explainable AI techniques (SHAP and LIME) to provide both global and patient-specific interpretability, linking model outputs to clinically meaningful factors.
- We conduct statistical significance analysis using non-parametric tests to validate that the observed performance differences are robust and not due to random variation.
- We compare multiple ensemble strategies under original, balanced, and upsampled data settings to examine how data distribution influences predictive performance.

The rest of the paper is organized as follows. Section 2 presents the related work. Section 3 describes the proposed methodology and provides an overview of the considered machine learning and ensemble learning algorithms. Section 4 details the dataset. Section 5 presents and analyzes the experimental results. Sections 6 and 7 report the statistical significance analysis and interprets the proposed models using XAI, respectively. Section 8 discusses the findings and their clinical implications. Finally, Section 9 concludes the study, outlining its limitations and directions for future work.

## 2. Related work

Machine learning and ensemble-based methodologies have demonstrated substantial potential to produce consistent, reliable, and valid outcomes, thereby finding widespread application across various real-world domains. In the context of lung cancer prediction, significant research has employed these techniques to enhance diagnostic accuracy. For example, Abdullah et al. [15] conducted a comparative analysis in terms of accuracies SVM, KNN, and a convolutional neural network (CNN) in classifying lung cancer in its early stages. In their study, SVM achieved the highest accuracy (95.56%), while KNN achieved the lowest (88.40%). Patra [16] utilized machine learning classifiers, including NB, KNN, and Radial Basis Function (RBF), using the Weka tool to analyse the lung cancer data. The dataset consisted of 32 instances with 57 features, collected from the University of California, Irvine (UCI) Machine Learning Repository. Among all the classifiers, RBF achieved the highest accuracy of 81.25% using 10-fold cross-validation. The author suggested that feature selection methods and integration with other machine learning models could enhance the efficacy of the proposed work. Radhika et al. [17] employed machine learning techniques such as DT, SVM, NB, and LR on two publicly available datasets (Data World and UCI) for lung cancer detection. By utilizing the UCI dataset, the LR model attained the highest accuracy rate of 96.90%, while SVM outperformed well and achieved an accuracy of 99.20% by using the world data repository.

Numerous studies have employed a variety of datasets, algorithms, and methodologies to advance research in lung cancer prediction. An increasing number of researchers have explored the effectiveness of ensemble learning techniques in improving diagnostic and prognostic accuracy for lung cancer. For instance, Ahmad and Mayyaa [18] designed a tool for lung cancer prediction using bagging and randomized node optimization. The authors considered various factors associated with lung cancer, such as genetic risks, air pollution, smoking, dust allergy, etc. and symptoms like coughing blood, fatigue, dry cough, shortness of breath, chest pain, and others. In a related study, Safiyari and Javidan [19] employed five widely adopted ensemble techniques, including bagging, dagging, AdaBoost (ADB), multi-boosting, and random subspace using data from the Surveillance, Epidemiology, and End Results (SEER) program. Wang et al. [20] introduced an ensemble-based model combining Random Forest (RF) with a self-paced learning bootstrap approach to enhance lung cancer classification and prognosis. Their methodology employed a sampling strategy that progressively integrated data from high- to lower-quality samples. Mamun et al. [21] implemented various ensemble algorithms, including Extreme Gradient Boosting (XGB), Light Gradient Boosting Machine (LGBM), bagging, and ADB, evaluated through 10-fold cross-validation. Among these, XGB demonstrated superior performance, achieving an accuracy of 94.42%.

Ensemble learning methodologies, when paired with XAI, have attracted significant attention in lung cancer prediction. This interest stems from their dual ability to enhance predictive performance while providing interpretability—an essential component in clinical decision-making and early diagnosis. Numerous studies have aimed to balance predictive accuracy with transparency. For instance, RF has been reported to achieve 98.9% accuracy across various datasets, highlighting its potential in medical diagnostics [22]. Another innovative framework, DeepXplainer, combines CNNs with XGB and achieves 97.43% accuracy. This model employs SHAP to offer both local and global interpretability, thereby improving the transparency of the system's predictions [23]. An interpretable radiomics-based model was developed using a stacked interpretable sequencing cells (SISC) framework, which not only demonstrated state-of-the-art results but also provided visualization tools like response maps to assist radiologists in decision support [24]. Similarly, the ExMed platform, which incorporates multiple feature attribution techniques, has been applied to synthetic healthcare data to predict lung cancer patients’ life expectancy, further illustrating the applicability of XAI in conjunction with ensemble learning models [25]. In another contribution, Siddhartha et al. [26] used a bagging-based ensemble method to classify the survival status of lung cancer patients by identifying and interpreting the risk factors. They developed a machine learning pipeline (RF) by mitigating the problem of imbalanced data. The central contribution of this study lies in its use of XAI to interpret RF predictions, a critical aspect for fostering trust and reliability in healthcare applications. However, the study lacks a comparative analysis with other ensemble or deep learning models, which limits insights into the relative effectiveness of RF. Zhang et al. [27] aimed to develop a prediction model using machine learning to identify high-risk groups for early identification and prevention of lung cancer. The performance of the models, including LR, NB, RF, and XGB was evaluated using the AUC (area under the curve), Brier loss, log loss, precision, recall, and F1 scores. The SHAP interpreter was used to visualize the models, showing the influence of each predictive factor on lung cancer risk. Another study, utilizing the SEER 2020 database, compared several traditional classifiers—LR and DT—with ensemble techniques like XGB, LGBM, and ADB, as well as deep learning models such as DNNs. LGBM emerged as the leading performer, with an accuracy of 87.45% and an AUC of 0.91 [28]. The study highlighted the need to integrate XAI tools to enhance the interpretability of deep learning frameworks, particularly for their potential use in clinical trials.

In summary, integrating ensemble methods—especially advanced techniques like stacking—with interpretability frameworks such as SHAP holds significant promise for enhancing both the performance and transparency of lung cancer prediction models. These combined approaches help build clinician trust in AI systems, addressing a long-standing barrier to AI deployment in clinical settings [29,30]. However, the current body of work primarily focuses on a limited set of ensemble techniques, with minimal comparative evaluation across a broader range of models. Most studies also confine themselves to conventional performance indicators like accuracy and F1-score, overlooking more nuanced metrics that could better assess clinical applicability. Furthermore, while XAI has been employed in many studies, its integration with complex ensemble frameworks remains underexplored. There is limited understanding of how model interpretability can be systematically integrated into clinical workflows or how different features interact synergistically to affect model outputs. This gap underscores the need for future research to investigate more complex feature interactions, broader datasets, and rigorous performance evaluation frameworks. Additionally, comparative studies that evaluate ensemble methods alongside traditional models could provide valuable insights into their respective advantages, limitations, and real-world applicability in healthcare settings.

## 3. Research methodology

This section provides a comprehensive overview of the research methodology employed and the ensemble learning techniques utilised in the experiment.

### 3.1 Research workflow

The following phases present the experiment:

- Phase I: The experiment begins by collecting lung cancer data and generating three datasets from the collected data.
- Phase II: We began with five prediction models, each using a different base learner: LR, NB, KNN, SVM, and LDA. Thereafter, we built five boosting ensemble models: CatBoost (CB), XGB, Gradient Boosting (GB), ADB, and LGBM, and five bagging ensemble models: DT, Bagged Decision Tree (BDT), RF, Extra Trees (ET), and Bagging Meta-Estimator (BME). Each model was applied to all three datasets. Hyperparameter tuning and stratified *k*-fold cross-validation were performed to optimize the performance of each model.
- Phase III: Voting and stacking models were built in the next phase. Initially, as constituent learners, we chose the top five models that performed best on each dataset. These models were then used to build ensemble structures, with optimization carried out individually on each dataset using hyperparameter tuning and stratified k-fold cross-validation.
- Phase IV: To achieve even better prediction performance, we judiciously selected the constituent learners and built voting and stacking models using them. Here again, each ensemble model was applied to the three datasets and optimized accordingly. The models’ performance was extensively evaluated using several standard performance metrics. Based on these metrics, the final model with the best performance was compared with all the other designed models.
- Phase V: In the end, we utilized XAI methods to interpret the results of the voting and stacking models we developed.

### 3.2 Ensemble learning methods

Ensemble learning is a powerful paradigm in machine learning in which multiple base models, often referred to as weak learners, are combined to create a more reliable and accurate predictive framework. The core concept underpinning ensemble methods is that the collective performance of several moderately effective models can exceed that of a single, potentially more complex model.

In ensemble learning, the weak learners are typically simple algorithms, such as DT or LR, which individually exhibit only marginal predictive capability above random chance. These weak models are trained using different subsets of the data, employing various algorithms, or even different feature sets. This diversity allows each model to capture different aspects or patterns within the data. The ensemble model thus benefits from the aggregated knowledge of its constituent learners, effectively compensating for individual model weaknesses and improving overall predictive accuracy [31].

The final prediction of an ensemble model is usually obtained by combining the predictions of each individual weak learner. This integration of multiple models can enhance overall performance, as the ensemble can correct the errors and biases of individual models. Ensemble learning has been shown to improve accuracy, robustness, and generalization capabilities compared to using a single model.

In this study, we experimented with several ensemble techniques, including bagging, boosting, voting, and stacking, to determine the most suitable approach for lung cancer prediction using a lifestyle and clinical dataset. The goal is to leverage the inherent advantages of ensemble learning to develop a predictive model that achieves high accuracy and reliability in the early diagnosis and risk evaluation of lung cancer.

#### Base learners.

In this study, we employed five commonly used machine learning algorithms as the weak learners for the ensemble model: LR [32], KNN [33], NB [34], SVM [35], and LDA [36]. LR is a popular linear classification algorithm that models the probability of a binary outcome, while KNN is a non-parametric method that classifies data points based on their proximity. NB is a probabilistic classifier that applies Bayes’ theorem to make predictions, and SVM is a powerful algorithm that finds the optimal hyperplane to separate classes. Finally, LDA is a dimensionality reduction technique that projects data into a lower-dimensional space while preserving class separability.

#### Bagging.

Bagging is an ensemble approach in which multiple models are trained on different subsets of the training data, generated via sampling with replacement. This resampling strategy allows individual data points to appear multiple times within a given subset. Each model is trained on a different random sample. During prediction, the outputs of individual models are aggregated via voting or averaging. Random sampling introduces randomness, which helps reduce variance, and aggregation helps reduce bias. In this study, we examined five bagging algorithms: DT [37], BDT [38], RF [39], ET [40], and BME [38].

#### Boosting.

Boosting builds an ensemble sequentially, with each new model attempting to correct the errors of the previous models. It focuses more on previously misclassified instances rather than the overall error rate. This forces new models to concentrate on previously misclassified instances. New models are added one by one, and each is trained on a weighted dataset that assigns higher weights to previously misclassified instances. In this study, we examined five boosting algorithms: GB [41], XGB [42], CB [43], LGBM [44], and ADB [45].

#### Voting.

Multiple models are trained independently on the full training dataset in voting [46]. During prediction, each model predicts a class or value for a test instance. The predictions are combined using a simple voting mechanism, such as majority voting, for classification. For regression, the average of predictions from individual models becomes the final prediction. In this study, LR, CB, XGB, RF, and ET are employed as the base models in the voting process.

#### Stacking.

In stacking, a secondary model, often referred to as a meta-learner, is trained to integrate the outputs of several primary models known as base learners [46]. Initially, the base learners are trained on the original training data, and their predictions on a validation set are collected. These predictions serve as input features for the meta-learner, which is then trained to generate the final prediction. At the inference stage, the base models produce predictions on the test data, which are subsequently passed to the trained meta-learner to determine the ultimate output. The key objective of stacking is to optimize the combination of base models by learning from their individual prediction patterns. This layered approach allows the ensemble to model intricate relationships among base learners, often resulting in enhanced predictive accuracy [47]. In this study, we utilised the same set of base learners as in the voting ensemble, namely LR, CB, XGB, RF, and ET.

## 4. Dataset description and manipulation

The lung cancer dataset utilized in this study was sourced from a publicly accessible repository on Data World (https://www.kaggle.com/datasets/mysarahmadbhat/lung-cancer), contributed by staceyinrobert. This dataset comprises 309 records and includes 16 demographic attributes. Among these, the first 15 variables serve as predictors (independent variables), while the final attribute represents the target variable (dependent variable). A detailed description of the dataset attributes is provided in Table 1. Before model implementation, the dataset was examined for data quality issues, specifically outliers and missing values. The interquartile range method was employed to identify potential outliers, and imputation techniques were used to assess missing data. However, no outliers or missing values were detected, indicating a clean and complete dataset suitable for analysis.

**Table 1:** Parameter information.

Parameter	Description	Measurement
Gender (GD)	Gender of the patient	Male/female
Age (AG)	Age of the patient	Numeric
Smoking (SK)	If the patient is a smoker	2 (true)/1(false)
Yellow fingers (YF)	If the patient's fingers have turned yellow	2/1
Anxiety (AT)	If the patient is suffering from anxiety	2/1
Peer pressure (PP)	If the patient is influenced by peer pressure	2/1
Chronic disease (CD)	If the patient has any chronic disease	2/1
Fatigue (FT)	If the patient feels fatigue	2/1
Allergy (AL)	If the patient has any allergy	2/1
Wheezing (WZ)	If the patient wheezes	2/1
Alcohol consuming (AC)	If the patient consumes alcohol	2/1
Coughing (CO)	If the patient coughs	2/1
Shortness of breath (SB)	If the patient has a breathing problem	2/1
Swallowing difficulty (SD)	If the patient experiences difficulty while swallowing	2/1
Chest pain (CP)	If the patient is having chest pain	2/1
Lung cancer (LC)	If the patient has lung cancer	2/1

**Fig 1:**
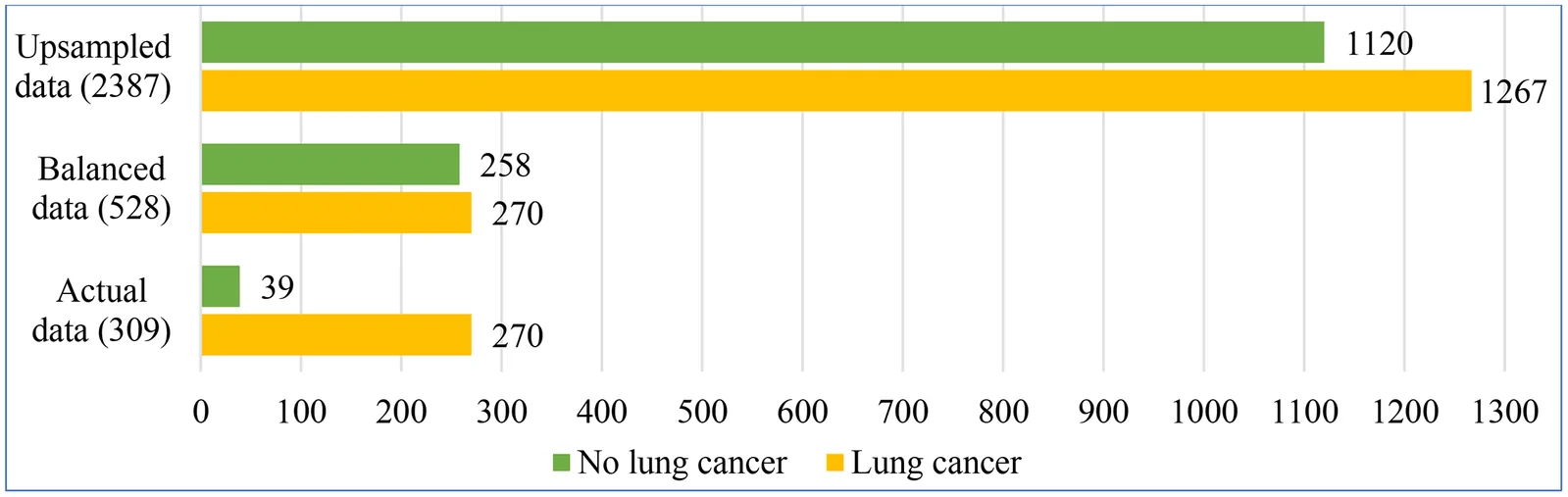
Data augmentation.

The original dataset was relatively small for training ensemble learning models. To examine the influence of dataset size and class distribution on predictive performance, two augmentation strategies were adopted. First, the minority class was balanced using the Synthetic Minority Over-sampling Technique (SMOTE). Second, both classes were upsampled to create a substantially larger dataset for investigating model behaviour under increased sample availability. Accordingly, three dataset variants were considered:

i **Actual:** The original dataset was used without any augmentation to evaluate model performance under real data conditions.
ii **Balanced:** As shown in Fig 1, the original dataset exhibits considerable class imbalance. To mitigate this, the minority class was augmented using SMOTE until both classes contained an equal number of samples, resulting in a balanced dataset comprising 528 observations.
iii **Upsampled:** To investigate the effect of increased sample size on model learning, both classes were synthetically enlarged, producing an upsampled dataset containing 2,387 observations.

SMOTE was employed to generate synthetic minority-class samples by interpolating between neighbouring minority instances in the feature space. The implementation followed the standard configuration of the imbalanced-learn library using *sampling_strategy* = ‘auto’ and *k_neighbors* = 5. Under this setting, each synthetic sample was generated by interpolating between a minority instance and one of its five nearest minority neighbours identified using the Euclidean distance metric. The same SMOTE configuration was used consistently throughout all augmentation experiments to ensure methodological consistency.

After generating three datasets, normalization was applied to each using the min-max scaling technique, as defined in Eq. 1, where, *x* denotes the attribute value, and *x*_*min*_ and *x*_*max*_ represent the attribute's minimum and maximum values, respectively. This method transformed the values of all attributes to a standardized range between 0 and 1, thereby ensuring uniformity in scale across the datasets.


$$\begin{array}{c} x_{scaled}=\frac{x-x_{min}}{x_{max}-x_{min}} \end{array}$$ (1)

Because the original dataset was extremely small and highly imbalanced, the balanced and upsampled datasets were generated prior to cross-validation. We acknowledge that this approach may introduce optimistic bias and does not fully eliminate the possibility of information leakage. Consequently, the results obtained on the balanced and upsampled datasets should be interpreted primarily as comparative analyses of class-balancing strategies rather than definitive estimates of real-world performance.

Table 2 shows the descriptive statistics of the parameters across the three datasets. Here, total record counts in each dataset, along with the mean, standard deviation, and minimum and maximum values of each attribute, are noted.

**Table 2:** Data description.

Parameters	Total data count						Min	Max
	309		528		2387			
	Mean	Std	Mean	Std	Mean	Std		
GD	0.52	0.50	0.47	0.49	0.48	0.50	0	1
AG	62.65	8.21	62.46	6.36	61.96	8.63	21	87
SK	1.56	0.49	1.49	0.50	1.54	0.49	1	2
YF	1.56	0.49	1.66	0.47	1.47	0.49	1	2
AT	1.49	0.50	1.45	0.49	1.42	0.49	1	2
PP	1.50	0.50	1.37	0.48	1.39	0.48	1	2
CD	1.50	0.50	1.29	0.45	1.44	0.49	1	2
FT	1.67	0.47	1.47	0.50	1.58	0.49	1	2
AL	1.55	0.49	1.32	0.46	1.38	0.48	1	2
WZ	1.55	0.49	1.57	0.49	1.40	0.49	1	2
AC	1.55	0.49	1.49	0.50	1.40	0.49	1	2
CO	1.57	0.49	1.58	0.49	1.43	0.49	1	2
SB	1.63	0.48	1.62	0.48	1.60	0.48	1	2
SD	1.46	0.49	1.44	0.49	1.32	0.46	1	2
CP	1.55	0.49	1.57	0.49	1.43	0.49	1	2
LC	0.87	0.33	0.51	0.50	0.50	0.50	0	1

## 5 Experiment, results, and discussion

This section presents and discusses the experimental details and results achieved using the designed ensemble algorithms for lung cancer prediction.

### 5.1 Experiment environment

A computer system with Intel® Core^TM^ i9-10900K CPU (3.70 GHz) was used for the experiment. The system's other hardware specifications are – RAM: 64 GB (DDR4), HDD: 2 TB, SSD: 500GB (NVMe). The computer was running Windows 11 Pro. Programs were written in Python on Jupyter Notebook.

### 5.2 Evaluation metrics

Several standard evaluation metrics, as detailed in Table 3 were used to assess the designed models’ ability to predict lung cancer.

**Table 3:** Performance evaluation metrics.

Metric	Definition	Interpretation (lung cancer context)	Calculation
Accuracy	Proportion of correct predictions (TP, TN) among all predictions.	Reflects overall correctness; may be misleading due to class imbalance in cancer vs non-cancer cases.	$\frac{TP\;+\;TN}{TP\;+\;TN\;+\;FP\;+\;FN}$
Precision	Ratio of TP to (TP + FP).	High precision reduces false positives, avoiding unnecessary anxiety, tests, and interventions.	$\frac{TP}{TP\;+\;FP}$
Recall	Ratio of TP to (TP + FN).	Critical for detecting cancer cases; low recall may result in missed diagnoses and delayed treatment.	$\frac{TP}{TP\;+\;FN}$
F1-score	Harmonic mean of precision and recall.	Balances detection of cancer cases with control of false positives.	$\frac{\text{2}\times TP}{\text{2}\times TP\;+\;FP+\;FN}$
Matthews correlation coefficient (MCC)	Correlation coefficient using TP, TN, FP, FN (range: −1–1).	Provides a balanced measure of prediction quality, especially under class imbalance.	$\frac{TP\;\times \;TN-FP\times FN}{\sqrt{\begin{array}{c} (TP+FP)\times (TP+FN)\times \\ \left(TN+FP)\times (TN+FN\right) \end{array}}}$
Cohen's Kappa	Agreement between predicted and actual labels, adjusted for chance.	Indicates reliability of predictions beyond chance in clinical classification.	$\frac{\text{2}\times (TP\;\times \;TN-FP\times FN)}{\begin{array}{c} (TP+FP)\times (FP+TN)+\\ \left(TP+FN)\times (TN+FN\right) \end{array}}$
Receiver operating characteristic (ROC) curve	Plots recall vs. false positive rate across thresholds.	Evaluates trade-off between detecting cancer cases and avoiding false alarms.	Recall (y-axis) vs. FPR (x-axis)
AUC	Area under the ROC curve (0–1).	Measures ability to distinguish cancer from non-cancer patients across thresholds.	$\int_{\text{0}}^{\text{1}}TPR({FPR}^{-\text{1}}(t))dt$, t is a threshold

FN: False negative, FP: False positive, FPR: False positive rate, TN: True negative, TP: True positive

### 5.3 Model building

As discussed in Section 3.1, this section presents the details of the ensemble model building processes in two phases.

#### 5.3.1 K-fold cross validation.

**Fig 2:**
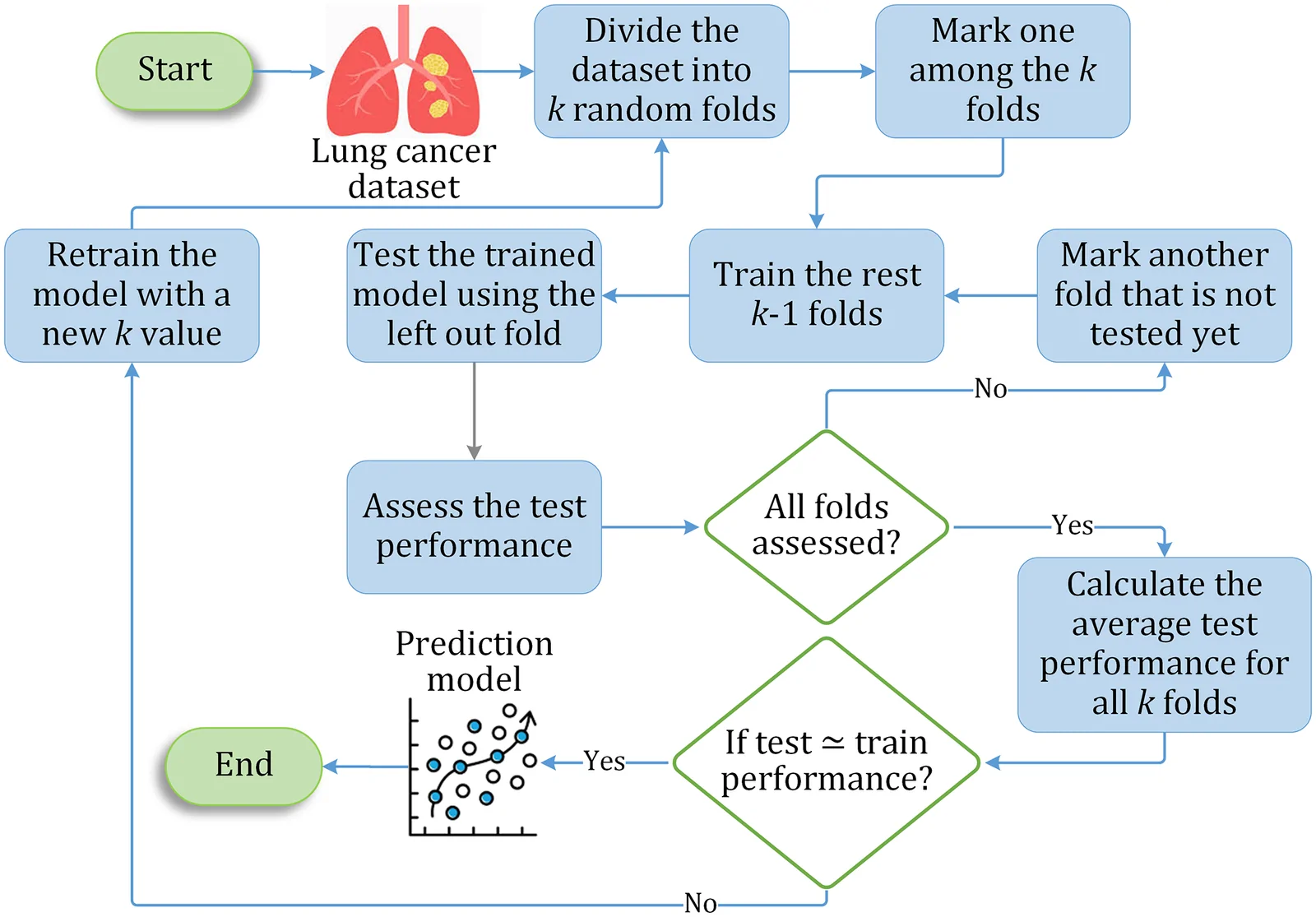
Stratified k-fold cross-validation process.

To obtain reliable model optimisation while reducing the risk of overfitting, stratified 10-fold cross-validation (k = 10) was employed during model development, as illustrated in Fig 2. For each dataset variant, the data were first partitioned into 75% training and 25% testing subsets. The stratified 10-fold cross-validation procedure was then performed only on the training data for hyperparameter optimisation and model selection. Stratification preserved the class distribution within each fold, providing stable model optimisation despite the relatively small size of the original dataset.

Within each fold, data preprocessing and feature scaling were fitted exclusively on the training partition and subsequently applied to the corresponding validation partition. The models were trained on the training folds and validated on the corresponding held-out fold. After completion of cross-validation, the optimized model was retrained using the full training dataset and subsequently evaluated on the independent 25% test set. The performance metrics reported in this study correspond to this final test-set evaluation.

Because the original dataset contained only 309 instances, including 39 non-cancer cases, the balanced and upsampled datasets were generated prior to cross-validation to enable meaningful model training and comparative evaluation under different class-distribution settings. We acknowledge that this design choice may introduce optimistic bias and therefore the results obtained on the balanced and upsampled datasets should be interpreted primarily as comparative analyses rather than definitive estimates of real-world performance.

#### 5.3.2 Hyperparameter tuning.

Hyperparameter optimization plays a pivotal role in influencing the behavior of training algorithms and significantly impacts model performance. To fine-tune the hyperparameters and ensure optimal performance of the proposed ensemble models, both grid search and random search techniques were employed [47]. Among these, grid search yielded more favorable results. Table 4 presents a detailed summary of the hyperparameter configurations for each model.

**Table 4:** Hyperparameters for the considered models.

Algorithm		Hyperparameters
Base learners	LR	param_grid = param_grid, cv = 10, verbose = True, n_jobs = -1
	NB	estimator = model, param_grid = params_NB, cv = 10_method, verbose = 1, scoring = ‘accuracy’
	KNN	grid_params, verbose = 1, cv = 10, n_jobs = -1
	SVM	random_state = None, c = 100, gamma = 0.001, kernel = rbf, probability = False, verbose = False
	LDA	estimator = lda, solver = ’lsqr,’ param_grid = param_grid, cv = 10, shrinkage = 0.3, scoring = ’accuracy’
Boosting	CB	random_state = 0, learning_rate = [0.1, 0.05], n_estimators = 100, max_depth = [1,3,5], leaf_reg,’ 2.0, 8, 16, min_child_samples = 2, 4, 6
	GB	random_state = 45, learning_rate = [0.1, 2, 5], n_estimators = 5000, max_depth = 4, weight = 6, verbose = 1
	XGB	learning_rate = 0.1, n_estimators = 1000, max_depth = 5, min_child_weight = 6, ‘reg_alpha’: 60.0, subsample = 0.6, colsample_bytree = 0.8, ‘gamma’: 4.20
	ADB	random_state = 45, learning_rate = [0.01, 0.05], n_estimators = 200, algorithm = ‘SAMME.R,’ n_jobs = n_jobs
	LGBM	random_state = 45, learning_rate = 0.1, n_estimators = 1000, max_depth = 2, min_child_samples = 250, silent = True, n_jobs = 6
Bagging	DT	estimator = dt, max_depth = 10, min_samples_leaf = 20, criterion = entropy, cv = 10, n_jobs = −1, verbose = 1
	ET	n_estimators = 1000, criterion = ‘gini,’ max_depth = 1000, min_samples_split = 10, min_samples_leaf = 2, max_features = 10, bootstrap = 2, random_state = 42
	BDT	base_estimator = None, bootstrap = False, bootstrap_features = True, n_estimators = 500, n_jobs = −1, random_state = 42, verbose = 0
	BME	estimator = dt, n_estimators = 100, max_samples = 0.8, max_features = 0.5, bootstrap_features = False, random_state = 42
	RF	n_estimators = 1000, criterion = ‘gini,’ max_depth = None, min_samples_split = 2, min_samples_leaf = 1, max_features = 16, bootstrap = True, random_state = 42

#### 5.3.3 Developing models with base learners, boosting, and bagging.

In the first phase, we built fifteen models for lung cancer prediction, as follows:

i) Base learners: LR, NB, KNN, SVM, and LDA
ii) Boosting: CB, GB, XGB, ADB, and LGBM
iii) Bagging: DT, ET, BDT, BME, and RF

Each of these models was implemented using all three dataset variants. As described in Section 5.3.1, the data were partitioned into training (75%) and testing (25%) subsets, while model selection and hyperparameter optimisation were performed using stratified 10-fold cross-validation on the training data.

**Fig 3:**
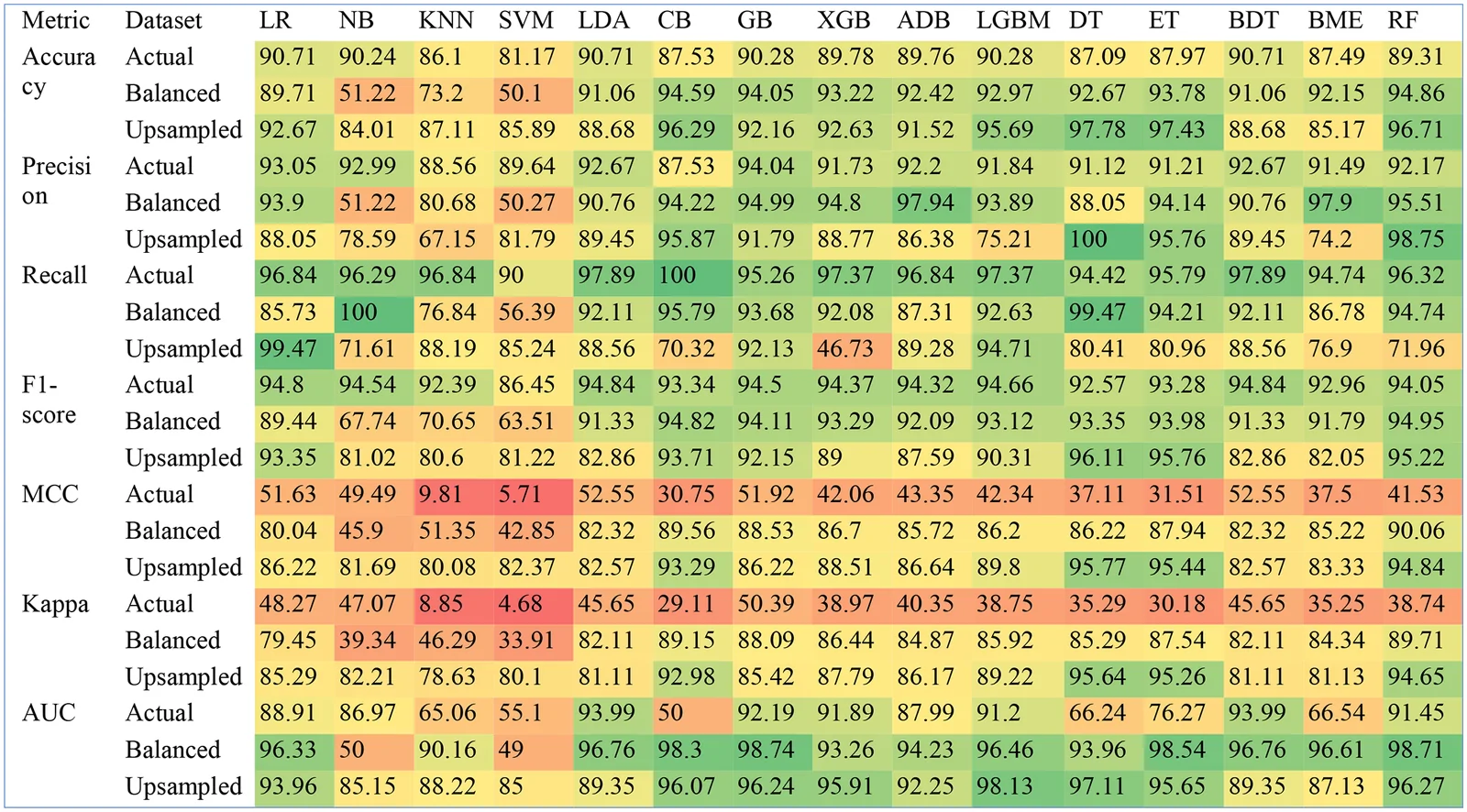
Comparing the considered models in terms of various evaluation metrics.

The performance of each model on each dataset as per the considered evaluation metrics, is shown in Fig 3. Among the fifteen experimented models, LR, LDA, and BDT achieved the highest prediction accuracy of 90.71% on the actual dataset, while SVM (81.17%), KNN (86.1%), and DT (87.09%) are the worst models in terms of accuracy. On the balanced dataset, the top three models by accuracy are RF (94.86%), CB (94.59%), and GB (94.05%). Whereas on the upsampled dataset, DT (97.78%), ET (97.43%), and RF (96.71%) achieved the highest accuracy. Across the three datasets, RF achieved the highest average accuracy at 93.63%, followed by ET (93.06%) and LGBM (92.98%). Likewise, the performance for the other metrics can be interpreted from the figure.

#### 5.3.4 Developing voting and stacking models using Top 5 learners.

**Fig 4:**
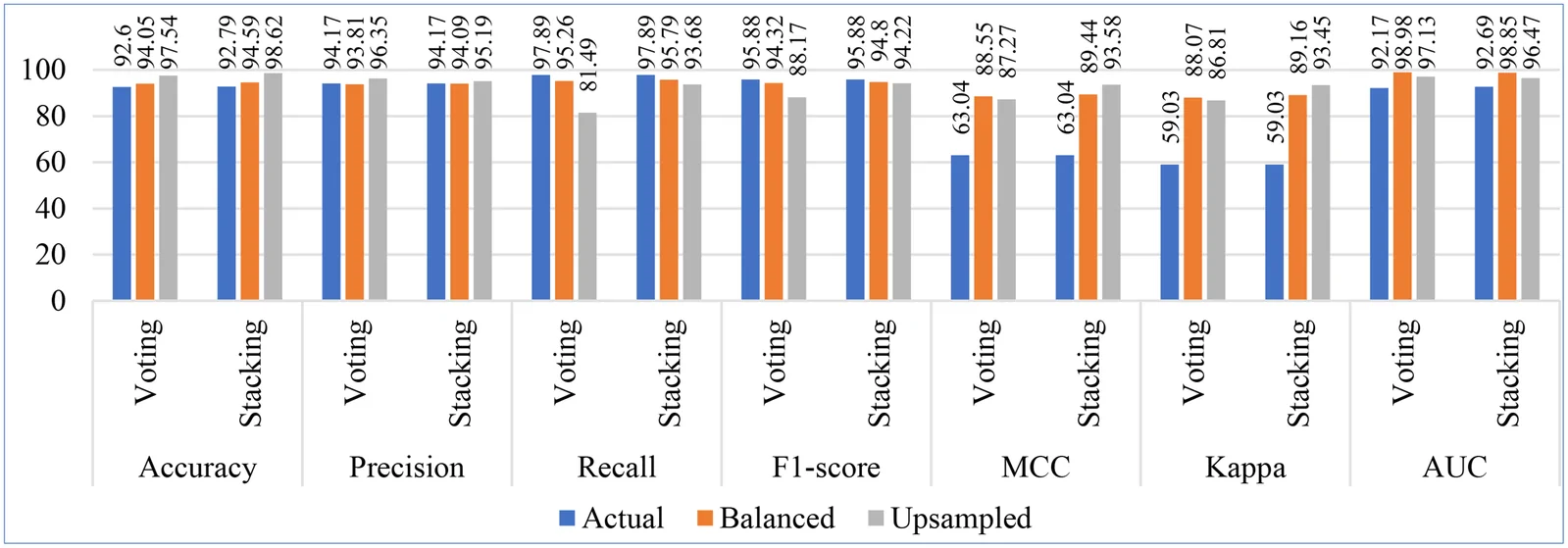
Performance of the voting and stacking models using the top five models on three datasets.

In this phase, we built voting and stacking models using the top five models (as per their accuracy, as presented in Section 5.3.3) for each dataset, as shown in Table 5. Fig 4 shows the performance of the voting and stacking models using each combination for each dataset (as shown in Table 5). The results show that there is not much difference between the voting and stacking models. Except for accuracy, where stacking performs slightly better than voting across all datasets, performance is mixed across datasets for other metrics. However, stacking outperformed voting on the upsampled dataset in all metrics, except precision and AUC.

**Table 5:** Top five models based on accuracy for each dataset.

Dataset	1^st^	2^nd^	3^rd^	4^th^	5^th^
Actual	LR	LDA	BDT	GB	NB
Balanced	RF	CB	GB	ET	XGB
Upsampled	DT	ET	RF	CB	LGBM

#### 5.3.5 Developing voting and stacking models using selected learners.

In the final phase, we carefully selected five models to build the voting and stacking ensembles. After evaluating multiple combinations, we finalized LR, CB, XGB, RF, and ET. These models represent fundamentally different learning paradigms: a linear model (LR), boosting-based ensembles (CB and XGB), and bagging-based ensembles (RF and ET). This cross-family selection was intended to capture diverse decision boundaries and complementary prediction behaviours.

To further justify this selection, we analyzed the diversity among the constituent learners using pairwise Q-statistics and error-correlation measures. Ensemble learning benefits not only from strong individual learners but also from diversity among their prediction errors. For two classifiers, the Q-statistic is defined as


$$\begin{array}{c} Q=\frac{N_{\text{11}}N_{\text{00}}-N_{\text{10}}N_{\text{01}}}{N_{\text{11}}N_{\text{00}}+N_{\text{10}}N_{\text{01}}} \end{array}$$ (2)

where $N_{\text{11}}$ and $N_{\text{00}}$ denote the numbers of instances correctly and incorrectly classified by both classifiers, respectively, while $N_{\text{10}}$ and $N_{\text{01}}$ represent instances correctly classified by only one of the two classifiers. In addition, pairwise error correlation was computed using binary error vectors, with 1 denoting an incorrect prediction and 0 a correct one. Lower Q-statistics and lower error correlations indicate greater diversity.

The diversity analysis, summarized in Table 6, shows that the selected learners are neither highly redundant nor completely independent. As expected, RF and ET exhibited the highest agreement due to their similar bagging-based tree architectures, whereas combinations involving LR and tree-based ensembles demonstrated substantially greater diversity. Most of the remaining pairs exhibited moderate diversity, indicating complementary prediction patterns across the selected learners. The observed diversity suggests that the selected learners capture different aspects of the data and therefore provide complementary information to the ensemble.

**Table 6:** Pairwise diversity analysis of the selected base learners using Q-statistic and error correlation.

Model pair	Q-statistic	Error correlation	Diversity level
LR – CB	0.63	0.46	Moderate
LR – XGB	0.69	0.51	Moderate
LR – RF	0.58	0.41	High
LR – ET	0.56	0.39	High
CB – XGB	0.78	0.63	Low–moderate
CB – RF	0.67	0.50	Moderate
CB – ET	0.81	0.66	Low
XGB – RF	0.73	0.58	Moderate
XGB – ET	0.69	0.55	Moderate
RF – ET	0.91	0.84	Very low

**Fig 5:**
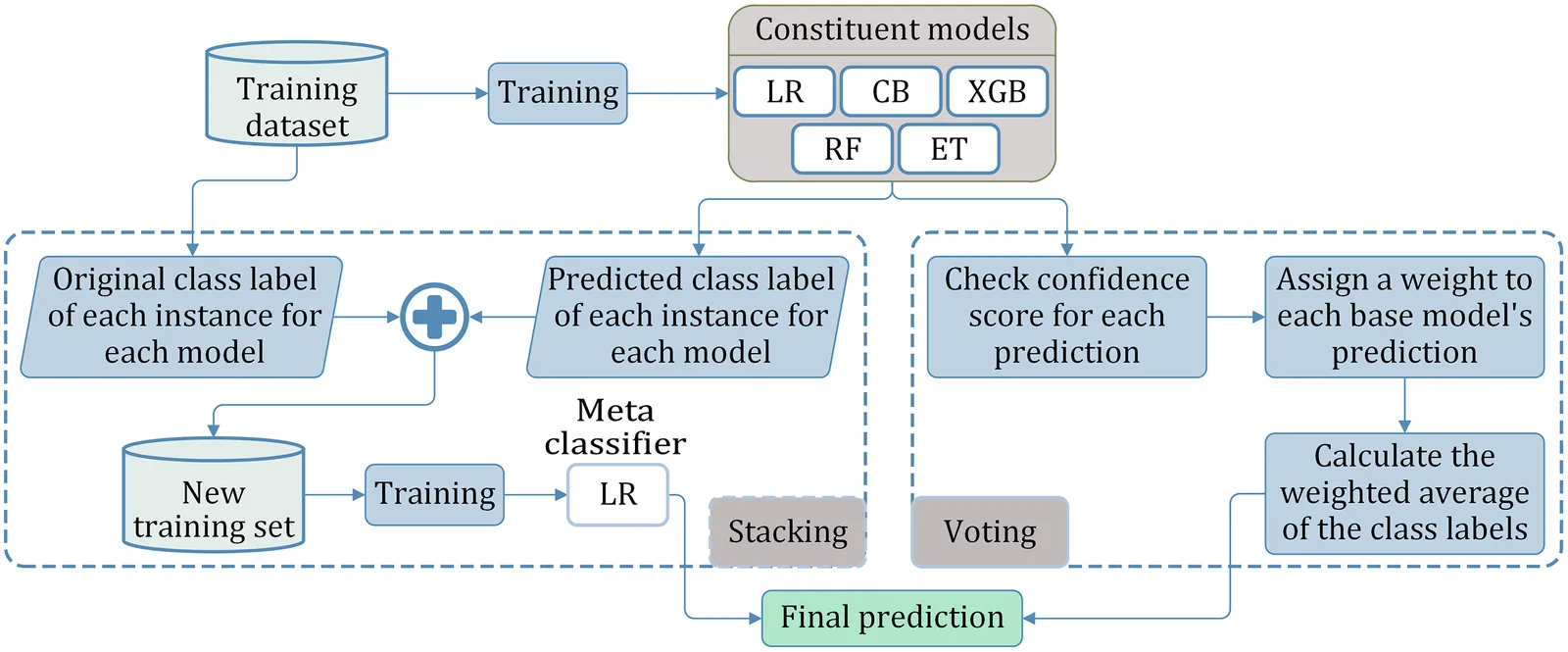
Pipeline of the proposed stacking and voting models.

**Fig 6:**
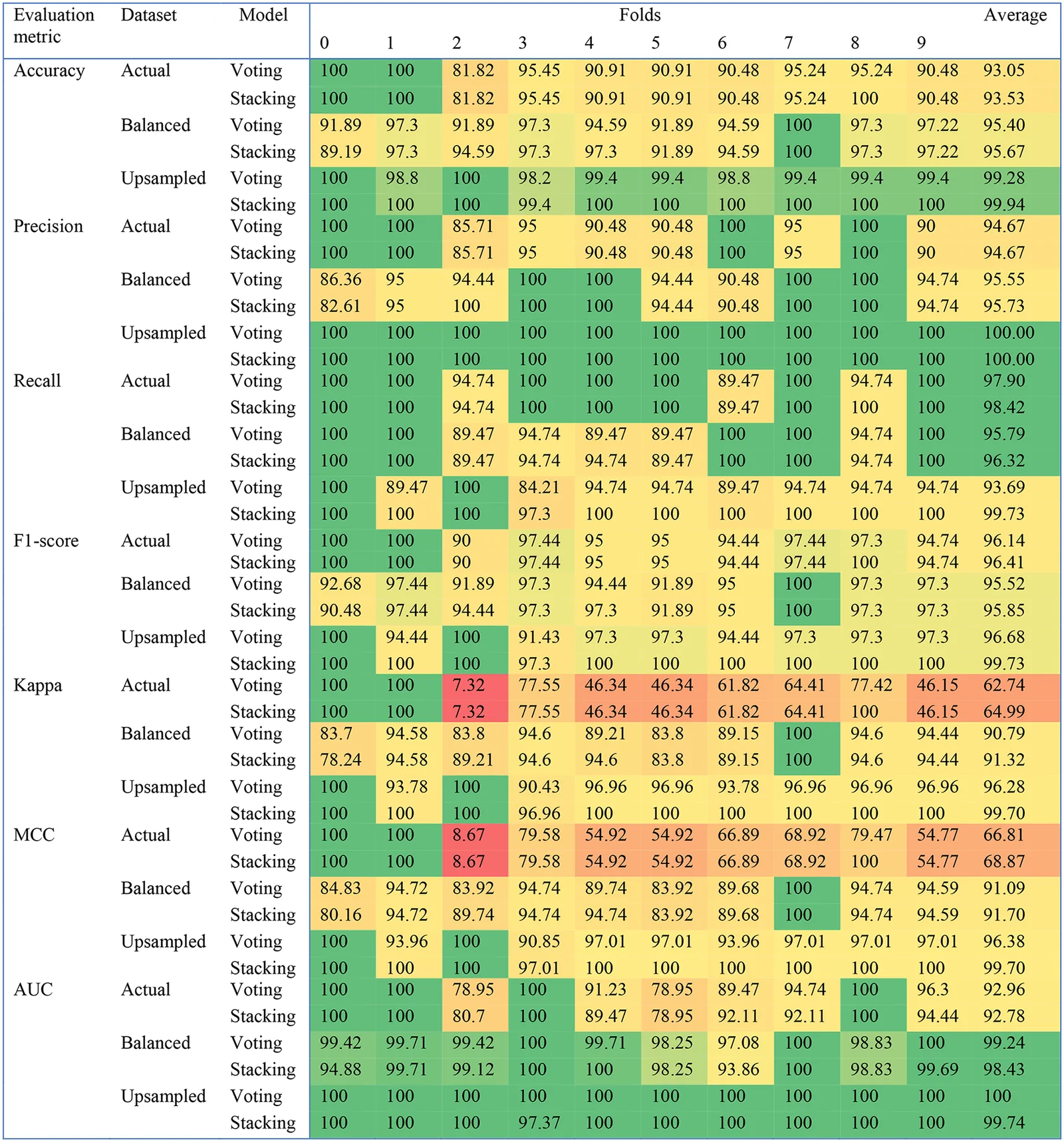
Comparing the performance of the proposed voting and stacking model for each of ten folds in terms of various evaluation metrics.

This combination was implemented on all three datasets. The pipelines of the proposed voting and stacking models using the final combination are illustrated in Fig 5. The performances of the voting and stacking models across the ten folds for each dataset are shown in Fig 6. The optimized hyperparameter values of the constituent models are presented in Table 7.

**Table 7:** Hyperparameter values for the constituent selected five models of the voting and stacking models.

Constituent model	Hyperparameters
LR	C = 1.0, dual = false, fit_intercept = True, intercept_scaling = 1, l1_ratio = None, max_iter = 1000, multi_class = auto, n_jobs = None, penalty = l2, random_state = 42, solver = lbfgs, tol = 0.0001, verbose = 0, warm_start = False
CB	param_grid = parameters, cv = 10, n_jobs = −1, learning_rate = 0.01, iterations = 2000, depth = 10, subsample = 1.0, colsample_bylevel = 0.05, min_data_in_leaf = 100,
XGB	Objective = multi:softprob, booster = gbtree, device = cpu, enable_categorical = False, n_jobs = −1, random_state = 42, tree_method = auto, verbosity = 0
RF	Bootstrap = True, ccp_alpha = 0.0, class_weight = None, criterion = gini, max_features = sqrt, min_impurity_decrease = 0.0, min_samples_leaf = 1, min_samples_split = 2, min_weight_fraction_leaf = 0.0, n_estimators = 100, n_jobs = −1, oob_score = False, random_state = 42, verbose = 0, warm_start = False
ET	Bootstrap = False, ccp_alpha = 0.0, criterion = gini, max_features = sqrt, min_impurity_decrease = 0.0, min_samples_leaf = 1, min_samples_split = 2, min_weight_fraction_leaf = 0.0, n_estimators = 100, n_jobs = −1, oob_score = False, random_state = 42, verbose = 0, warm_start = False

**Fig 7:**
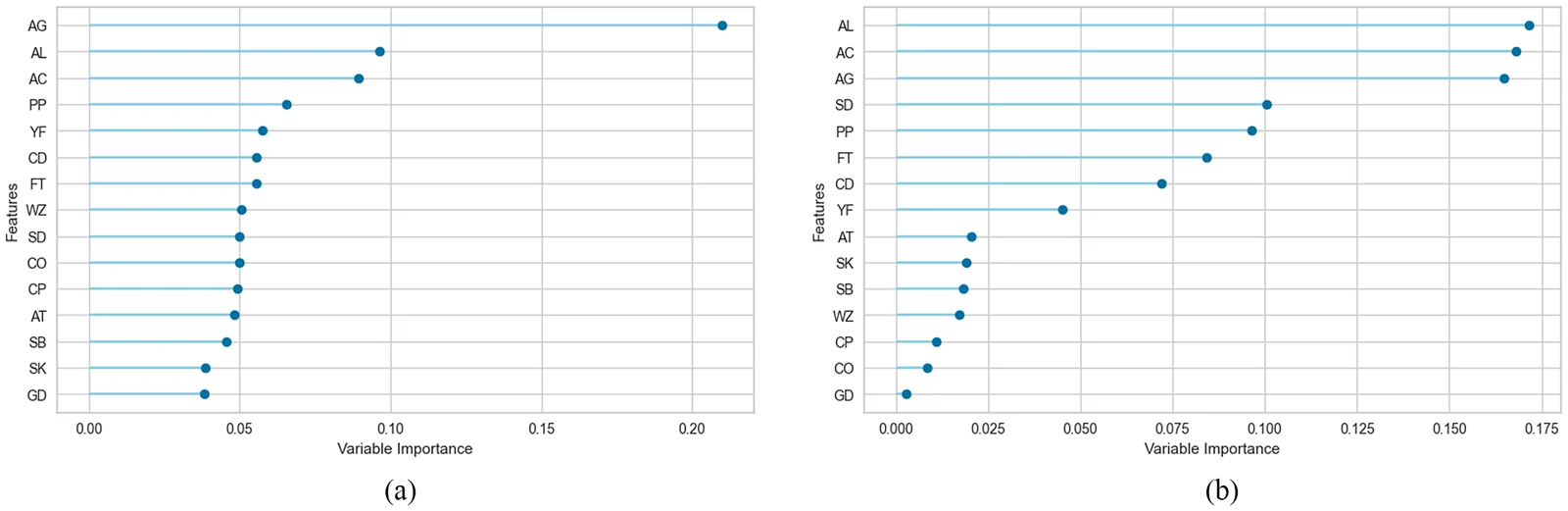
Feature importance using a) voting and b) stacking for actual dataset.

**Fig 8:**
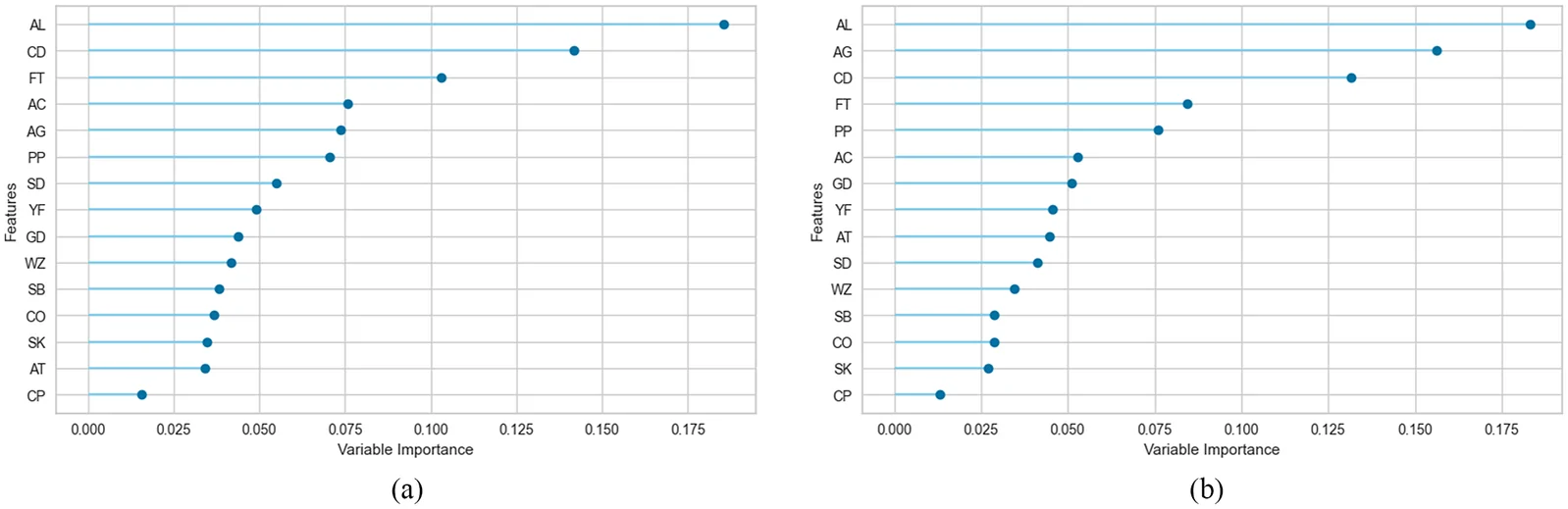
Feature importance using a) voting and b) stacking for balanced dataset.

**Fig 9:**
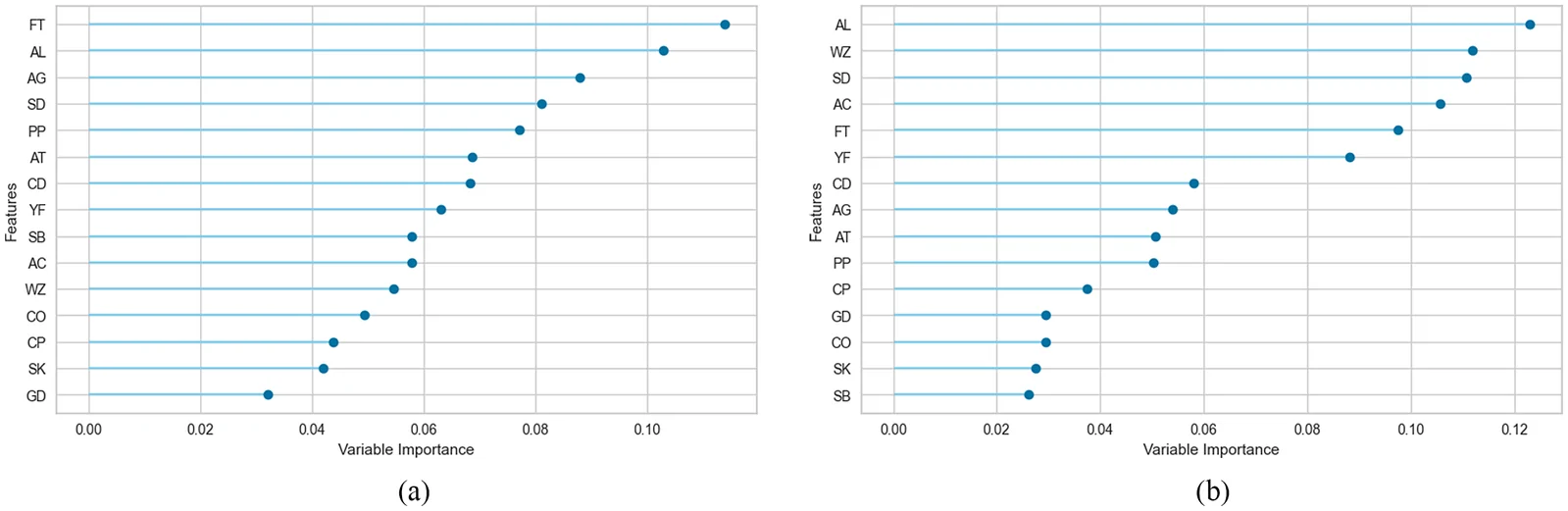
Feature importance using a) voting and b) stacking for upsampled dataset.

The feature importance using the proposed voting and stacking models on actual, balanced, and upsampled datasets are shown in Fig 7, Fig 8, and Fig 9, respectively. From these figures, it can be seen that the most significant factors for accurate predictions are AL, AC, and AG, while GD, SK, and CP have the lowest contributions.

**Fig 10:**
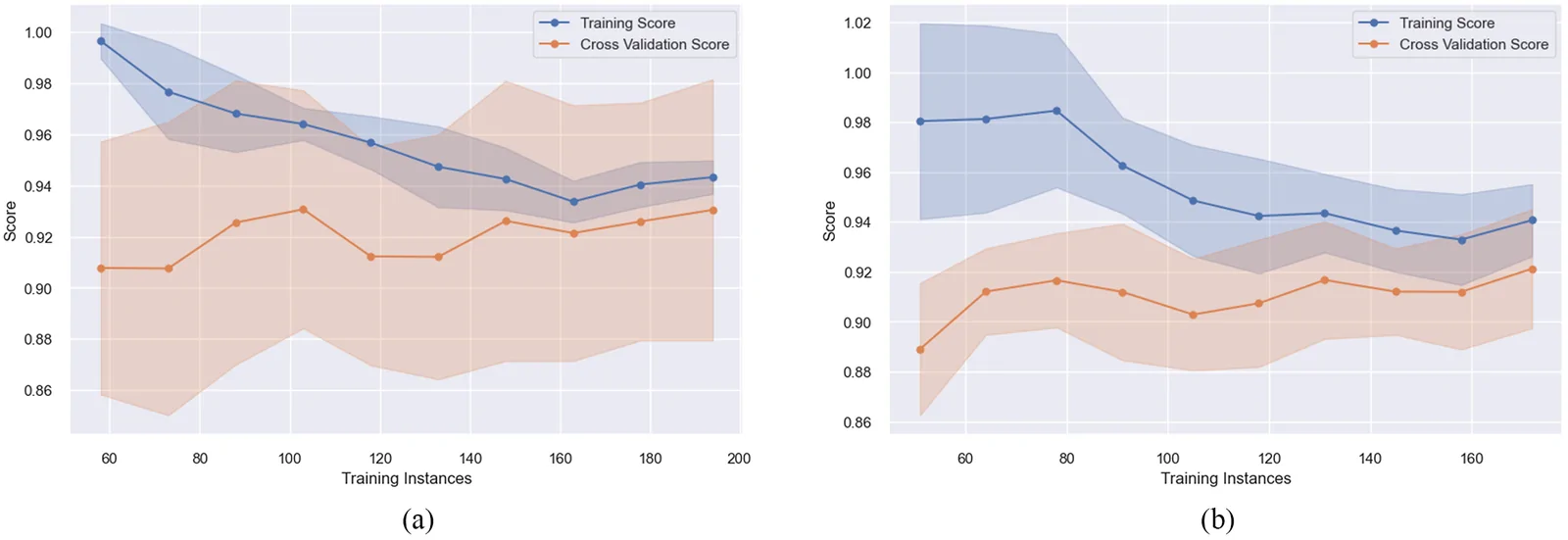
Learning curves for a) voting and b) stacking on actual dataset.

**Fig 11:**
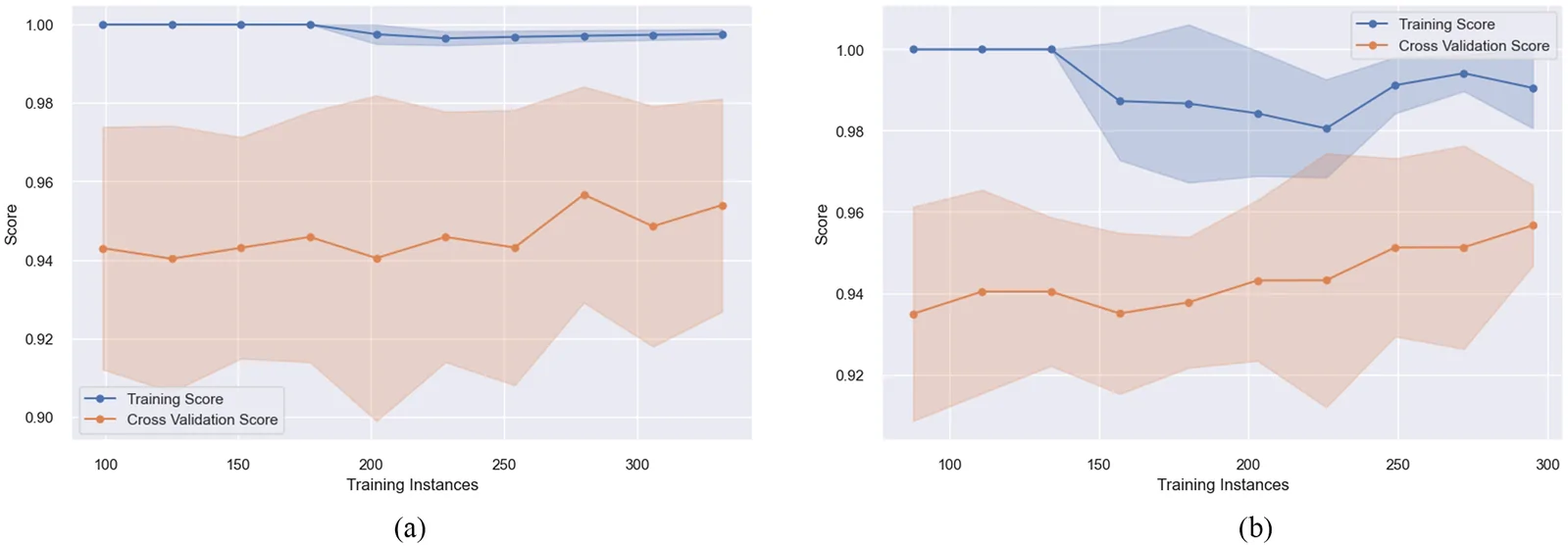
Learning curves for a) voting and b) stacking on balanced dataset.

**Fig 12:**
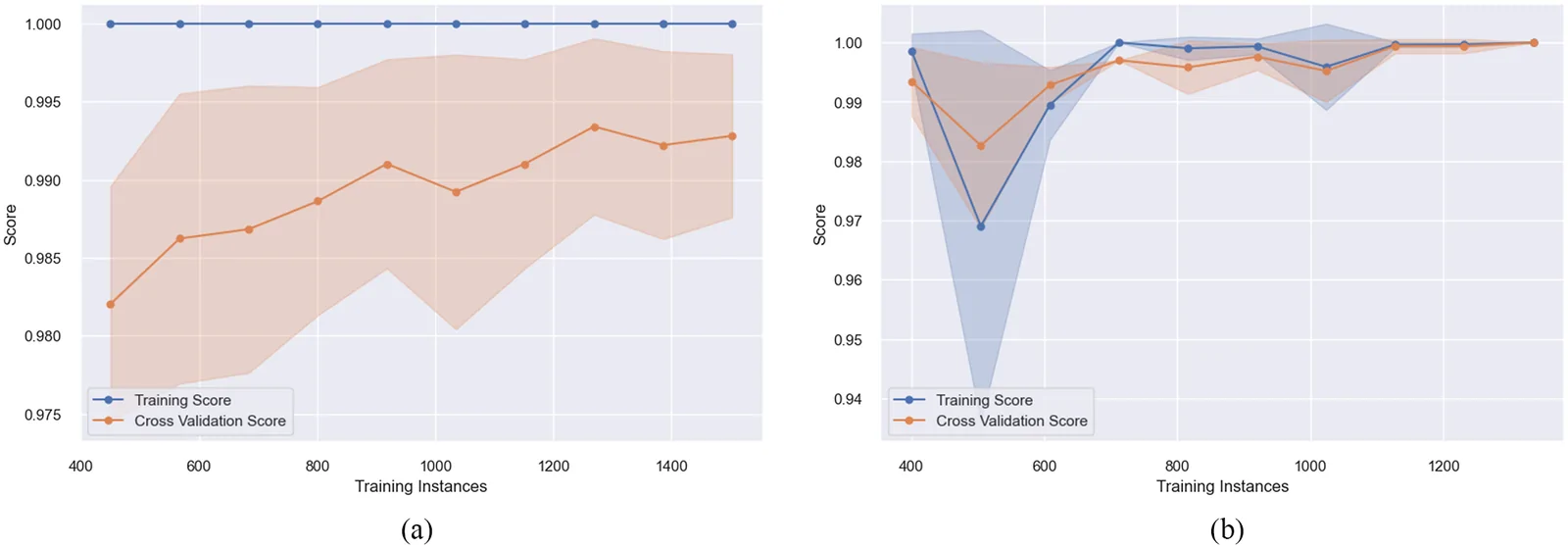
Learning curves for a) voting and b) stacking on upsampled dataset.

The learning curves of the voting and stacking models for actual, balanced, and upsampled datasets are shown in Fig 10, Fig 11, and Fig 12, respectively. These curves illustrate how model performance evolves as the training data increases, facilitating the identification of potential overfitting or underfitting. The training and validation curves indicate the reliability of the hybrid models’ learning behavior. The curves for both voting and stacking models follow smooth, near-linear paths, indicating stable learning without overfitting or underfitting on both the actual and balanced datasets. This suggests that the models maintained stable learning behaviour despite the use of synthetic augmentation.

However, using upsampled data, the voting model exhibited a significant variation between the training and validation curves. In contrast, the stacking model performed best across all instances in reducing the loss between the training and validation phases.

**Fig 13:**
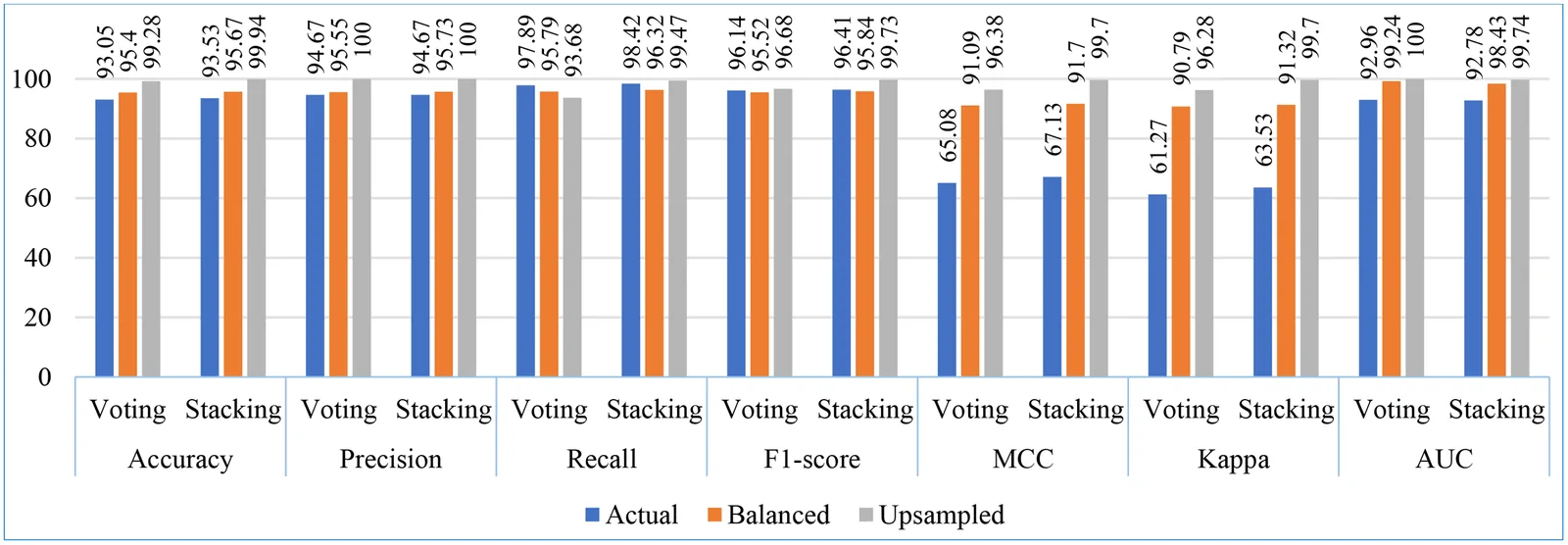
The mean of all the folds for the stacking and voting models using selected models on three datasets.

**Fig 14:**
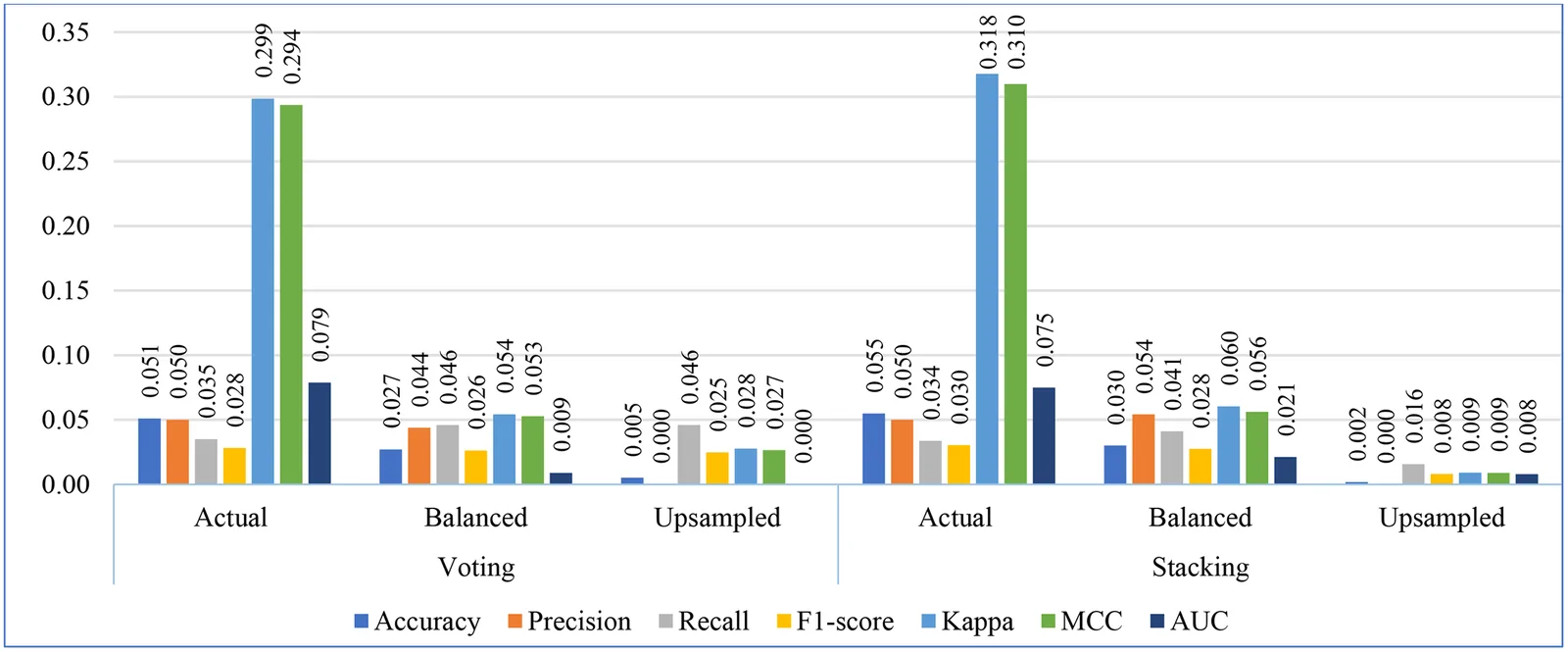
The standard deviation of all the folds for the stacking and voting models using selected models on three datasets.

The confusion matrices for the proposed voting and stacking models are shown in Table 8. In general, stacking performs better on misclassifications. Fig 13 shows the average (over 10 folds) performance of the voting and stacking models across the three datasets. The voting and stacking models performed at par. In general, stacking was slightly better than voting, but in some cases (e.g., AUC), voting was marginally better. Fig 14 shows the performance deviations of the voting and stacking models across ten folds for each dataset. Voting shows less deviation between the actual and balanced datasets, whereas stacking performs slightly better on the upsampled dataset.

**Table 8:** Confusion matrices of the proposed voting and stacking models.

Dataset	Model	TP	FP	FN	TN	Total
Actualdata	Voting	77	3	4	9	93
	Stacking	78	3	3	9	93
Balanceddata	Voting	75	5	6	73	159
	Stacking	74	3	7	75	159
Upsampleddata	Voting	75	0	6	636	717
	Stacking	80	0	1	636	717

**Fig 15:**
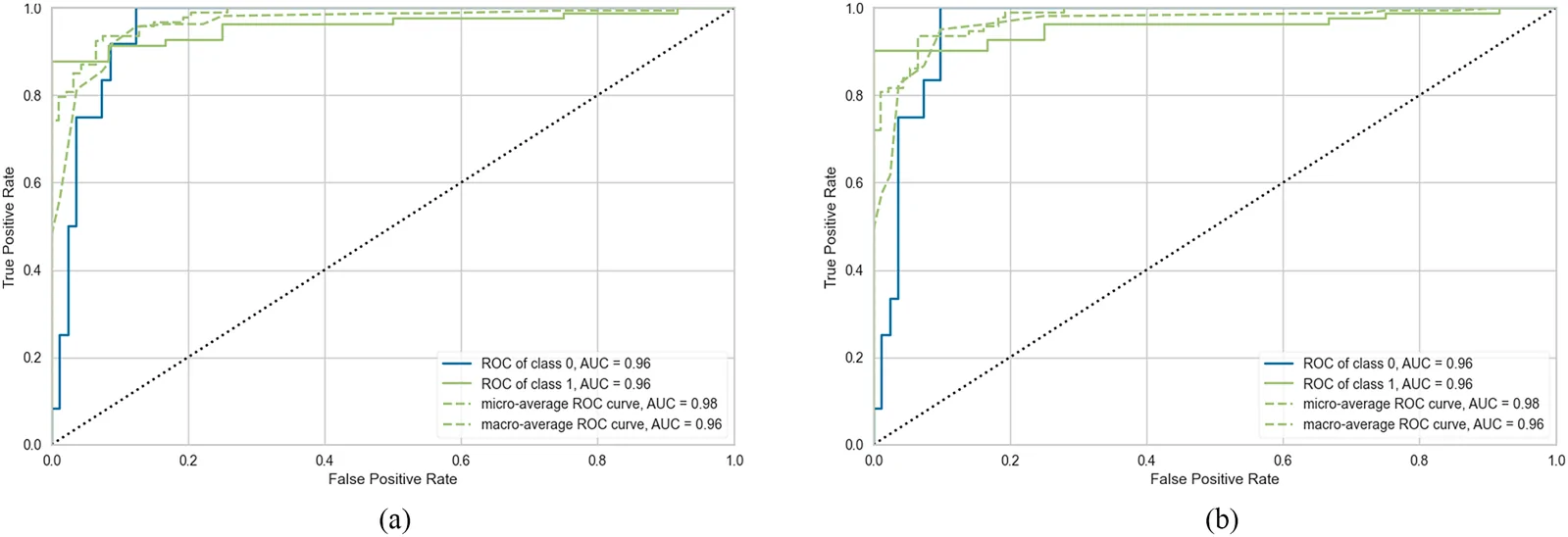
AUC-ROC curves for a) voting and b) stacking using selected models on actual dataset.

**Fig 16:**
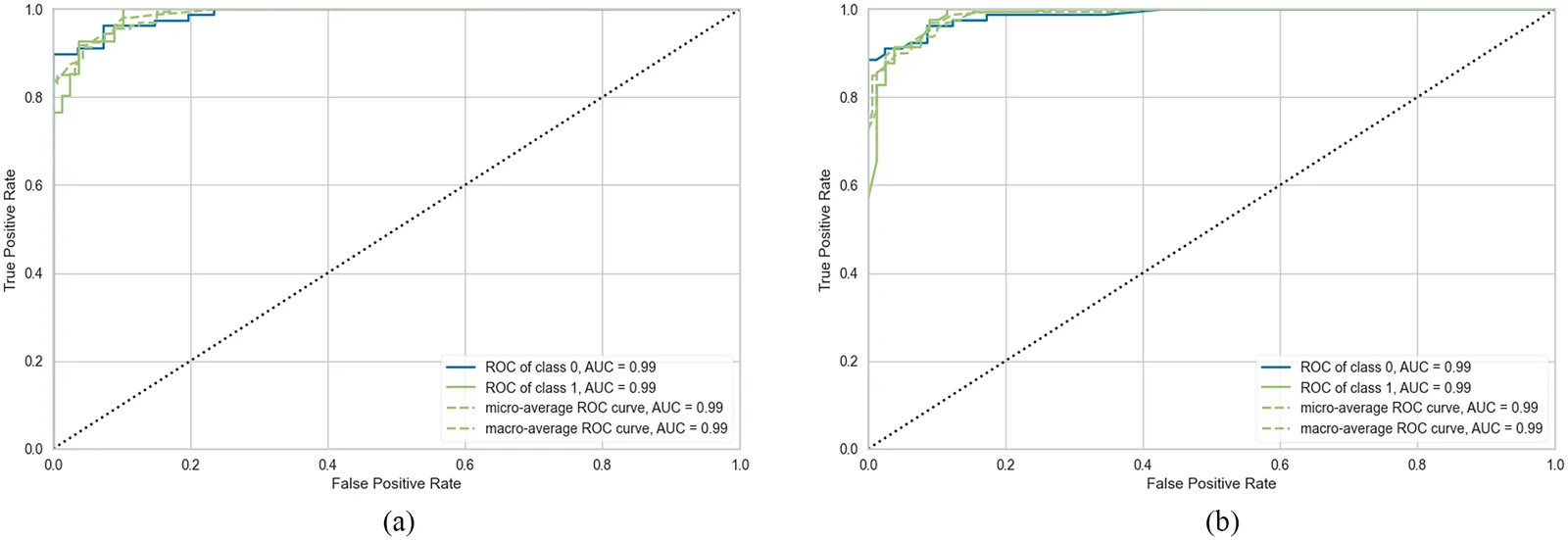
AUC-ROC curves for a) voting and b) stacking using selected models on balanced dataset.

**Fig 17:**
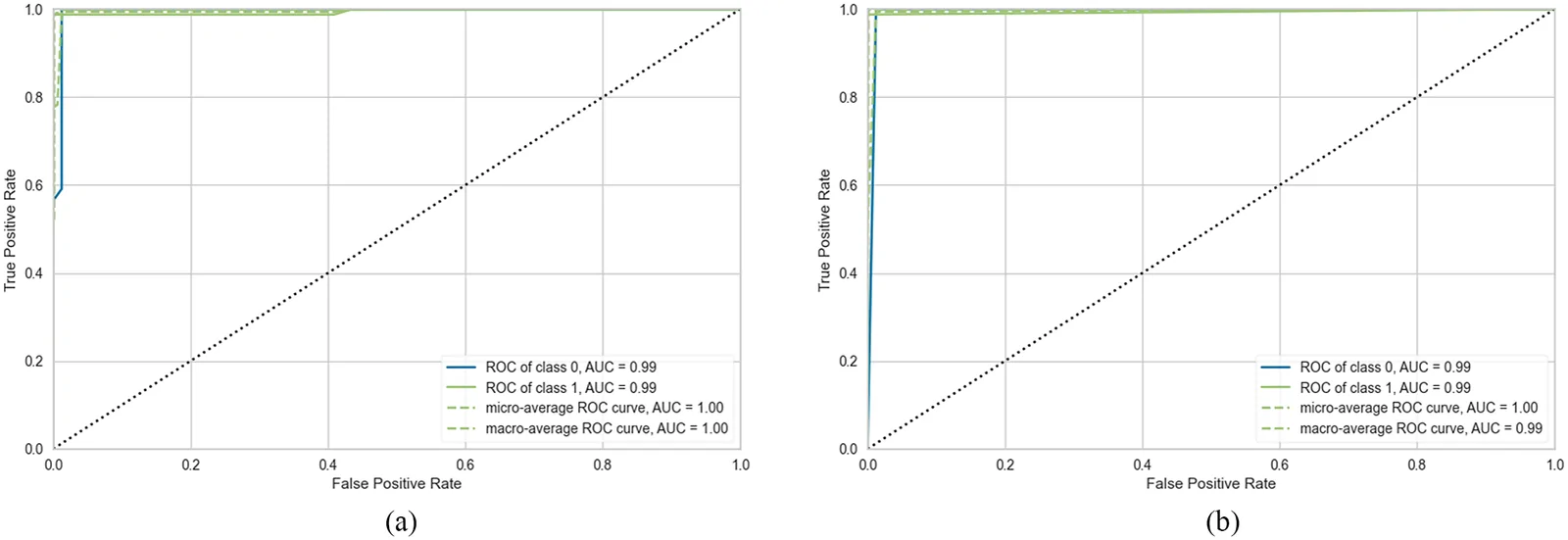
AUC-ROC curves for a) voting and b) stacking using selected models on upsampled dataset.

The AUC-ROC curves of the voting and stacking models for actual, balanced, and upsampled datasets are shown in Fig 15, Fig 16, and Fig 17, respectively. Overall, both stacking and voting models performed equally well across all datasets. However, the voting model slightly outperformed the stacking model in macro-average AUC-ROC on the upsampled dataset.

### 5.4 Result analysis

This section presents the performance of the proposed hybrid voting and stacking models and compares them against other models in this study and relevant state-of-the-art approaches in the literature.

#### 5.4.1 Comparing with other models.

This section compares the voting and stacking models from Phase III against models from Phases I and II. To ensure a focused comparison, the top three performing models for each metric were selected from Phase I, considering base learners, boosting, and bagging methods for each dataset. On the actual dataset, LR, LDA, and BDT showed the highest accuracies. On the balanced dataset, RF, CB, and GB performed best. For the upsampled dataset, DT, ET, and RF achieved the top accuracies.

**Fig 18:**
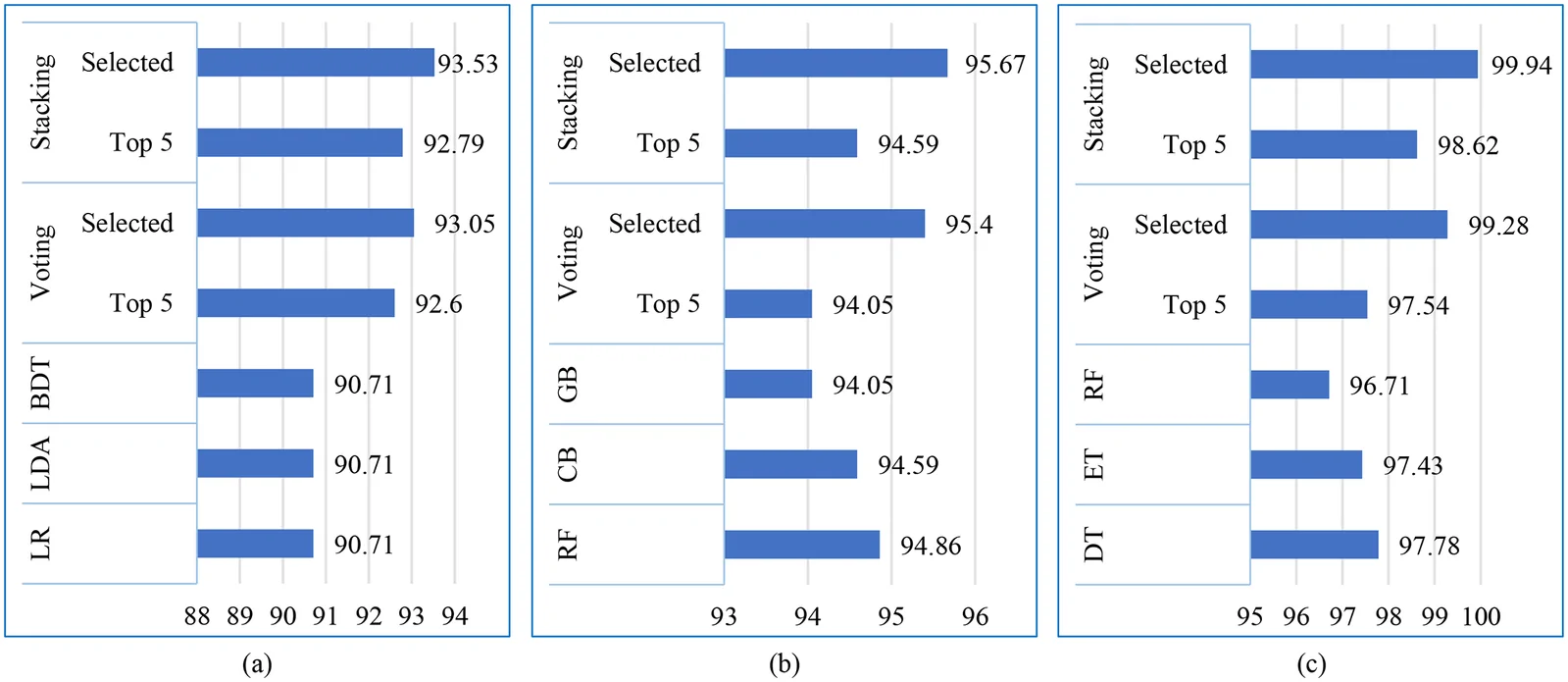
Accuracy comparison of the top three base models, voting and stacking using the top five models and voting and stacking using selected models for a) actual dataset, b) balanced dataset, and c) upsampled dataset.

**Fig 19:**
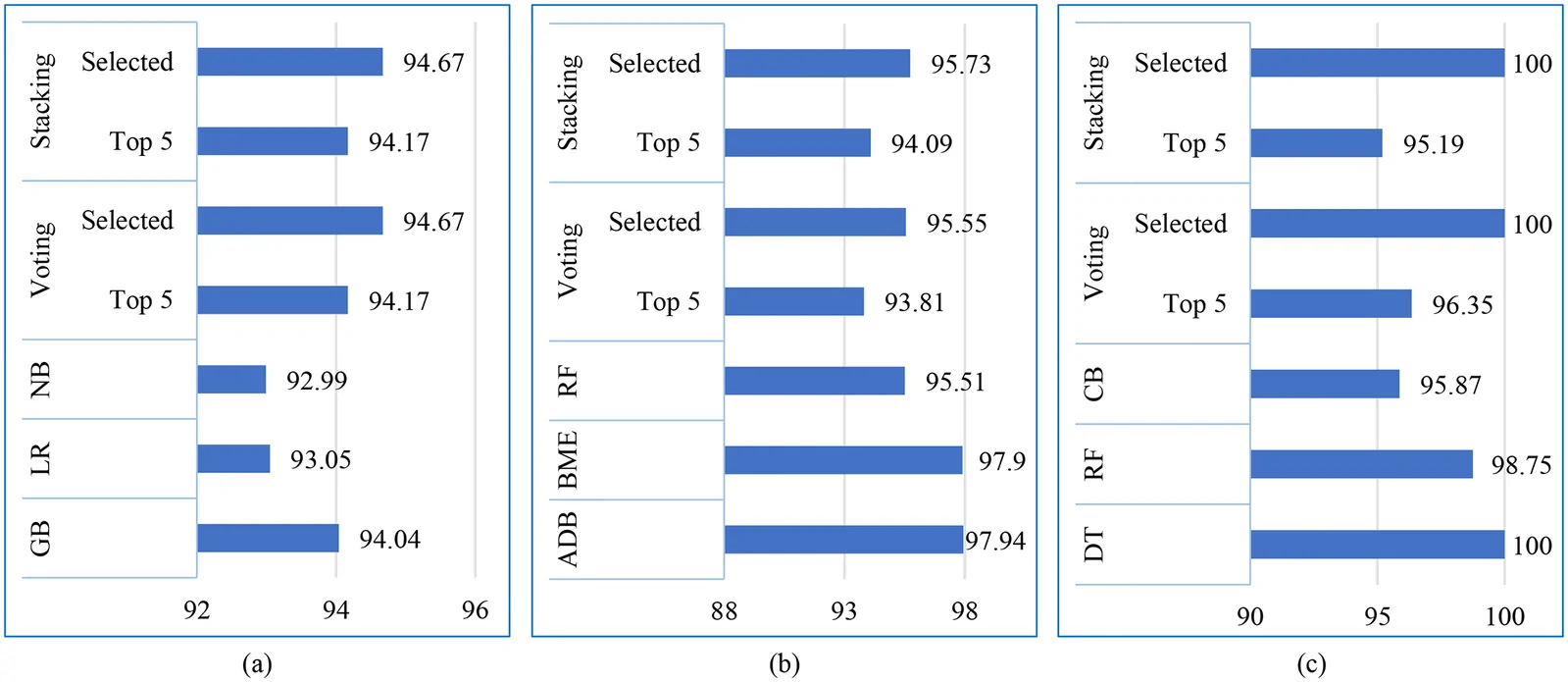
Precision comparison of the top three base models, voting and stacking using the top five models and voting and stacking using selected models for a) actual dataset, b) balanced dataset, and c) upsampled dataset.

**Fig 20:**
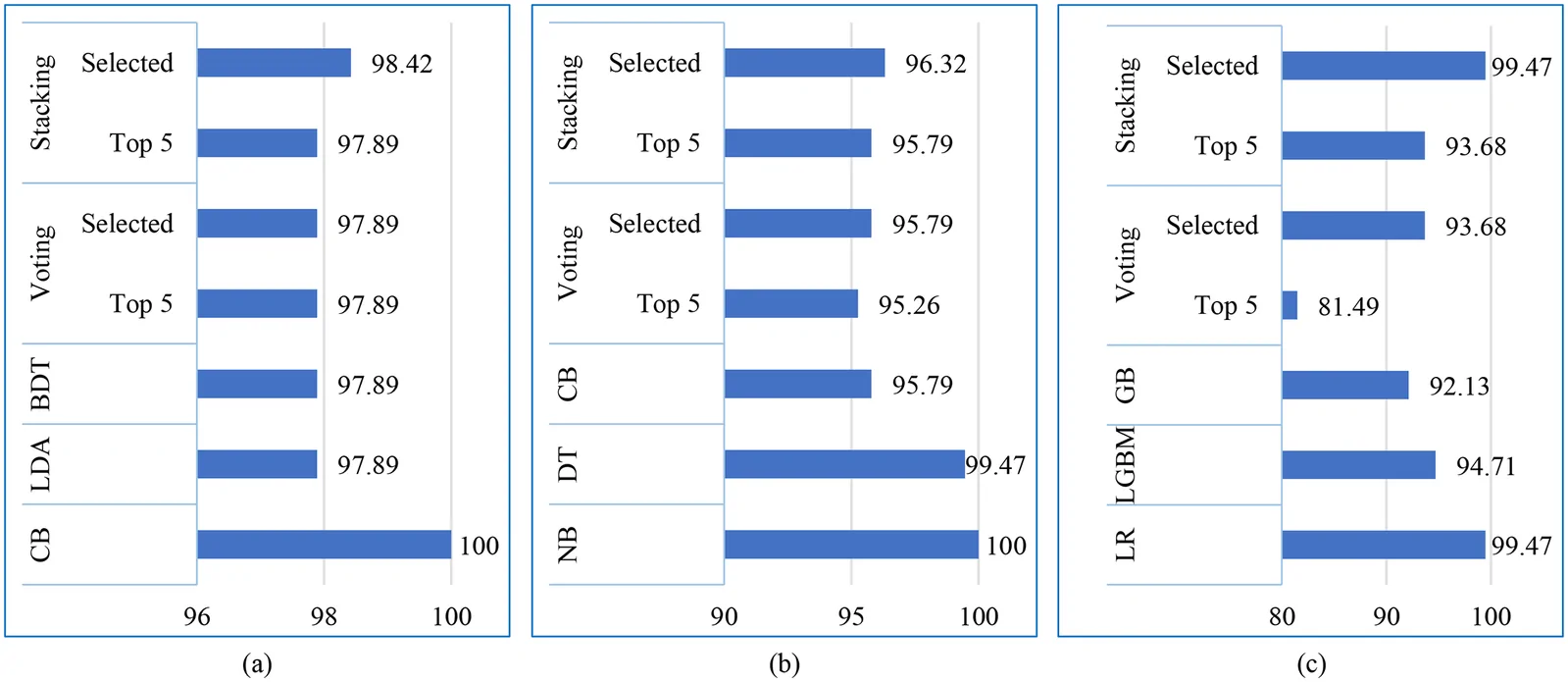
Recall comparison of the top three base models, voting and stacking using the top five models and voting and stacking using selected models for a) actual dataset, b) balanced dataset, and c) upsampled dataset.

**Fig 21:**
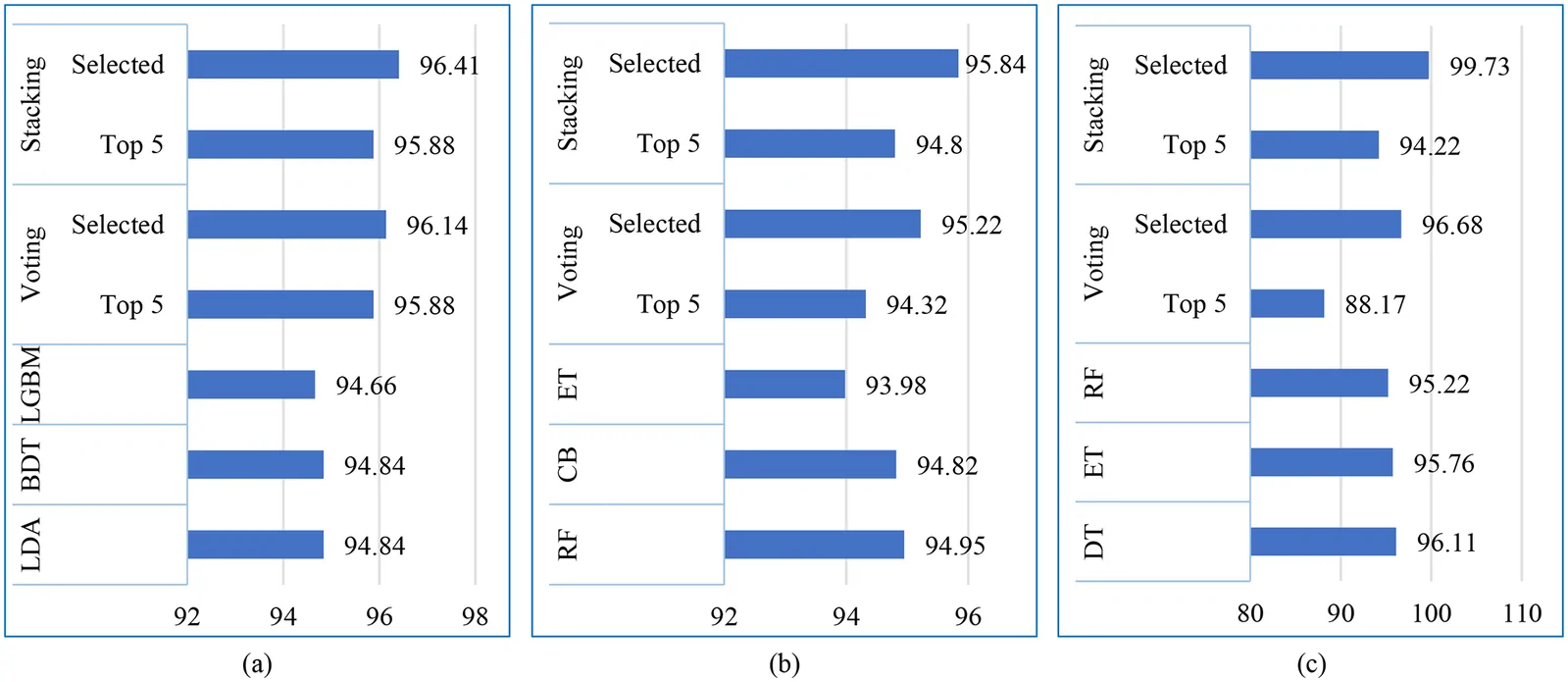
F1-score comparison of the top three base models, voting and stacking using the top five models and voting and stacking using selected models for a) actual dataset, b) balanced dataset, and c) upsampled dataset.

**Fig 22:**
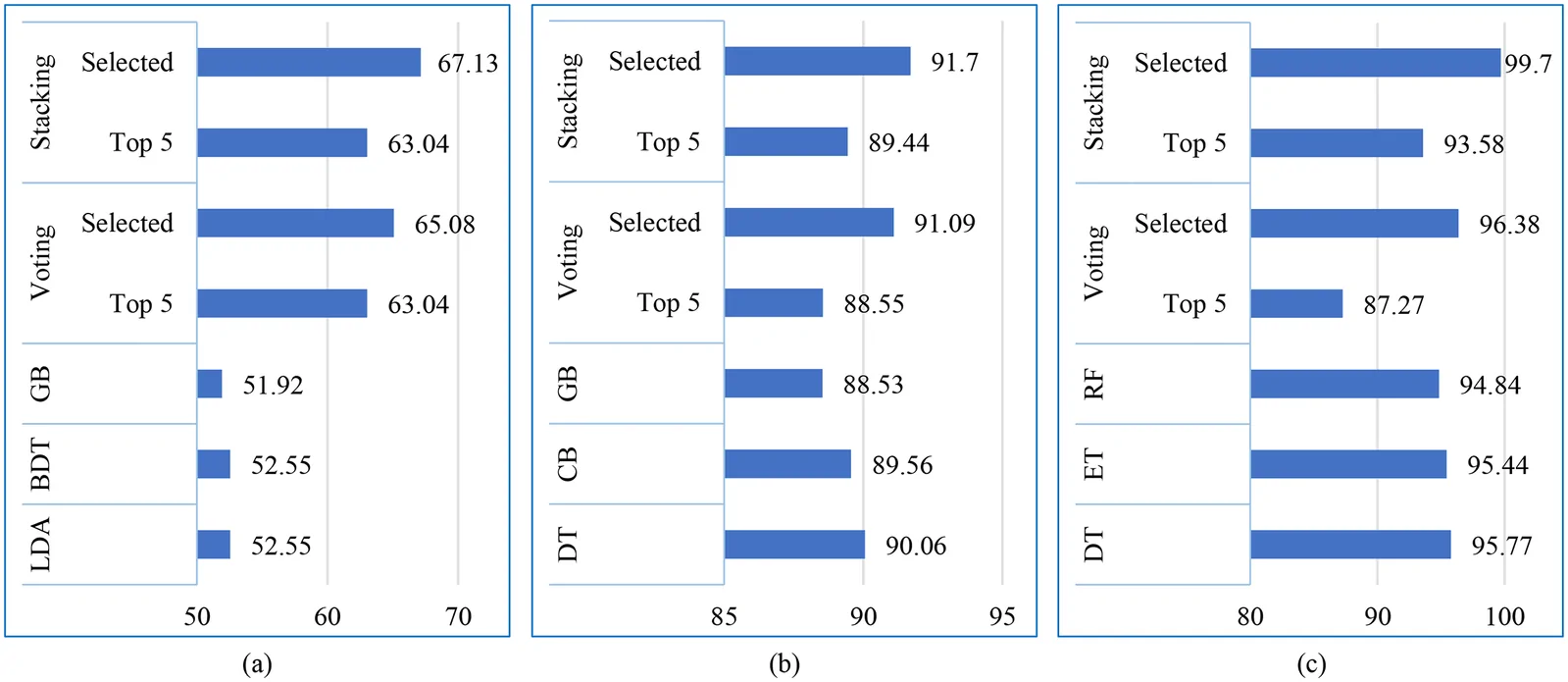
MCC comparison of the top three base models, voting and stacking using the top five models and voting and stacking using selected models for a) actual dataset, b) balanced dataset, and c) upsampled dataset.

**Fig 23:**
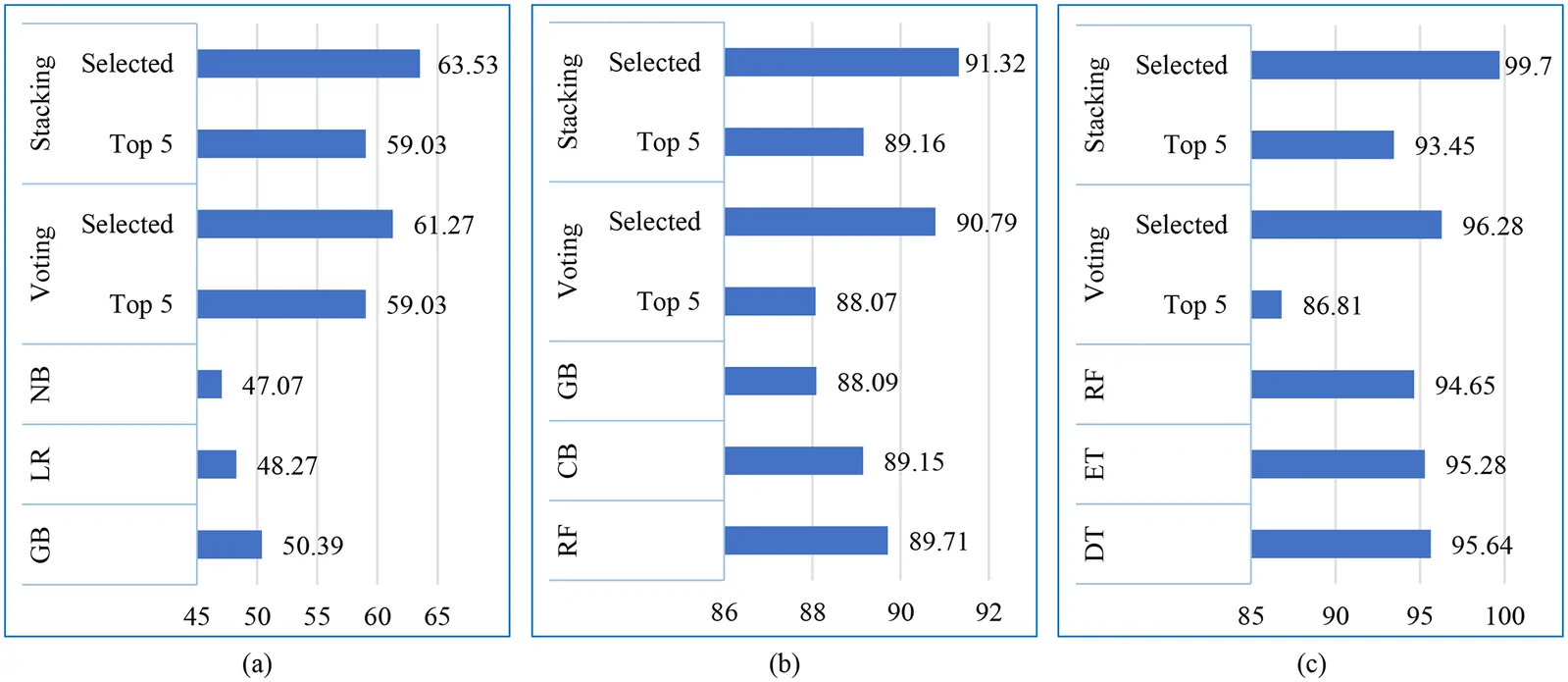
Kappa comparison of the top three base models, voting and stacking using the top five models and voting and stacking using selected models for a) actual dataset, b) balanced dataset, and c) upsampled dataset.

**Fig 24:**
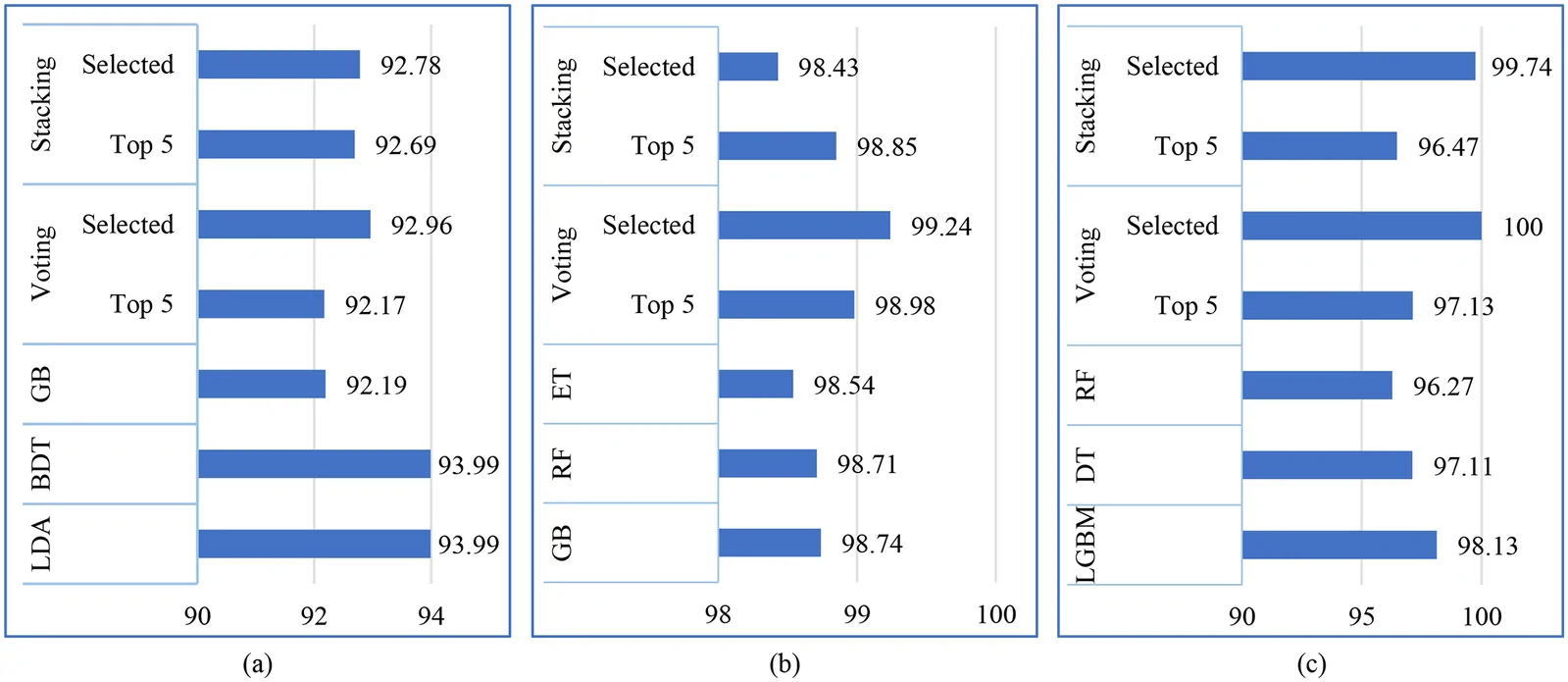
AUC of the top three base models, voting and stacking using top five models and voting and stacking using selected models for a) actual dataset, b) balanced dataset, and c) upsampled dataset.

The comparative performances across various metrics, accuracy, precision, recall, F1-score, MCC, kappa, and AUC, are shown in Fig 18, Fig 19, Fig 20, Fig 21, Fig 22, Fig 23, and Fig 24, respectively. The proposed voting and stacking models of Phase III outperformed all other models in every evaluation metric except recall. Recall that using stacking on the actual dataset is second-best (next to CB), whereas on the upsampled dataset it achieves a recall comparable to the best recall of LR and surpasses that of other models. Overall, the stacking model outperformed the voting model across all metrics and datasets.

**Fig 25:**
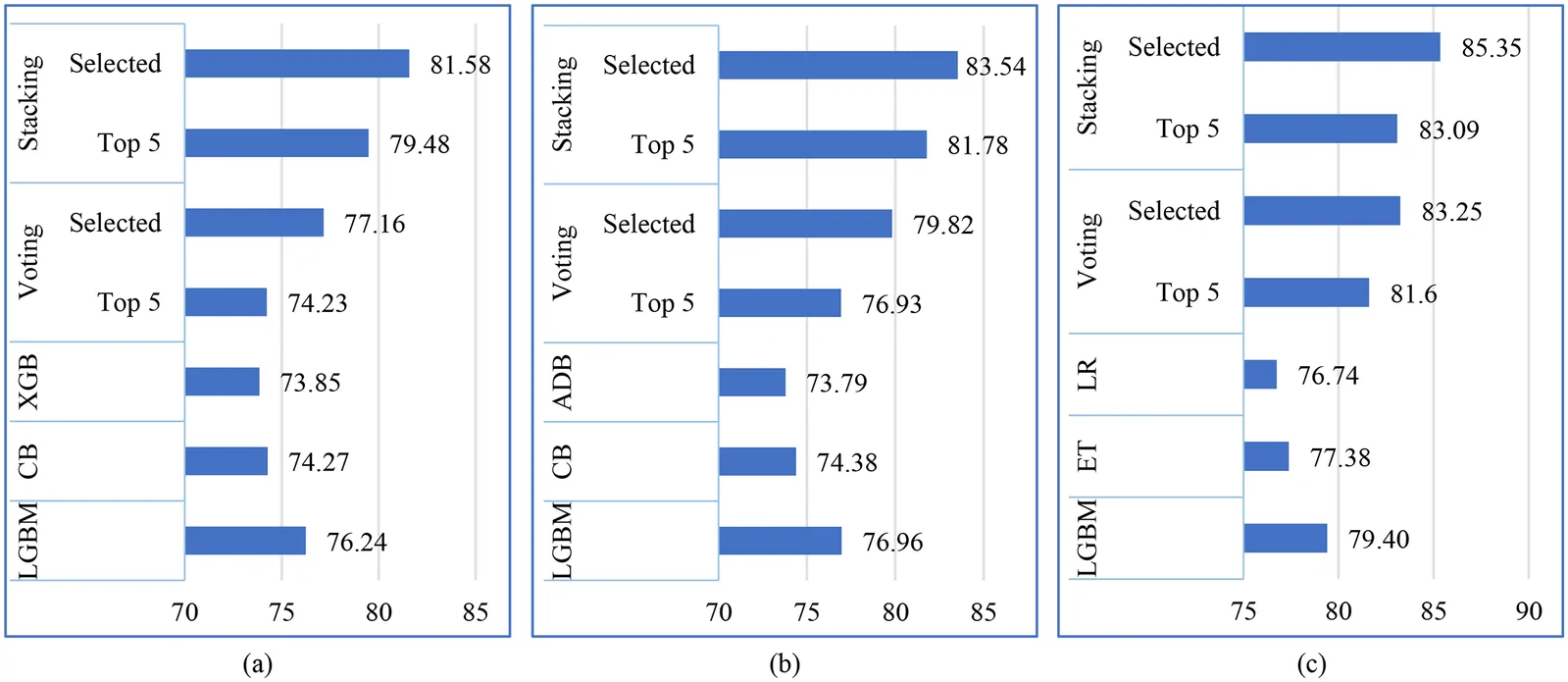
Composite performance scores comparison of the top three base models, voting and stacking using the top five models and voting and stacking using selected models for a) actual dataset, b) balanced dataset, and c) upsampled dataset.

Fig 25 presents a comparison of the composite performance scores (CCS) for the top three base models, as well as voting and stacking ensembles using both the top five and selected models across the three dataset variants. The CCS is defined as the average of multiple evaluation metrics (accuracy, precision, recall, F1-score, MCC, Kappa, and AUC) and is used here as a unified measure for comparative model assessment. The results show that ensemble models generally outperform individual models, with stacking consistently achieving higher CCS values than voting. LightGBM is a notable exception, outperforming the voting ensemble (top five) on both the actual and balanced datasets. The highest CCS is achieved by the stacking ensemble on the upsampled dataset, with scores of 83.09 and 85.35 for the top five and selected models, respectively. A clear trend is observed across datasets: CCS values increase from the original to the balanced and further to the upsampled dataset. This reflects improved model performance under reduced class imbalance and increased sample size. Tree-based models such as LightGBM, AdaBoost, and XGBoost consistently perform well, likely due to their ability to capture nonlinear feature interactions and handle class imbalance more effectively.

#### 5.4.2 Robustness analysis of SMOTE application strategy.

Because the balanced and upsampled datasets were generated via synthetic augmentation, an additional experiment was conducted to assess whether the stage at which SMOTE was applied affected model performance. Two settings were considered: (i) synthetic samples generated before data splitting and (ii) synthetic samples generated only from the training data after splitting. In the latter setting, the data were first partitioned into training and validation subsets, after which SMOTE was fitted and applied exclusively to the training subset using the same configuration adopted in the main experiments. The corresponding validation subset retained its original class distribution and contained no synthetic observations throughout model training and evaluation, thereby preventing information leakage during the resampling process. The corresponding results are reported in Table 9.

**Table 9:** Impact of SMOTE application strategy on ensemble model performance.

Dataset	Ensemble approach	Base learners	SMOTE application	Accuracy	Precision	Recall	F1-score	MCC	Kappa	AUC
Balanced	Stacking	Selected 5	Before splitting	93.78	94.14	94.21	93.98	87.94	87.54	98.54
			After splitting	95.67	95.20	96.84	95.87	91.65	91.32	99.02
		Top 5	Before splitting	92.97	93.89	92.63	93.12	86.20	85.92	96.46
			After splitting	94.59	97.11	93.68	94.68	89.59	89.17	98.42
	Voting	Selected 5	Before splitting	92.67	88.05	99.47	93.35	86.22	85.29	93.96
			After splitting	95.40	95.55	95.79	95.53	91.09	90.79	99.24
		Top 5	Before splitting	91.06	90.76	92.11	91.33	83.32	82.11	96.76
			After splitting	94.05	94.99	93.68	94.11	88.53	88.09	98.74
Upsampled	Stacking	Selected 5	Before splitting	97.43	95.76	80.96	87.59	86.64	86.17	95.65
			After splitting	99.94	100	99.47	99.73	99.70	99.70	99.74
		Top 5	Before splitting	94.86	95.51	94.74	94.95	90.06	89.71	98.71
			After splitting	98.62	95.19	93.68	94.22	93.58	93.45	96.47
	Voting	Selected 5	Before splitting	95.40	94.75	96.84	95.63	91.14	90.78	99.18
			After splitting	99.28	100	93.68	96.68	96.38	96.28	100
		Top 5	Before splitting	90.72	59.38	77.28	66.22	62.37	61.10	93.35
			After splitting	97.54	96.35	81.49	88.17	87.27	86.81	97.13

For the balanced dataset, applying SMOTE after splitting produced slightly higher performance across most evaluation metrics. The selected-5 stacking model, for instance, improved from 93.78% to 95.67% accuracy, while the F1-score increased from 93.98% to 95.87%. Similar improvements were observed for the voting ensembles.

The same comparison was performed on the upsampled dataset. Although the absolute values varied, the overall behaviour of the models remained unchanged. Stacking consistently outperformed voting, and the selected-5 stacking model continued to achieve the best results, recording 99.94% accuracy, 99.47% recall, and 99.74% AUC under the post-splitting SMOTE protocol.

An interesting observation is that the stricter resampling protocol did not reduce performance. In several cases, the results were marginally better. More importantly, the relative ranking of the ensemble models remained stable across both settings. This suggests that the advantage of the stacking framework is not dependent on a particular SMOTE implementation strategy. At the same time, the results should be interpreted within the broader limitations of synthetic augmentation and the relatively small size of the original dataset.

#### 5.4.3 Comparing with the state-of-the-art.

The effectiveness of the proposed ensemble model was validated through comparative evaluation against relevant research papers, using multiple performance metrics detailed in Table 10. We selected the stacking model for the upsampled dataset, given its superior performance over the voting model in our study. The comparative analysis includes studies that used ensemble learning methods to predict lung cancer from demographic data.

**Table 10:** Comparative analysis with similar research works.

Reference	Considered models	Dataset used	Sample size	No. of features	Splitting ratio (train:test)	Highest accuracy (%)	Precision (%)	Recall (%)	F1-score (%)	MCC (%)	Kappa	AUC (%)	XAI
Abdullah et al. [15]	SVM, KNN, and CNN	Lung cancer dataset UCI machine learning repository	32	56	–	95.56 (SVM)	95.60	95.60	95.40	–	–	95.50	x
Safiyari and Javidan [19]	Bagging, dagging, ADB, multi boosting and random subspace with a combination of several base classifiers	Surveillance, epidemiology, and end results (SEER) data released in April 2016	643924	149	–	88.98 (ADB)	–	–	–	–	–	94.90	x
Wang et al. [20]	Bagging, RF with BSPL (bootstrap with self-paced learning)	US National Library of Medicine National Institutes of Health	720	3	70:30	97.96 (RFSPL)	–	–	98.11	–	–	98.21	x
Siddhartha et al. [26]	Bagging-based ensemble algorithm using RF	Wroclaw Thoracic Surgery Centre for Patients (2007–2011)	470	17	90:10	84.04 (10-fold stratified cross-validation)	–	94.28	89.79	–	–	0.83	√
Ahmad and Mayya [18]	RF for lung cancer prediction tool (LCPT)	International cancer database and local medical questionnaire	1000	23	–	93.33 (RF)	81.82	100	90.09	93.33	84.28	100	x
Mamun et al. [21]	XGB, LGBM, bagging, ADB	Lung cancer dataset by staceyinrobert, Data World	309	16	70:30	94.42 (XGB)	95.66	94.46	–	–	–	98.14	x
Patra [16]	NB, KNN, and RBF	Lung cancer UCI machine learning repository	32	56	80:20	81.25% (RBF)	81.30	81.30	81.30	–	–	74.9%	x
Zhang et al. [27]	LR, NB, RF, and XGB	UK Biobank	467888	49	70:30	–	99.7 with XGB	98.1	98.9			99.8	√
Radhika et al. [17]	DT, SVM, NB, and LR	Lung cancer UCI machine learning repository (d1) and Data World (d2)	32 (d1) and 1000 (d2)	57 (d1) and 25 (d2)	7-fold cross-validation	99.20 (SVM)	89.99	99.01	99	89.36	–	99	x
Ganie and Pramanik [11]	NB, SVM, quadratic discriminant analysis, Ridge, KNN, LDA, LR, DT, RF, ET, GB, ADB, stacking, voting	Lung cancer dataset by staceyinrobert, Data World	309	16	70:30	99.22 (stacking)	93.16	100	96.4	99	95.96	96.07	√
		Lung cancer prediction (from Kaggle)	462000	24		99.75 (stacking)	99.75	99.77	99.75	100	99.62	99.63	
		Lung cancer (from Kaggle)	1298	9		99.81 (stacking)	100	99.62	99.8	100	99.62	99.62	
This paper	LR, NB, KNN, SVM, LDA, CB, GB, XGB, ADB, LGBM, DT, RF, ET, BDT, BME, stacking, voting	Lung cancer dataset by staceyinrobert, Data World	309	16	75:25	93.53 (stacking)	94.67	98.42	96.41	67.13	63.53	92.78	√
		Balanced version	528			95.67 (stacking)	95.73	96.32	95.84	91.70	91.32	98.43	
		Upsampled version	2387			99.94 (stacking)	100	99.47	99.73	99.70	99.70	99.74	

The results in Table 10 demonstrate the improvement our stacking approach achieves over existing methods. This advancement stems from the strategic selection of models and meta-learners, rigorous cross-validation, and comprehensive hyperparameter optimisation.

## 6 Statistical significance

To rigorously assess performance differences among the evaluated models, a non-parametric framework was employed using the Friedman aligned-rank test, followed by Holm-adjusted post hoc comparisons. The results indicate clear and statistically significant differences across models.

As shown in Table 11, the Friedman test yields highly significant results for both the voting and stacking with selected base models ensembles (p ≈ 0.000), leading to rejection of the null hypothesis. This confirms that the observed performance differences are statistically meaningful rather than due to random variation.

**Table 11:** Friedman aligned ranks test results for model comparison.

Ensemble approach	Statistic	p-value	Result
Voting (selected base models)	76.62267	0.00000	H0 is rejected
Stacking (selected base models)	76.63695	0.00000	H0 is rejected

The ranking results in Table 12 further substantiate this finding. Both voting and stacking with selected base models achieve the highest rank scores (105.86 and 107.86, respectively), clearly outperforming all baseline models. Among the individual learners, GB and RF consistently rank as the next-best-performing models. In contrast, simpler models such as SVM, KNN, and Naive Bayes occupy the lowest ranks, indicating comparatively weaker performance.

**Table 12:** Model rankings based on the Friedman aligned-ranks test for voting and stacking with selected base models.

*Voting with selected base models (VTS)*			*Stacking with selected base models (STS)*		
Rank	Rank score	Algorithm	Rank	Rank score	Algorithm
1	105.85714	VTS	1	107.85714	STS
2	88.14286	GB	2	87.14286	GB
3	88.00000	RF	3	87.00000	RF
4	74.28571	LGBM	4	73.57143	LGBM
5	68.42857	LR	5	67.57143	LR
6	67.28571	ET	6	67.42857	DT
7	66.85714	DT	7	67.42857	ET
8	59.28571	XGB	8	59.00000	XGB
9	56.07143	BDT	9	56.21429	BDT
10	56.07143	LDA	10	56.21429	LDA
11	54.28571	CB	11	54.57143	ADB
12	54.00000	ADB	12	54.57143	CB
13	28.71429	BME	13	28.85714	BME
14	15.57143	NB	14	15.57143	NB
15	15.14286	KNN	15	15.00000	KNN
16	6.00000	SVM	16	6.00000	SVM

The post hoc analysis provides detailed pairwise comparisons. As reported in Table 13, the voting (with selected models) model shows statistically significant improvements over several baseline methods, including SVM, KNN, NB, BME, AdaBoost, CatBoost, LDA, and BDT. However, no statistically significant differences are observed when compared with stronger models such as GB, RF, LGBM, LR, DT, and ET. In contrast, the STS model demonstrates broader statistical superiority; it achieves statistically significant improvements over a wider set of models, including XGBoost in addition to weaker learners. Similar to voting (with selected models), its differences from top-performing models such as GB and RF are not statistically significant, indicating comparable performance at the highest level.

**Table 13:** Post hoc pairwise comparison of voting and stacking with selected base models with baseline models (Holm-Adjusted).

*Voting with selected base models (VTS)*				*Stacking with selected base models (STS)*			
Comparison	Statistic	Adjusted p-value	Result	Comparison	Statistic	Adjusted p-value	Result
VTS vs SVM	5.75249	0.00000	H0 is rejected	STS vs SVM	5.86770	0.00000	H0 is rejected
VTS vs KNN	5.22579	0.00000	H0 is rejected	STS vs KNN	5.34924	0.00000	H0 is rejected
VTS vs NB	5.20110	0.00000	H0 is rejected	STS vs NB	5.31632	0.00000	H0 is rejected
VTS vs BME	4.44398	0.00011	H0 is rejected	STS vs BME	4.55097	0.00006	H0 is rejected
VTS vs ADB	2.98734	0.03096	H0 is rejected	STS vs CB	3.06964	0.02357	H0 is rejected
VTS vs CB	2.97088	0.03096	H0 is rejected	STS vs ADB	3.06964	0.02357	H0 is rejected
VTS vs LDA	2.86801	0.03718	H0 is rejected	STS vs LDA	2.97500	0.02637	H0 is rejected
VTS vs BDT	2.86801	0.03718	H0 is rejected	STS vs BDT	2.97500	0.02637	H0 is rejected
VTS vs XGB	2.68285	0.05110	H0 is accepted	STS vs XGB	2.81452	0.03420	H0 is rejected
VTS vs DT	2.24668	0.14796	H0 is accepted	STS vs ET	2.32897	0.11916	H0 is accepted
VTS vs ET	2.22199	0.14796	H0 is accepted	STS vs DT	2.32897	0.11916	H0 is accepted
VTS vs LR	2.15615	0.14796	H0 is accepted	STS vs LR	2.32075	0.11916	H0 is accepted
VTS vs LGBM	1.81874	0.20685	H0 is accepted	STS vs LGBM	1.97510	0.14477	H0 is accepted
VTS vs RF	1.02870	0.60724	H0 is accepted	STS vs RF	1.20152	0.45910	H0 is accepted
VTS vs GB	1.02047	0.60724	H0 is accepted	STS vs GB	1.19329	0.45910	H0 is accepted

Finally, the Wilcoxon test results in Table 14 confirm that the proposed ensemble approaches yield statistically significant improvements at the dataset level (p ≈ 0.0425), further supporting the robustness of the observed performance gains.

**Table 14:** Wilcoxon signed-rank test results for ensemble model comparison.

Dataset	Statistic	p-value	Result
Lung cancer	2	0.04252247799805163	H0 is rejected

## 7 Explainability of the stacking and voting models

XAI encompasses methodologies that enhance the transparency and comprehensibility of AI models, thereby rendering their decisions accessible to human experts [48]. This is particularly critical in domains such as healthcare, where trust and accountability are paramount [49]. XAI supports both global and local explanations, which are essential for ensuring the reliability and utility of models in healthcare contexts. Global explanations elucidate the primary factors influencing disease outcomes, thereby assisting clinicians and researchers, while local explanations clarify how these factors affect individual patients, effectively bridging the gap between research and clinical practice.

To augment the interpretability of the proposed stacking and voting models for predicting lung cancer on a global scale, we employed the SHAP method [50]. Grounded in Shapley values from cooperative game theory, SHAP provides a consistent framework for assessing each feature’s impact on individual predictions, making it a widely adopted tool for elucidating complex machine learning models [51]. Additionally, we utilized LIME for local or immediate feature interpretation [52], offering rapid, case-specific insights into model predictions, which is particularly beneficial for real-time decision-making.

### 7.1 Global explanation

Global explanations provide comprehensive AI model behavior insights across patient populations by identifying key features like age, genetic markers, and lab results influencing predictions. This analysis ensures alignment of medical knowledge, validates model decisions, and identifies inconsistencies that need refinement. Global explanations also help identify biases, ensure fairness across demographics, and support ethical compliance with standards such as the General Data Protection Regulation (GDPR), the Health Insurance Portability and Accountability Act (HIPAA), and Food and Drug Administration (FDA) regulations. This transparency fosters trust in AI-driven medical decisions. In our analysis, we used SHAP feature importance values to rank features by their influence on predictions, regardless of whether the impact is positive or negative.

**Fig 26:**
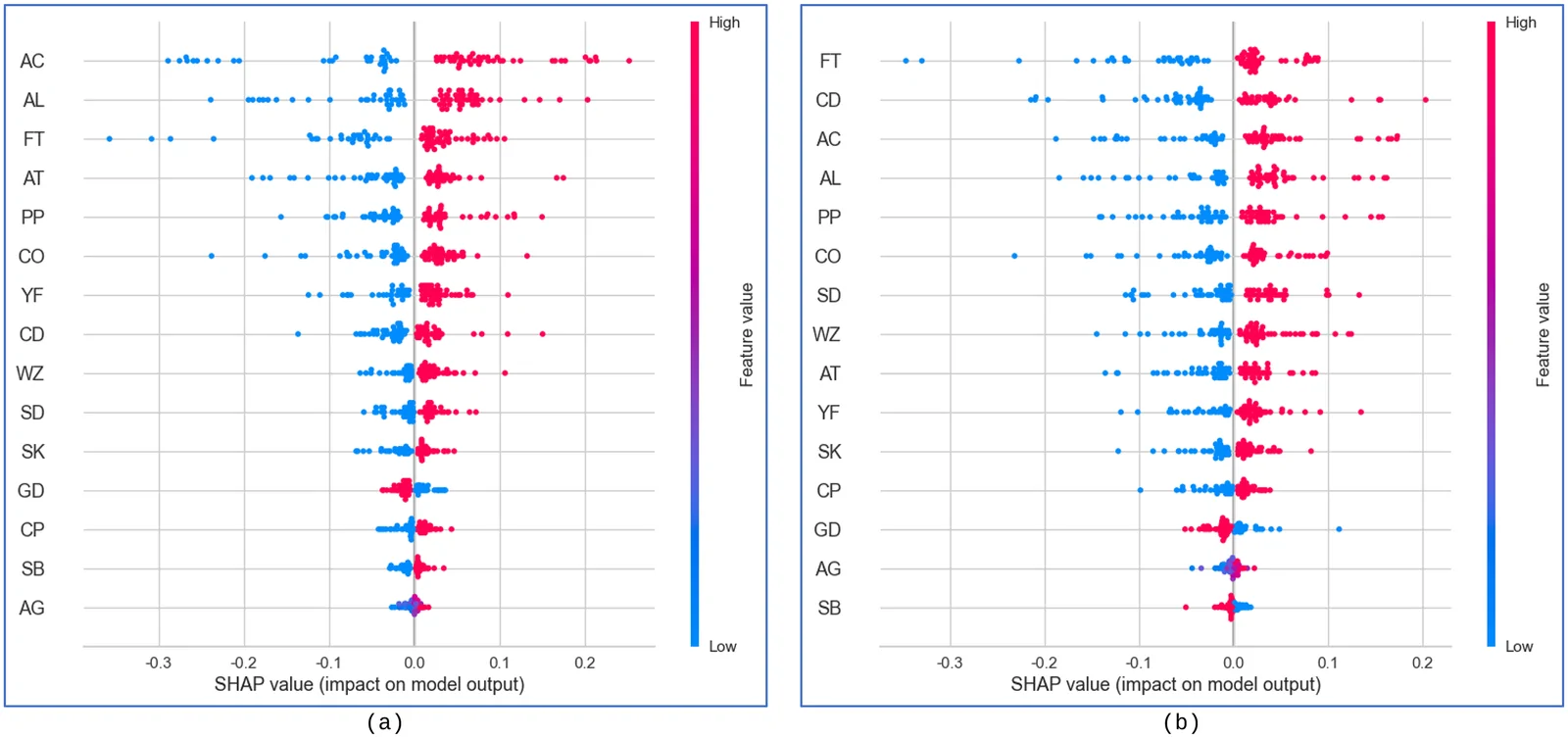
Beeswarm plot, ranked by mean absolute SHAP values for a) voting and b) stacking models on the actual dataset.

**Fig 27:**
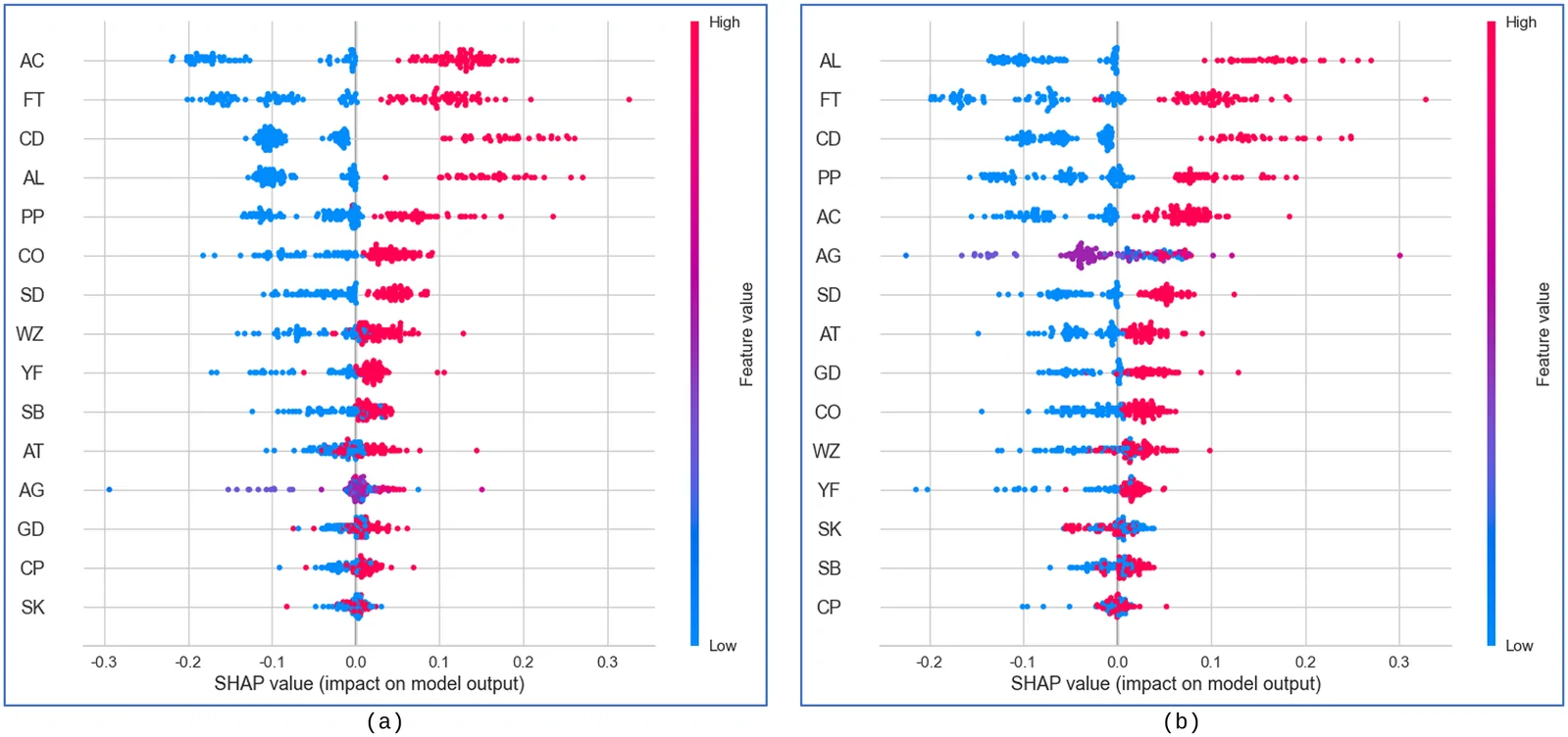
Beeswarm plot, ranked by mean absolute SHAP values for a) voting and b) stacking models on the balanced dataset.

**Fig 28:**
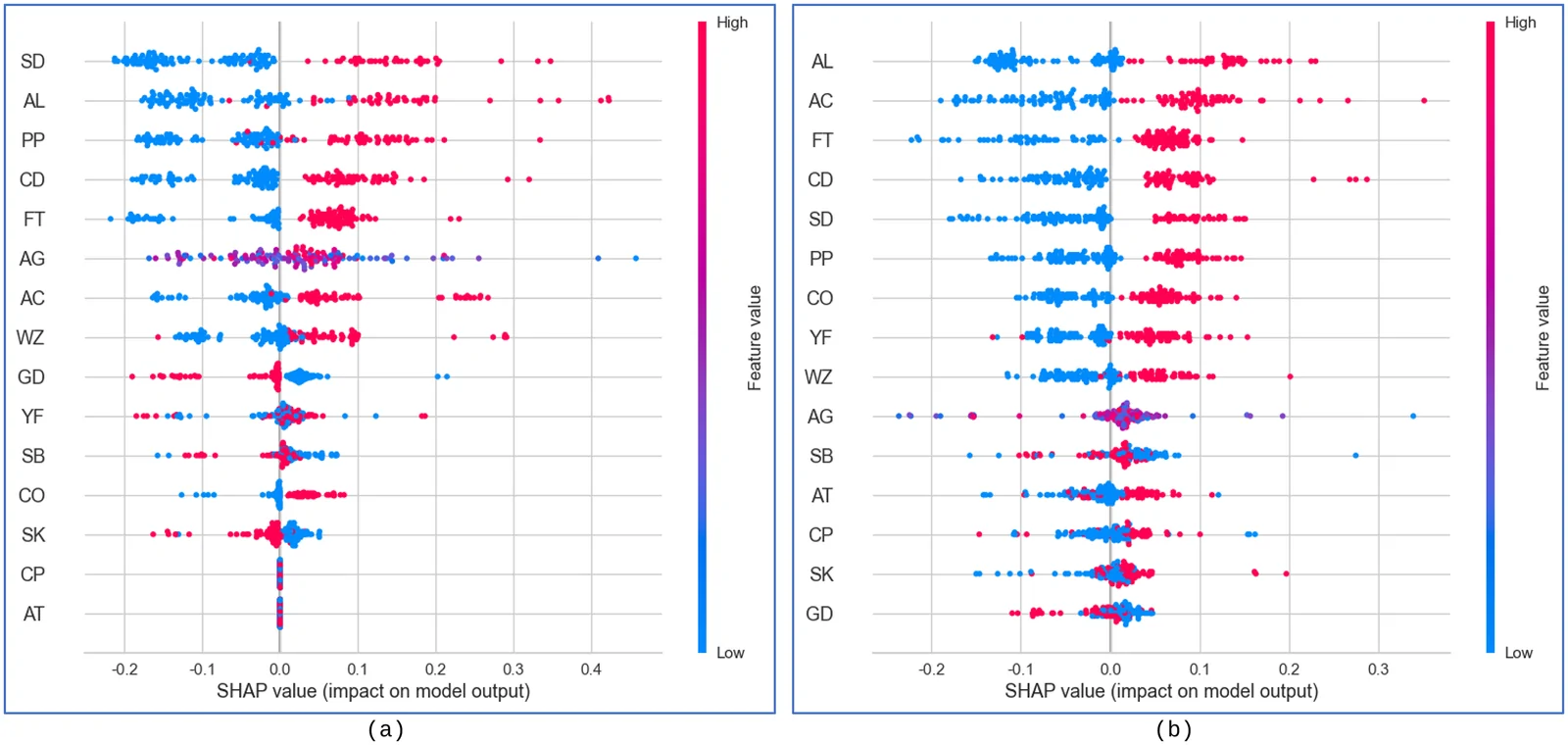
Beeswarm plot, ranked by mean absolute SHAP values for a) voting and b) stacking models on the upsampled dataset.

The global analyses of stacking and voting models across three datasets are depicted through Beeswarm plots, ranked by mean absolute SHAP values in Fig 26, Fig 27, and Fig 28, for actual, balanced, and upsampled datasets. Features are ranked from top to bottom by their impact on lung cancer prediction. The x-axis displays SHAP values, representing each feature's impact on the model's output. A positive SHAP value indicates contribution to predicting lung cancer, while a negative value suggests the prediction of lung cancer absence. Red dots represent high feature values, and blue dots represent low values, showing how feature magnitude affects prediction. Red dots indicate that higher feature values correlate with a higher probability of lung cancer. Blue dots indicate a lower probability of an association with lung cancer, as shown by negative SHAP values. For example, if a patient's feature F1 has a SHAP value of −0.2, it decreases the predicted probability of lung cancer by 0.2. Conversely, if F1's SHAP value is + 0.3, the model predicts a 0.3 increase, suggesting lung cancer.

In the stacking model, the features FT, CD, and AC exhibit the highest mean absolute SHAP values, indicating their dominant role in predictions. The broad dispersion of SHAP values for FT—spanning positive and negative impacts—suggests a context-dependent influence, where higher values (red dots) are strongly associated with increased cancer risk, while lower values (blue dots) decrease the prediction probability. Features AL and PP show significant but unidirectional effects, reinforcing their known associations with cancer prognosis. Features WZ, AT, YF, and SK exhibit mixed impacts (dots on both sides of zero), suggesting context-dependent effects. Features SB and GD cluster near zero, implying negligible contributions, reflecting either biological irrelevance or insufficient dataset representation. The voting model's plot shows similar top features (AC, AL, FT) and shares the same minimal-impact features (AG, SP, CP, GD) with stacking.

Similarly, using the balanced dataset, features such as AL, AC, FT, CD, and PP remain top contributors for both models. In contrast, features such as GD, SB, CP, and AG continue to have minimal impact on both models. Using the upsampled dataset, AL, AC, FT, CD, SD, and PP are the most influential predictors in both stack and voting. Features such as GD, SB, CP, and AG once again show very low or near-zero SHAP values, indicating minimal influence on predictions across both models.

### 7.2 Local explanation

Local explanations clarify individual model predictions, which are crucial in healthcare for making patient-specific decisions. By identifying key factors, such as biomarkers or medical history, these explanations enhance transparency, facilitate treatment planning, and foster clinician-patient trust. They also reveal errors by highlighting features that lead to incorrect outcomes, enabling model refinement. Additionally, they allow professionals to assess whether predictions align with established research, thereby enhancing transparency in AI diagnostics. This study utilises LIME plots to interpret stacking and voting models in lung cancer prediction. LIME generates localized explanations by approximating model behavior around instances, efficient for real-time use. Compared to SHAP, which provides precise but computationally intensive explanations of Shapley values, LIME offers quicker insights for exploratory analysis. Using LIME and SHAP together balances interpretability, depth, and computational efficiency in clinical decision-making.

**Fig 29:**
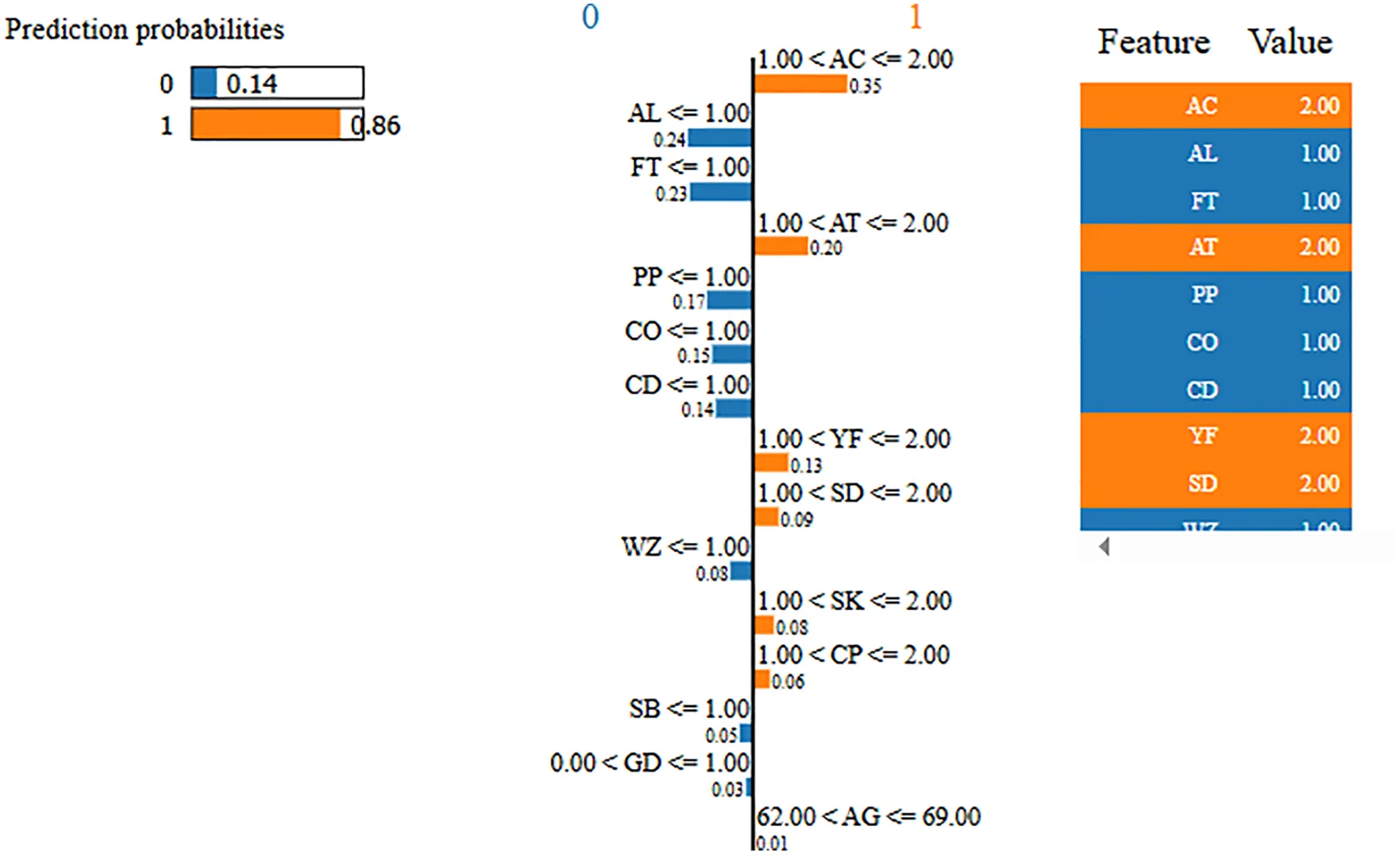
LIME plot for the voting model using the actual dataset.

**Fig 30:**
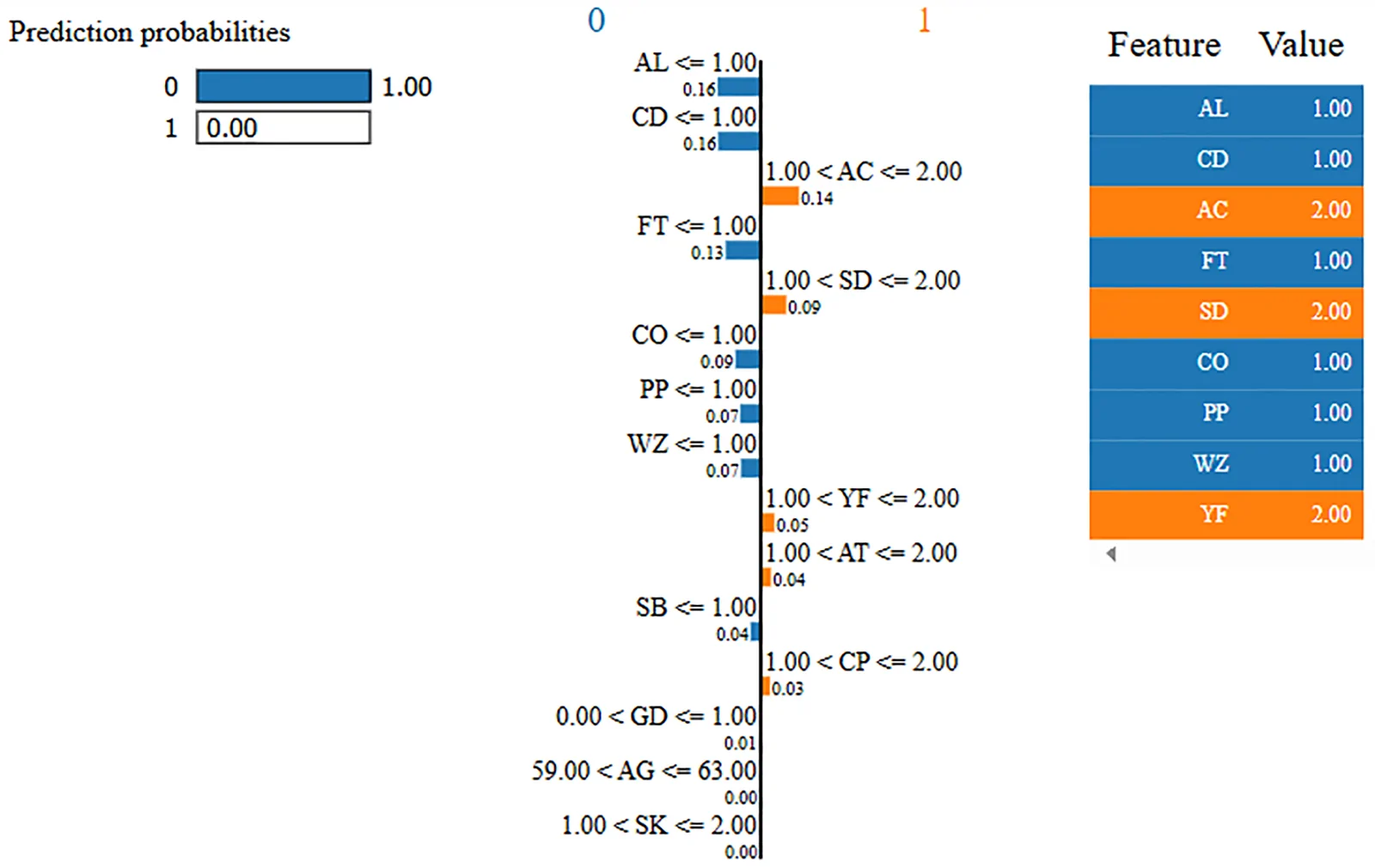
LIME plot for the voting model using the balanced dataset.

**Fig 31:**
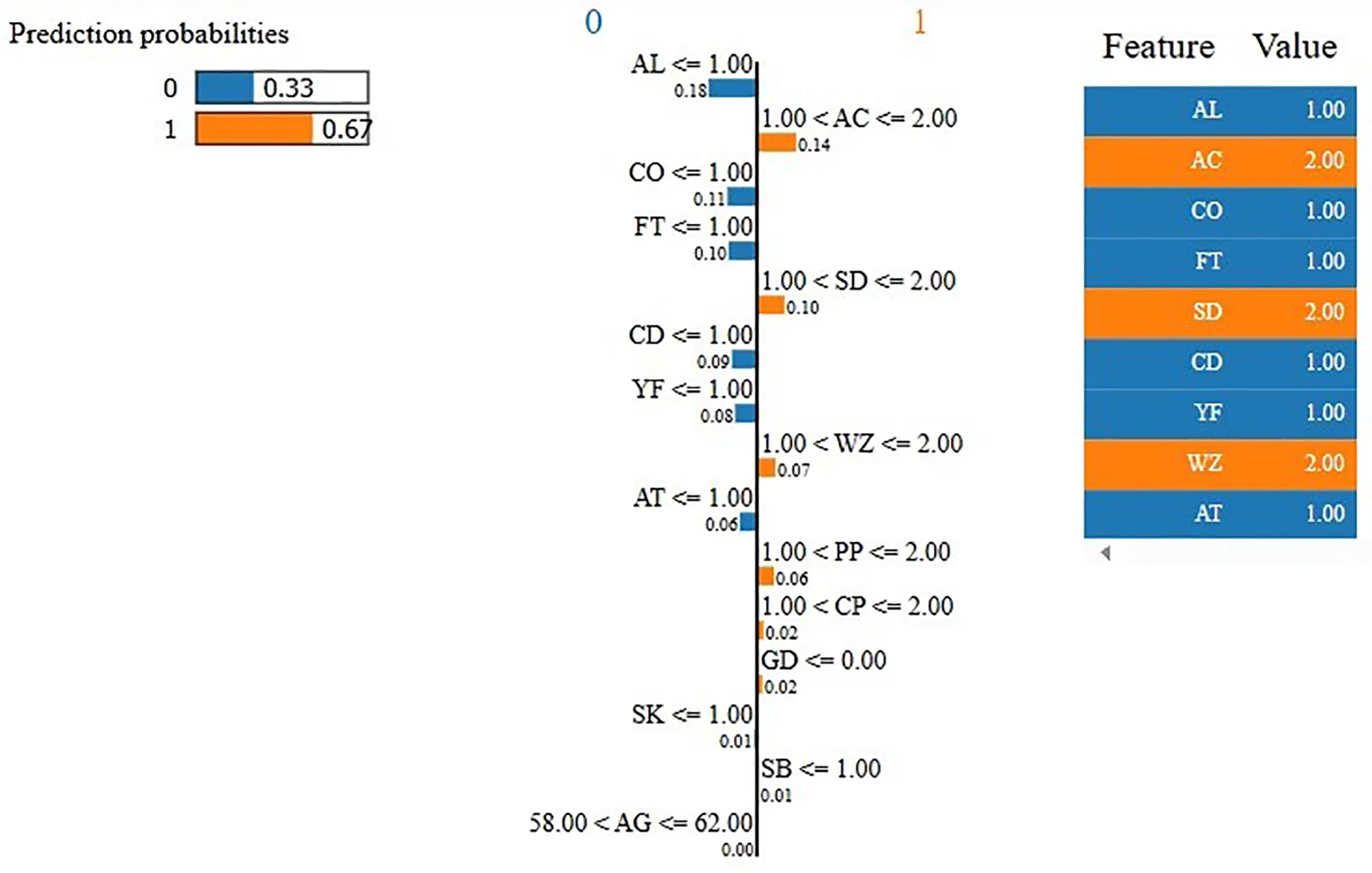
LIME plot for the voting model using the upsampled dataset.

**Fig 32:**
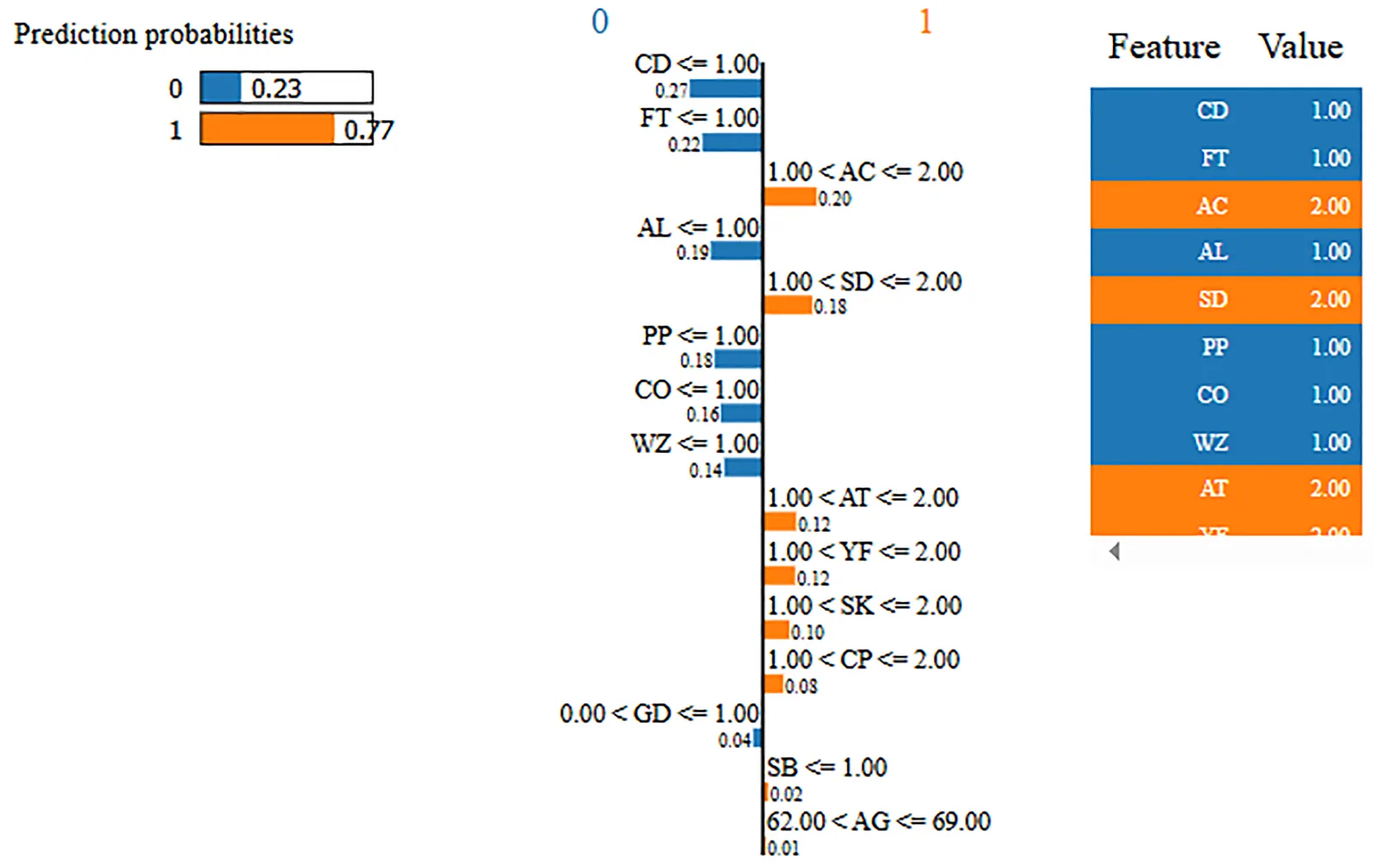
LIME plot for the stacking model using the actual dataset.

**Fig 33:**
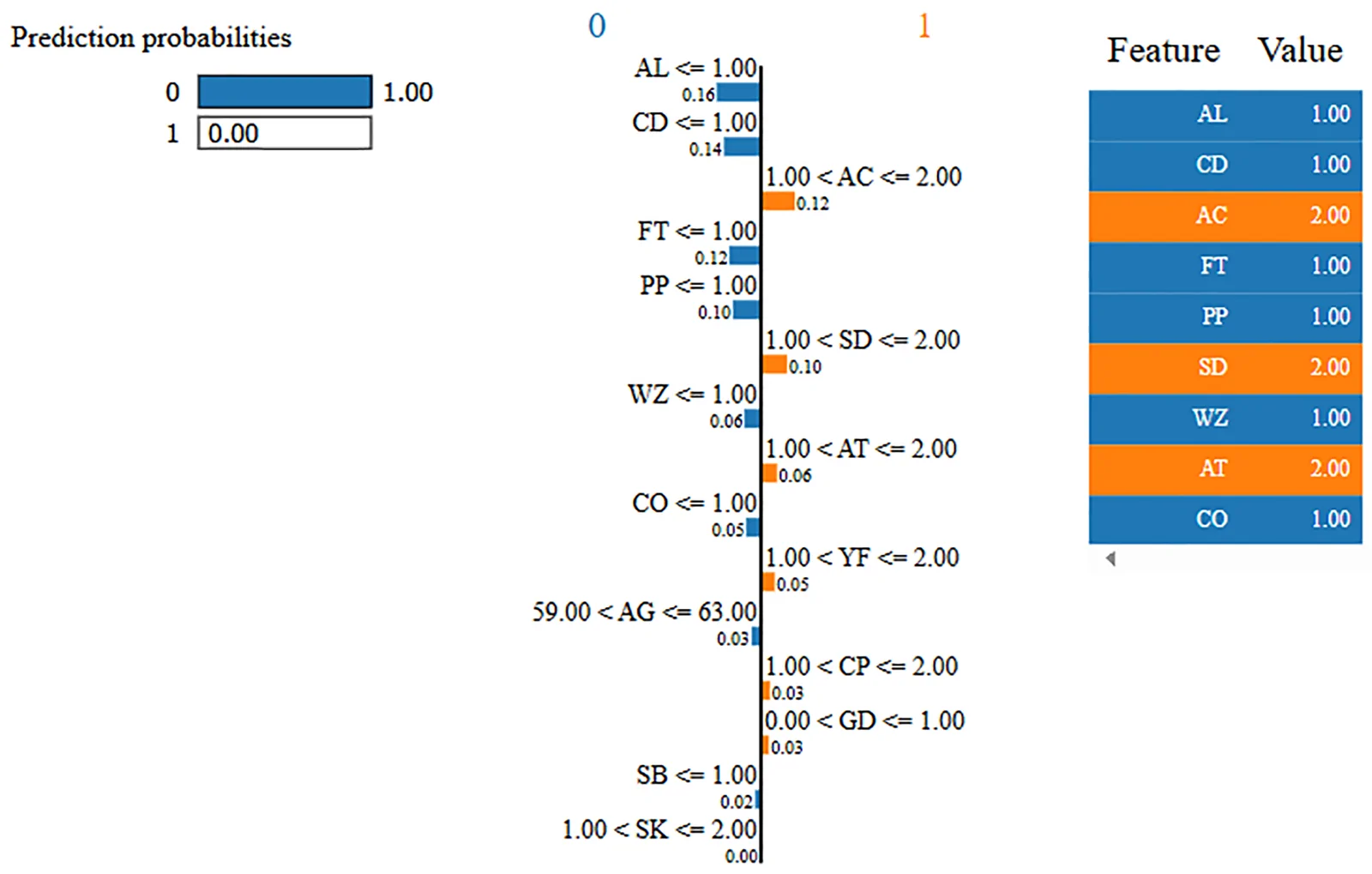
LIME plot for the stacking model using the balanced dataset.

**Fig 34:**
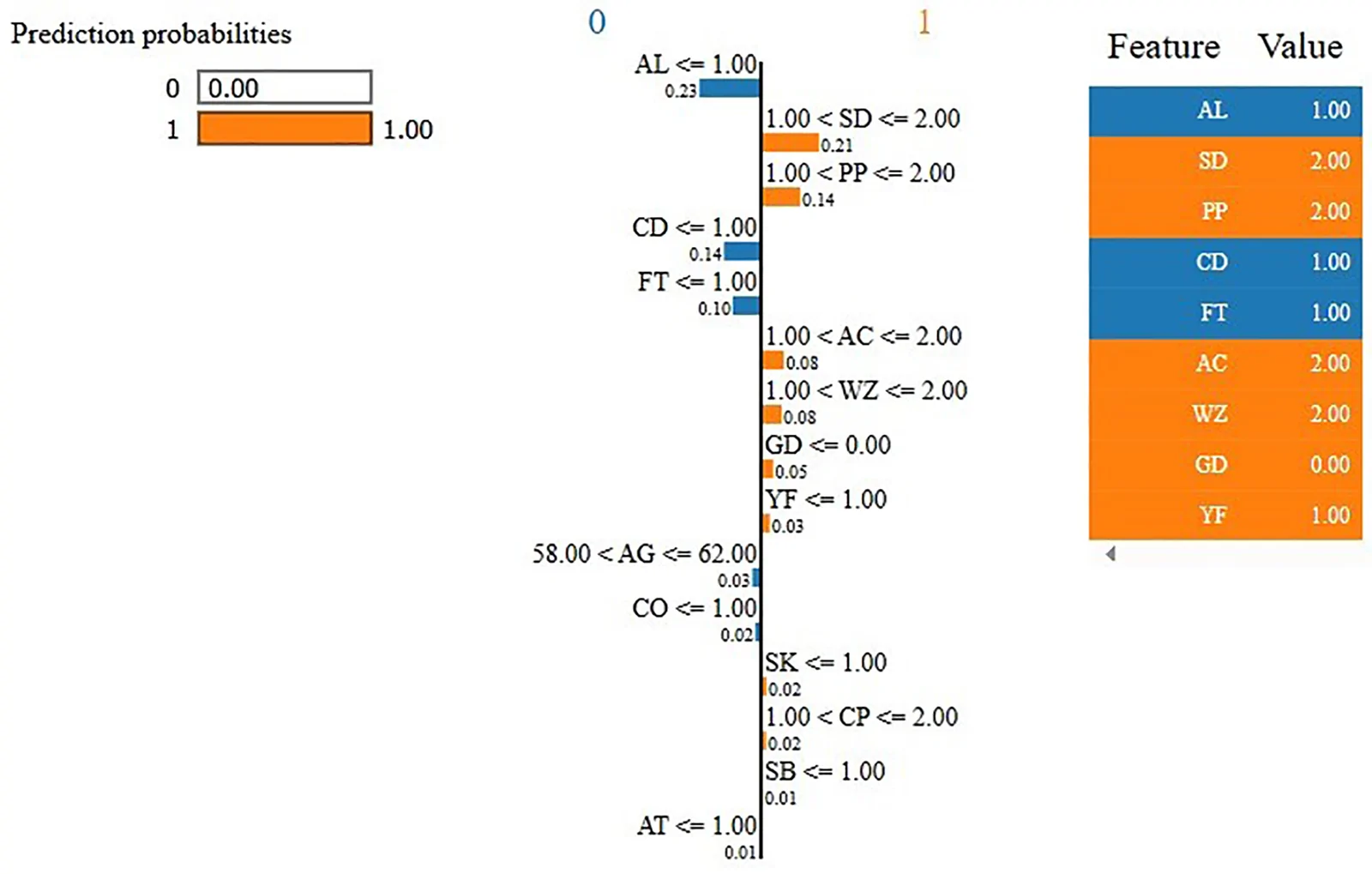
LIME plot for the stacking model using the upsampled dataset.

The LIME outputs of voting models are shown in Fig 29–30, and Fig 31 for actual, balanced, and upsampled datasets. The LIME outputs of stacking models are shown in Fig 32–33 and Fig 34 for actual, balanced, and upsampled datasets. They provide insights into feature contributions and prediction probabilities for specific instances. The visualisation illustrates the influence of features on model decisions. The left plot displays the prediction probabilities for lung cancer. The middle section presents feature contributions, while the right side lists features with actual values. Orange bars indicate features increasing lung cancer risk, while blue bars represent protective factors.

The voting model predicts 86% likelihood of cancer (class 1) and 14% likelihood of no cancer (class 0) based on the actual dataset (Fig 29). AC (+0.35) contributes most to lung cancer prediction, followed by AT (+0.20), YF (+0.13), SD +(0.09), SK (+0.08), CP (+0.06), and AG (+0.01). AL (−0.24) and FT (−0.23) exert greatest influence on predicting no lung cancer. PP (−0.17), CO (−0.15), and CD (−0.14) have a negative impact on lung cancer prediction. In the balanced dataset (Fig 30), AL (−0.16), CD (−0.16), and FT (+0.13) are the most significant features indicating the absence of lung cancer. AC (+0.14) is the strongest predictor for lung cancer, followed by SD (+0.9), YF (+0.05), AT (+0.04), and CP (+0.03). In the upsampled dataset (Fig 31), AL (−0.18) has the most significant negative impact, while AC (+0.14) has the most significant positive impact. AG (−0.00), SB (+0.01), and SK (−0.01) minimally influence prediction.

For the stacking model (Fig 32), CD (−0.27) primarily indicates the patient does not have lung cancer. FT (−0.22), AL (−0.19), PP (−0.18), CO (−0.16), and WZ (−0.14) are significant negative determinants. AC (+0.20) and SD (+0.18) are the strongest positive determinants for lung cancer. Other positive determinants include AT (+0.12), YF (+0.12), SK (+0.10), and CP (+0.08). A similar pattern appears in the balanced dataset (Fig 33). In the upsampled dataset (Fig 34), AL (−0.23) has the most significant negative impact, while SD (−0.21) has the most significant positive impact. AT (−0.01), SB (+0.01), CP (+0.02), SK (+0.02), and CO (0.02) have a minimal influence on the prediction.

## 8 Discussion and clinical implications

The development of interpretable ensemble models for lung cancer prediction using lifestyle and clinical indicators carries important clinical implications, particularly for early detection and risk stratification. The experimental results indicate that ensemble approaches—especially stacking and voting—consistently outperform individual base learners. While performance improved substantially on the balanced and upsampled datasets, these results should be interpreted with caution, as synthetic data augmentation may not fully capture the complexity and variability of real-world clinical populations. In this context, the stacking model's performance on the original dataset (93.53% accuracy) provides a more realistic estimate of its practical utility. The near-perfect accuracy observed on the upsampled dataset demonstrates the model's ability to exploit additional training information but should not be viewed as representative of expected clinical performance.

Although the stacking model achieves the highest overall performance across multiple evaluation metrics, its recall is marginally lower than that of some individual base models. This highlights an inherent trade-off in classification, where optimizing for overall performance may lead to slight reductions in sensitivity. In the context of lung cancer prediction, recall is particularly critical because false negatives can delay diagnosis and treatment. Therefore, while the stacking model provides balanced and robust performance, model selection in clinical settings may require prioritizing recall depending on the specific application.

To further examine model behaviour, learning curve analysis was conducted across different dataset configurations. On both the original and SMOTE-balanced datasets, the voting and stacking models exhibit closely aligned training and validation curves, indicating stable learning dynamics and good generalization. However, a divergence emerges in the fully upsampled dataset: the voting model shows a clear gap between training and validation performance, suggesting susceptibility to overfitting with synthetic data. In contrast, the stacking model maintains close alignment between the curves, demonstrating greater robustness and resistance to overfitting under the same conditions.

These observations are further supported by stratified cross-validation, which helps mitigate variance in performance estimates. Notably, the stacking model achieves consistently strong results across all dataset variants, indicating that its performance is not solely dependent on synthetic augmentation but reflects a more stable and generalizable learning capability.

The statistical results indicate that the stacking model achieves the most consistent and highest overall performance, with significant improvements over several baseline methods. However, its performance is statistically comparable to strong tree-based models such as GB and RF. From a clinical perspective, this distinction is important. The statistical superiority of stacking over weaker models supports its use to improve reliability, particularly by reducing variability across patient cases. This is valuable in screening or triage settings, where consistent identification of high-risk individuals is critical.

At the same time, the lack of statistically significant differences between stacking and top-performing tree-based models suggests that comparable predictive performance can be achieved with less complex models. In practical terms, this means that in settings where interpretability, ease of deployment, or computational efficiency are priorities, models such as RF or GB may serve as effective alternatives. Conversely, in scenarios where robustness across heterogeneous data is essential—such as multi-center screening programs or populations with varying clinical profiles—the stacking model offers an advantage by integrating diverse learning patterns. This can help maintain stable performance when data characteristics shift.

Through comprehensive SHAP and LIME analyses, we identified several clinically relevant predictors that both confirm and challenge the established understanding of lung cancer risk factors. A deep dive into the feature importance using SHAP reveals a fascinating alignment with, and a few surprising deviations from, established clinical knowledge. The consistent identification of fatigue (FT) and alcohol consumption (AC) as top predictors strongly supports the model's clinical validity. Fatigue is a well-documented constitutional symptom of malignancy, often linked to systemic inflammation and tumor metabolic activity [53]. However, fatigue is a nonspecific and prevalent symptom in many chronic conditions, suggesting that its predictive power is strongest when combined with other indicators, such as smoking history or age [54]. Similarly, chronic alcohol use is a known, dose-dependent risk factor that can promote carcinogenesis [55,56]. The model's ability to capture the dose-response relationship, in which higher values push SHAP values positive (increased risk), mirrors clinical observations and reinforces confidence in the model's learning process. However, epidemiological evidence on alcohol as an independent risk factor for lung cancer is mixed, with some studies suggesting a synergistic effect with smoking due to enhanced carcinogen exposure (e.g., acetaldehyde) [57,58]. While smoking remains the most established risk factor, our model attributes substantial predictive weight to alcohol intake. This finding warrants further investigation—whether the model captures true biological risk or confounding behavioural patterns (e.g., heavy drinkers being more likely to smoke). Prospective cohort studies could clarify this relationship before clinical integration. Furthermore, the model's emphasis on swallowing difficulty (SD) is of high clinical importance. Dysphagia is a significant “red flag” symptom, often indicating local tumor progression where the mass may be compressing the esophagus, suggesting a more advanced stage [59].

However, the analysis also highlights key points for discussion, particularly regarding features that either conflict with or add nuance to established risk hierarchies. One notable observation is the relatively low importance attributed to smoking (SK), despite its well-established role as the primary risk factor for lung cancer [60]. As shown in the feature importance analyses presented in Section 5.3.5, this pattern was consistently observed across both voting and stacking models and across all three dataset variants.

To further investigate this apparent discrepancy, we performed a multicollinearity analysis using the Variance Inflation Factor (VIF). For a predictor $X_{i}$, the VIF is computed as:


$$\begin{array}{c} {\text{VIF}}_{i}=\frac{\text{1}}{\text{1}-R_{i}^{\text{2}}} \end{array}$$ (3)

where $R_{i}^{\text{2}}$ denotes the coefficient of determination obtained by regressing $X_{i}$ on all remaining predictors. Larger VIF values indicate stronger multicollinearity and greater redundancy among correlated variables.

The results of VIF analysis are presented in Table 15. Smoking (VIF = 11.16) and several smoking-associated predictors, including yellow fingers (VIF = 19.08), chronic cough (VIF = 17.47), shortness of breath (VIF = 18.78), and alcohol consumption (VIF = 17.79), exhibited substantial multicollinearity. This suggests that the predictive signal associated with smoking is distributed across correlated behavioural and symptom-related variables. Consequently, attribution methods such as SHAP may allocate importance across these related predictors rather than concentrating it solely on smoking. Therefore, the relatively lower importance assigned to smoking should not be interpreted as contradicting established epidemiological evidence, but rather as a consequence of multicollinearity within the dataset.

**Table 15:** Multicollinearity assessment of predictor variables using VIF.

Variable	VIF
GD	3.15
AG	43.12
SK	11.16
YF	19.08
AT	19.97
PP	13.98
CD	10.77
FT	18.99
AL	13.13
WZ	14.31
AC	17.79
CO	17.47
SB	18.78
SD	15.61
CP	13.92

A similar pattern is observed for other clinically established factors. The relatively low importance assigned to age (AG) and chest pain (CP) is also counterintuitive, given that advanced age is a major non-modifiable risk factor for cancer [61], and chest pain is a commonly reported clinical symptom in lung cancer patients [62]. These observations indicate that the model may be capturing more immediate, discriminative signals—such as fatigue, chronic disease, and swallowing difficulty—over broader or less specific indicators. Additionally, dataset-specific characteristics, such as class distribution and representation bias (e.g., elderly patients with non-malignant respiratory conditions), may further influence these patterns.

Importantly, these observations highlight a fundamental distinction between predictive modeling and epidemiological causality. SHAP-based feature importance reflects the contribution of features to the model’s predictions based on the available data distribution, rather than their true causal role in disease development. As a result, features that are strongly correlated with the outcome in the dataset—or that capture downstream effects—may receive higher importance than primary risk factors. Therefore, deviations from established clinical hierarchies should be interpreted as insights into the model’s learned representations and dataset characteristics, rather than contradictions of medical knowledge.

Another intriguing high-impact feature is allergy (AL). The relationship between allergies and lung cancer is complex and not fully understood. Some research suggests that a hyper-vigilant immune system in allergic individuals might offer a protective effect against certain cancers, including lung cancer [63], although the relationship varies depending on the specific allergy and cancer type [64]. The model's consistent recognition of this feature suggests it has identified a strong predictive pattern in the data, warranting further clinical investigation.

Other clinically relevant features, such as chronic disease (CD), demonstrate notable predictive contributions, with biologically plausible mechanisms involving inflammatory pathways. It makes sense, as pre-existing conditions, particularly respiratory ones, are known to increase lung cancer risk [65]. The minimal influence of gender (GD) contradicts known epidemiological patterns [66,67], possibly reflecting changing smoking demographics or dataset limitations. These findings emphasise the importance of contextual interpretation when applying model results to clinical decision-making. Future iterations could improve these features by integrating more detailed genetic or biomarker data to increase precision.

The clinical implications of these findings could be significant. The models might improve risk stratification by identifying high-risk individuals beyond current smoking-based criteria, potentially broadening eligibility for low-dose CT screening. Including symptoms like fatigue and coughing may allow earlier detection than traditional methods, while highlighting modifiable risk factors, such as alcohol consumption, could guide preventive counselling.

The local, patient-specific explanations provided by LIME further enhance the model's clinical utility. By showing how a combination of factors, like high alcohol consumption and fatigue, contributes to an individual's high-risk score, the model provides actionable insights. This can empower physicians during patient consultations, enabling them to have more informed discussions about specific lifestyle modifications and the concrete factors that elevate the patient's personal risk. Ultimately, while the stacking model demonstrates powerful predictive capabilities, its true clinical promise is unlocked by its interpretability, which provides the crucial bridge between statistical predictions and meaningful, actionable clinical insights.

**Fig 35:**
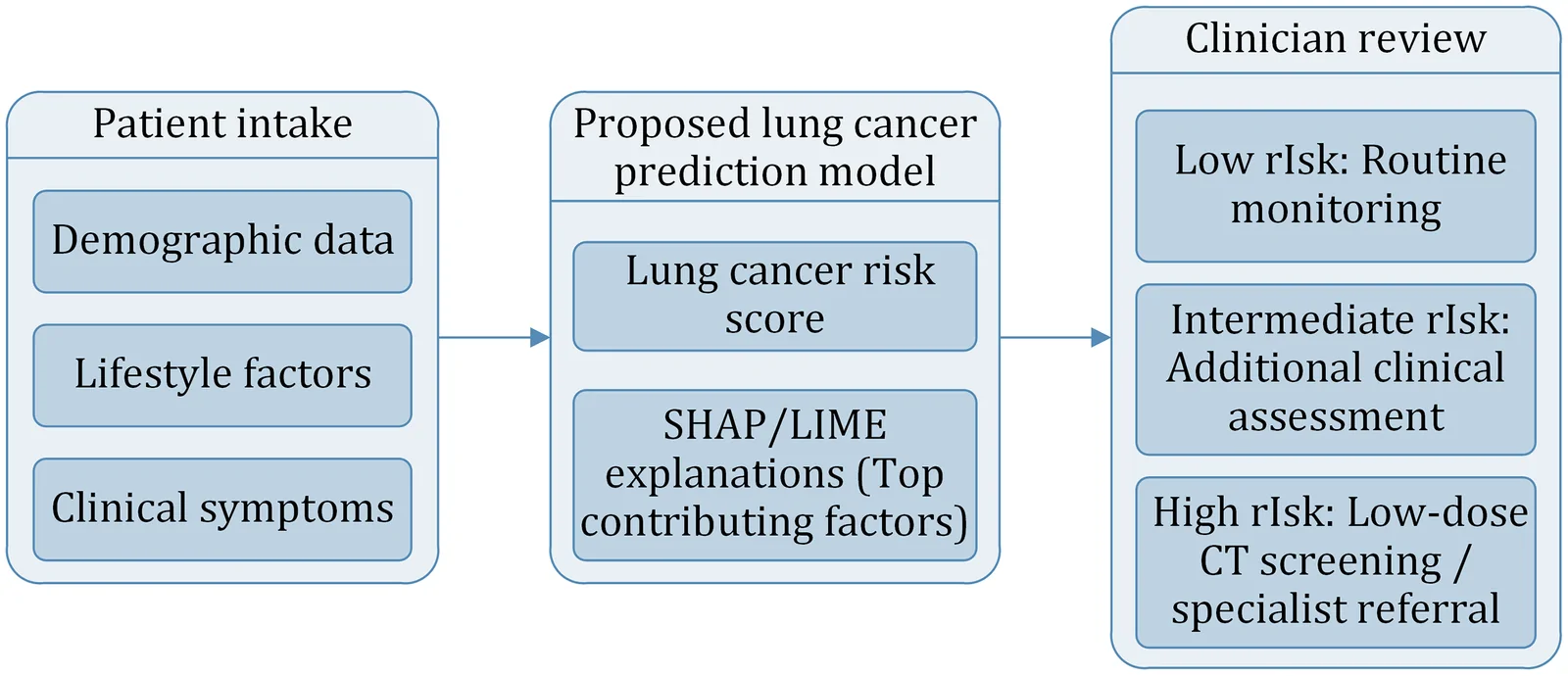
Conceptual decision-support workflow illustrating the potential integration of the proposed explainable lung cancer prediction framework into future clinical practice following appropriate validation.

Overall, from a clinical workflow perspective, the interpretability offered by SHAP and LIME can play a practical role in decision-making. As illustrated in Fig 35, patient demographic information, lifestyle factors, and clinical symptoms can be processed by the proposed stacking model to generate both a lung cancer risk score and SHAP/LIME-based explanations highlighting the most influential predictors. Global explanations can assist clinicians in identifying high-risk profiles based on combinations of lifestyle and clinical factors, potentially supporting early screening decisions such as prioritising patients for further diagnostic evaluation (e.g., low-dose CT scans). At the individual level, LIME-based explanations provide patient-specific insights, helping clinicians understand why a particular patient is flagged as high risk and guiding targeted follow-up investigations. The resulting risk score and explanatory factors can then be reviewed alongside conventional clinical findings to support risk-stratified decisions, including routine monitoring, additional clinical assessment, or specialist referral. Furthermore, the identification of modifiable contributors, such as alcohol consumption, may support personalised counselling and preventive interventions. Importantly, these explanations also allow clinicians to assess whether model predictions are clinically plausible, thereby reinforcing trust and enabling informed use of AI-assisted recommendations.

Overall, from a clinical workflow perspective, the interpretability offered by SHAP and LIME can play a practical role in decision-making. Fig 35 illustrates a conceptual decision-support workflow intended to demonstrate how the proposed framework could be integrated into a future clinical setting following appropriate validation. Patient demographic information, lifestyle factors, and clinical symptoms can be processed by the proposed stacking model to generate both a lung cancer risk score and SHAP/LIME-based explanations highlighting the most influential predictors. Global explanations can assist clinicians in identifying high-risk profiles based on combinations of lifestyle and clinical factors, potentially informing decisions regarding further diagnostic evaluation (e.g., consideration of low-dose CT screening). At the individual level, LIME-based explanations provide patient-specific insights, helping clinicians understand why a particular patient is flagged as high risk and guiding targeted follow-up investigations. The resulting risk score and explanatory factors can then be reviewed alongside conventional clinical findings to support risk stratification, including routine monitoring, additional clinical assessment, or specialist referral. Furthermore, the identification of modifiable contributors, such as alcohol consumption, may support personalised counselling and preventive interventions. Importantly, these explanations also allow clinicians to assess whether model predictions are clinically plausible, thereby reinforcing trust and enabling informed use of AI-assisted recommendations. The proposed workflow is intended solely as a conceptual illustration of how explainable AI could support clinical decision-making and should not be interpreted as a recommendation for clinical deployment or referral decisions based on the present study alone.

However, implementation challenges warrant careful consideration, including the subjective nature of key predictors such as fatigue and anxiety, which may necessitate the use of standardised assessment tools to ensure reliable clinical measurement. Although the unexpected deviations do not invalidate the prediction, they highlight an important implication: a clinician using this tool must interpret the output thoroughly. The model indicates the presence of a “symptom,” and the clinician must link it to the underlying “cause.” This emphasises that the tool is intended to support, not replace, clinical judgment.

From a deployment perspective, there is an inherent trade-off between predictive performance and computational complexity. The proposed stacking ensemble achieves the strongest predictive performance but requires greater computational resources than individual base learners due to its multi-stage architecture and meta-learning process. While this additional complexity is unlikely to pose a significant challenge in most clinical environments, where risk prediction is performed offline and does not require real-time response, it may become an important consideration in large-scale population screening systems or resource-constrained healthcare settings. Therefore, the choice of model should balance predictive accuracy with available computational resources and deployment requirements.

Improving feature measurement with more precise data on key variables, such as smoking (pack-years) and alcohol intake (standard drinks), could enhance predictive accuracy. Combining this with imaging results and molecular markers may lead to the development of multimodal prediction tools, while causal analysis can clarify whether the identified associations reflect genuine biological relationships. These developments will be essential for translating model outputs into actionable clinical insights.

## 9 Conclusions, limitations, and further scope

Lung cancer remains one of the deadliest diseases worldwide. Early prediction and detection are critical for timely medical intervention and improving patient outcomes. Machine learning techniques can be applied for this purpose. This study adopted ensemble learning to predict lung cancer. Four major ensemble approaches (boosting, bagging, voting, and stacking) were designed and evaluated for lung cancer prediction using a lifestyle dataset. The results showed that ensemble models outperformed individual base learners, with the stacking ensemble achieving the strongest overall performance. On the original dataset, the stacking model attained an accuracy of 93.53%, demonstrating its potential for lung cancer risk prediction using lifestyle and clinical indicators. Although substantially higher performance was observed on the balanced and upsampled datasets; however, these results should be interpreted in the context of synthetic data augmentation. Overall, the findings demonstrate that ensemble learning can effectively leverage complementary predictive patterns to improve lung cancer risk assessment and support early detection efforts. When interpreting models using XAI, according to the SHAP method, features such as allergy, alcohol consumption, fatigue, chronic disease, and peer pressure for smoking and alcohol most significantly influence lung cancer risk prediction, while gender, age, and shortness of breath have the least influence. The findings support the development of targeted interventions and risk management strategies to improve patient outcomes in lung cancer diagnosis and treatment.

This study has several limitations that warrant careful consideration. A primary limitation is the reliance on a single publicly available Kaggle dataset comprising 309 samples, which inherently restricts the generalizability of the findings. The model’s performance may therefore reflect characteristics that are specific to this dataset and its underlying demographic and clinical distribution rather than broadly representative patterns. The relatively small sample size further increases the risk of overfitting and learning dataset-specific relationships. Additionally, the absence of external validation limits the robustness of the results, as the proposed models were not evaluated on independent datasets collected from different institutions or patient populations. Consequently, the findings should be interpreted as preliminary, and the proposed framework should not be used to guide clinical decisions or low-dose CT referral until prospective and external validation has been completed.

Furthermore, the dataset may not capture the full spectrum of lifestyle and clinical risk factors associated with lung cancer. Real-world patient populations are highly heterogeneous, influenced by comorbidities, environmental exposures, genetic predispositions, and other factors that are not fully represented in the current data, potentially limiting the model's applicability in practical settings. The use of synthetic data augmentation introduces additional limitations. The strategy of upsampling both minority and majority classes to substantially enlarge the dataset is unconventional and may distort real-world class distributions, potentially leading to overly optimistic performance estimates. Accordingly, the results obtained on the balanced and, in particular, the upsampled datasets should be interpreted as comparative analyses under synthetic data augmentation rather than as estimates of expected real-world clinical performance. In addition, applying resampling prior to cross-validation may introduce bias in performance evaluation. Specifically, using SMOTE before cross-validation can cause synthetic samples derived from the same original instances to appear across training and validation folds, introducing information leakage and potentially inflating performance estimates.

To build upon this work and address these limitations, several enhancements can be explored. The most crucial next step is to evaluate the ensemble models on larger, more diverse, and externally validated datasets. This will involve sourcing data from multiple institutions to assess the generalizability and robustness of our approach across different populations and clinical settings. Such validation should also encompass a broader range of lifestyle and clinical characteristics to create a more comprehensive and accurate predictive model. Further validation experiments could explore the model's utility in multi-cancer studies. Assessing the performance of the proposed ensemble framework across other cancer types would help verify its generalizability beyond lung cancer and provide insights into its broader applicability in oncology. Methodologically, integrating our ensemble techniques with deep learning and advanced feature engineering may further augment predictive performance. Investigating novel hybrid architectures that synergistically combine these approaches represents a promising direction for enhancing predictive accuracy. Ultimately, the clinical value of this research lies in its practical application. Developing a user-friendly decision support tool that incorporates ensemble models could facilitate the practical application of lung cancer risk assessment and early detection in clinical settings. Translating these models into a practical tool with a physician-friendly interface is a critical step for successful clinical deployment, facilitating real-time risk assessment and supporting early detection strategies for lung cancer.

Additionally, implementing bias mitigation strategies—such as analysing underrepresented populations—will be crucial to improving model fairness and ensuring equitable healthcare outcomes. While SHAP elucidated feature significance, the study did not thoroughly explore the interpretability of ensemble models. Understanding the decision-making mechanisms of these models is essential for their implementation in healthcare, where trust and transparency are paramount. Future research could investigate model interpretability through various frameworks to clarify relationships between input data and predictions. Incorporating multi-modal data, such as imaging data with clinical indicators, could enhance the predictive capability and clinical relevance of models.

Future improvements involve broadening the scope to incorporate additional base learners and hybrid models that integrate ensemble methods with deep learning to enhance predictive accuracy. Examining demographic diversity within databases will be crucial to ensuring equitable healthcare outcomes. Integrating multiple data sources can help mitigate biases and improve the model's ability to identify individuals at risk. The inclusion of additional risk factors, such as genetic predispositions and environmental influences, in ensemble models warrants further investigation. Expanding the set of assessed characteristics could enhance their predictive power and relevance to personalised treatment.

To progress from research to clinical use, prospective validation across multiple centres is crucial. Including causal inference methods, such as counterfactual analysis, can help determine whether significant factors, such as alcohol consumption, are causally related or merely correlated. Additionally, combining imaging and biomarker data could improve predictive accuracy, leading to a multi-modal AI diagnostic system. Lastly, creating an interpretable decision-support tool using ensemble models could enhance real-world usefulness in healthcare. This technology would assist healthcare professionals in assessing risk and early detection of lung cancer, ultimately enhancing patient outcomes and healthcare services.
