# Supplementary material for: Lung cancer risk prediction using interpretable ensemble models on lifestyle and clinical data
Source: PLoS One. 2026 Sep 3;21(9):e0357291. doi: 10.1371/journal.pone.0357291 (PMC13541198; doi:10.1371/journal.pone.0357291)
Supplement: Supplementary file 1 [file pone.0357291.s002.docx]

**Manuscript ID: PONE-D-25-51253**

**Title: Lung cancer risk prediction using interpretable ensemble models on lifestyle and clinical data**

We thank the editor and two reviewers for the valuable and constructive comments on our work. We tried our best to address these points during the revision. The major changes are highlighted in red text in the revised manuscript. Below, we summarize our point-to-point response.

**Editorial comments:**

When submitting your revision, we need you to address these additional requirements.

1. Please ensure that your manuscript meets PLOS ONE's style requirements, including those for file naming. The PLOS ONE style templates can be found at

https://journals.plos.org/plosone/s/file?id=wjVg/PLOSOne_formatting_sample_main_body.pdf and

[https://journals.plos.org/plosone/s/file?id=ba62/PLOSOne_formatting_sample_title_authors_affiliations.pdf](https://track.editorialmanager.com/CL0/https:%2F%2Fjournals.plos.org%2Fplosone%2Fs%2Ffile%3Fid=ba62%2FPLOSOne_formatting_sample_title_authors_affiliations.pdf/1/010f019d260e1bd0-7e7c4d37-f4e0-493d-a7b0-e33ea24ac77f-000000/CDo1jwqW02fRDxS5s0PJaA3O7ggemNJBiLbKbW1J30I=256)

**Response:** We have revised the manuscript to comply with all PLOS ONE formatting and style requirements, including file structure and naming conventions, as per the provided templates.

2. Please note that PLOS One has specific guidelines on code sharing for submissions in which author-generated code underpins the findings in the manuscript. In these cases, we expect all author-generated code to be made available without restrictions upon publication of the work. Please review our guidelines at https://journals.plos.org/plosone/s/materials-and-software-sharing#loc-sharing-code and ensure that your code is shared in a way that follows best practice and facilitates reproducibility and reuse.

**Response:** We have created a publicly accessible GitHub repository containing all author-generated code used in this study. The repository link has been included in the Code Availability section of the manuscript to ensure reproducibility and reuse.

3. We note that the grant information you provided in the ‘Funding Information’ and ‘Financial Disclosure’ sections do not match.

When you resubmit, please ensure that you provide the correct grant numbers for the awards you received for your study in the ‘Funding Information’ section.

**Response:** We have carefully reviewed and corrected the Funding Information and Financial Disclosure sections to ensure consistency. The updated funding details now accurately reflect the support received for this study.

4. Thank you for stating in your Funding Statement:

ZZ is partially funded by his startup at The University of Texas Health Science Center at Houston, Houston, Texas, USA.

Please provide an amended statement that declares *all* the funding or sources of support (whether external or internal to your organization) received during this study, as detailed online in our guide for authors at http://journals.plos.org/plosone/s/submit-now.  Please also include the statement “There was no additional external funding received for this study.” in your updated Funding Statement.

Please include your amended Funding Statement within your cover letter. We will change the online submission form on your behalf.

**Response:** We have updated the Funding Statement in both the manuscript and cover letter to include all sources of support. The statement now explicitly includes: “There was no additional external funding received for this study.”

5. Thank you for stating the following financial disclosure:
ZZ is partially funded by his startup at The University of Texas Health Science Center at Houston, Houston, Texas, USA.

Please state what role the funders took in the study. If the funders had no role, please state: "The funders had no role in study design, data collection and analysis, decision to publish, or preparation of the manuscript."

If this statement is not correct, you must amend it as needed.
Please include this amended Role of Funder statement in your cover letter; we will change the online submission form on your behalf.

**Response:** We have included the Role of Funder statement in the manuscript and cover letter. Specifically, we state: “The funders had no role in study design, data collection and analysis, decision to publish, or preparation of the manuscript.”

6. Thank you for uploading your study's underlying data set. Unfortunately, the repository you have noted in your Data Availability statement does not qualify as an acceptable data repository according to PLOS's standards.

At this time, please upload the minimal data set necessary to replicate your study's findings to a stable, public repository (such as figshare or Dryad) and provide us with the relevant URLs, DOIs, or accession numbers that may be used to access these data. For a list of recommended repositories and additional information on PLOS standards for data deposition, please see [https://journals.plos.org/plosone/s/recommended-repositories](https://track.editorialmanager.com/CL0/https:%2F%2Fjournals.plos.org%2Fplosone%2Fs%2Frecommended-repositories/1/010f019d260e1bd0-7e7c4d37-f4e0-493d-a7b0-e33ea24ac77f-000000/k2dcXXqnAJlb0GHi08k-jSNOBFVuiTj13zgj4qf0TPM=256).

**Response:** The dataset used in this study is publicly available from Kaggle. As per PLoS guidelines, we have provided direct access details and clarified that no restrictions apply.

7. If the reviewer comments include a recommendation to cite specific previously published works, please review and evaluate these publications to determine whether they are relevant and should be cited. There is no requirement to cite these works unless the editor has indicated otherwise.

**Response:** Thanks for this reminder. No, the reviewers have not suggested any references to cite.

**Reviewers' comments:**

**Reviewer #1:**

This paper develops and evaluates ensemble learning models, particularly stacking, for lung cancer risk prediction using lifestyle and clinical data. Multiple base and ensemble methods are compared across original, balanced, and upsampled datasets to assess predictive performance. SHAP and LIME are applied to interpret the models and understand the contribution of individual factors.

Major Concerns:-

1. The manuscript does not clearly explain whether SMOTE was applied strictly within each training fold during k-fold cross-validation. If synthetic samples were generated before splitting or shared across folds, this could introduce data leakage and artificially inflate performance.

**Response:** We appreciate the reviewer’s careful observation regarding the potential for data leakage when applying SMOTE in conjunction with cross-validation. This is indeed a critical methodological concern.

In our current implementation, SMOTE-based resampling was applied prior to cross-validation, resulting in three static datasets (original, balanced, and fully upsampled), on which stratified 10-fold cross-validation was subsequently performed. We clarify that SMOTE was applied to balanced and fully upsampled versions. While in balanced set, SMOTE was used to upsample only the minor class, in the fully upsampled set, SMOTE was used to upsample both classes. We agree that this differs from the strict “within-fold” resampling protocol typically recommended to completely eliminate any possibility of information leakage.

Our decision was driven by a practical constraint: the original dataset is extremely small and highly imbalanced, with only 39 samples in the minority class. Applying SMOTE strictly within each fold would lead to folds with very few minority samples, making both model training and validation unstable and highly sensitive to sampling variance. To address this, we constructed augmented datasets to enable more reliable learning and comparative evaluation across ensemble strategies.

That said, the reviewer’s concern is valid: performing resampling before cross-validation can, in principle, introduce optimistic bias due to shared synthetic structure across folds. We have taken several steps to mitigate and assess this risk:

- We evaluated models across three dataset regimes (original, SMOTE-balanced, and fully upsampled), rather than relying on a single augmented dataset.
- We conducted learning curve analysis, which showed that simpler models (e.g., voting) exhibited overfitting on the upsampled data, while the stacking model maintained stable training–validation alignment.
- Performance improvements were consistent across datasets, not confined to the augmented versions.

However, we acknowledge that these checks do not fully substitute for a leakage-free evaluation protocol. We have also added a discussion of the limitations of pre-CV resampling, including its potential impact on performance estimates.

2. Some models achieve near-perfect performance on the upsampled dataset, which raises concerns about overfitting.

**Response:** We thank the reviewer for this important observation. We agree that the near-perfect performance on the upsampled dataset warrants careful scrutiny for potential overfitting.

To investigate this, we analyzed the learning curves of both voting and stacking models (Fig. 14). On the original and SMOTE-balanced datasets, both models exhibited closely aligned training and validation curves, indicating stable learning and good generalization.

On the fully upsampled dataset, however, a clear difference emerged: the voting model showed noticeable divergence between training and validation performance, suggesting overfitting to the synthetic data. In contrast, the stacking model maintained close alignment between the curves, indicating more stable generalization even under aggressive upsampling.

We also relied on stratified cross-validation to reduce variance in performance estimates. Importantly, the strong performance of the stacking model was not limited to the upsampled dataset—it remained consistently high across the original and balanced datasets as well.

That said, we acknowledge that performance on heavily upsampled data can be optimistic. We have added an explicit discussion on this in Section 8.

3. Adding to the previous point, the models are evaluated on variations of a single dataset without testing on an independent external cohort, limiting generalizability.

**Response:** We thank the reviewer for highlighting this important limitation. We agree that evaluation on an independent external cohort would provide a stronger assessment of generalizability. However, we could not obtain an external dataset with similar parameters for validation. Parameter matching is crucial in our study, as we identified the contribution of specific predicate variables to lung cancer.

As an alternative approach, this study evaluated all models on variations of a single dataset (original, SMOTE-balanced, and fully upsampled). These variants were designed to test model behavior under different sample distributions and data volumes, rather than to serve as true external validation. While the consistent performance of the stacking model across these settings suggests robustness to data imbalance and sampling variations, it does not fully establish generalizability to unseen populations.

We acknowledge that the absence of an external or multi-institutional dataset limits the clinical applicability of the current findings. We will revise the manuscript to explicitly state this limitation.

As part of future work, we plan to explore extensively for similar datasets and validate the proposed model on independent datasets from different sources to assess its real-world generalization capability.

4. The strategy of upsampling both minority and majority classes to substantially enlarge the dataset is unconventional and may distort real-world class distributions, affecting generalizability

**Response:** We thank the reviewer for this insightful observation. We agree that upsampling both minority and majority classes is unconventional and does not reflect real-world class distributions.

Our intention with the fully upsampled dataset was not to model realistic prevalence, but to create a controlled setting to examine how different ensemble methods behave when sufficient data volume is available. Given the very small size of the original dataset, this approach allowed us to study model capacity, stability, and comparative performance at higher sample densities.

This approach resulted in overall improved efficacy for the ensemble models in terms of not only standard evaluation metrics but also statistical significance, and XAI-based interpretability.

However, we emphasize that this upsampled dataset was used strictly for methodological exploration, alongside the original and SMOTE-balanced datasets, rather than as a proxy for real-world deployment. Importantly, the models were also evaluated on the original dataset, which preserves the natural class distribution.

Nevertheless, we are not denying that training on artificially expanded data may distort the underlying data distributions and lead to overly optimistic performance estimates. We have added this aspect in the limitation section of the revised manuscript.

5. Although performance differences between models are reported, no statistical tests (e.g. paired tests across folds) are provided to show whether differences are statistically significant.

**Response:** We thank the reviewer for this important suggestion. We agree that statistical evaluation is necessary to support the significance of performance differences between models.

To address this, we conducted a non-parametric statistical analysis. Specifically, we performed a multiple-group (one-vs-all) comparison, where the stacking and voting models (with selected base learners) were evaluated against all base models. In addition, pairwise comparisons were carried out between the stacking and voting models.

These tests were performed across cross-validation folds, and the results confirm that the performance improvements achieved by the stacking model are statistically significant rather than due to random variation. The statistical significance is included in Section 6.

6. SHAP results indicate relatively low importance for smoking compared to symptoms, which contradicts epidemiological evidence and may reflect dataset bias that requires further clarification.

**Response:** We thank the reviewer for this important and valid observation. We agree that the relatively low importance of smoking in the SHAP analysis appears counterintuitive given its well-established role as the primary risk factor for lung cancer.

We would like to clarify that this behaviour has been explicitly analysed and discussed in the revised manuscript. Our findings suggest that this apparent discrepancy is primarily driven by the nature of the dataset and feature interactions rather than a contradiction of established epidemiological evidence. Specifically, the dataset is more symptom-oriented and reflects patient-level clinical manifestations rather than long-term exposure history. As a result, the model tends to assign higher importance to features that directly capture the physiological consequences of smoking (e.g., yellow fingers, shortness of breath) rather than the smoking variable itself.

This effect is further reinforced by multicollinearity, where the predictive signal of smoking is distributed across highly correlated downstream features. Consequently, SHAP—being a model-specific attribution method—allocates importance to the features that the model directly relies on for prediction, rather than to causal risk factors. This explains why smoking appears less prominent despite its underlying causal role.

Importantly, our interpretability analysis does not treat these findings in isolation. We provide a detailed clinical discussion, supported by relevant literature, showing that several top-ranked features (e.g., fatigue, alcohol consumption, swallowing difficulty) have plausible biological or clinical associations with lung cancer, while also critically examining deviations such as the reduced importance of smoking and age. These deviations are explicitly acknowledged as potential indicators of dataset bias and feature representation limitations.

We have further clarified in the manuscript that model-derived feature importance should not be interpreted as a direct reflection of epidemiological causality, but rather as a representation of patterns learned from the available data. This distinction is essential for responsible clinical interpretation.

7. The paper has a clear objective and is technically solid, but it feels slightly overextended. It covers a lot of concepts. Tightening the focus around the main goal would improve overall impact.

**Response:** We sincerely thank the reviewer for this thoughtful feedback. We agree the manuscript covers multiple components. After careful consideration, we felt they are all centered around a single objective: the development and evaluation of interpretable ensemble models—particularly voting and stacking—for lung cancer prediction.

The elements of the study are designed as sequential, interdependent steps toward this goal. The selection of base models establishes a strong foundation for ensemble construction; the comparative performance analysis demonstrates their effectiveness; statistical testing validates the reliability of the observed improvements; and the XAI analysis provides interpretability for clinical relevance. Each component is therefore not standalone, but contributes directly to strengthening the central objective of the work.

We acknowledge that the breadth of these components may give an impression of overextension; however, they are included to provide a complete and rigorous evaluation of the proposed approach.

Furthermore, we carefully reviewed the complete manuscript, trying to compact the writing wherever possible. For example, we minimised the text of Table 3, which was too verbose earlier. We also removed Figure 1 (earlier), which merely represented the methodology, described in Section 3.

We sincerely hope the reviewer is okay with our explanation above. If this reviewer still prefers us to shorten the manuscript writing, we will try in the next version.

8. How is the confidence score defined here?

**Response:** We thank the reviewer for this important point. We acknowledge that the term “confidence score” was not clearly defined and may have caused confusion.

In our study, the reported “confidence score” is not a probabilistic measure of prediction certainty. Instead, it refers to a Composite Performance Score (CCS), computed as the average of multiple evaluation metrics (Accuracy, Precision, Recall, F1-score, MCC, Kappa, and AUC) across the three dataset variants. This score was introduced solely to provide a unified basis for comparing model performance.

We agree that using the term “confidence” for this aggregate metric is potentially misleading. In the revised manuscript, we will replace it with “Composite Performance Score (CCS)” and clearly distinguish it from prediction probabilities.

We revised the corresponding text (the description of Fig. 27) to ensure that the interpretation reflects the overall model performance rather than prediction confidence.

**Reviewer #2:**

I have following concerns:

1. Data leakage risk: Synthetic data (SMOTE) was applied before splitting, potentially causing data leakage. Perform augmentation after train-test split to ensure synthetic samples don't influence validation folds. This undermines reported performance generalizability.

**Response:** We thank the reviewer for highlighting this critical issue. We fully agree that applying SMOTE before data splitting can introduce data leakage and lead to overly optimistic performance estimates.

In our current implementation, SMOTE-based resampling was applied prior to cross-validation, resulting in augmented datasets on which stratified k-fold cross-validation was performed. We acknowledge that this does not follow the recommended practice of applying resampling strictly within each training fold.

This design choice was driven by the extremely small and imbalanced nature of the original dataset (only 39 samples in the minority class), where fold-wise resampling led to unstable training and high variance across folds. To enable meaningful model training and comparison, we constructed balanced, upsampled datasets to serve as controlled experimental settings.

However, we recognize that this approach may introduce optimistic bias and limit the reliability of the reported performance. We revised the manuscript to clearly state this limitation and avoid overstating generalizability. We added a discussion of a leakage-free evaluation in the limitations section.

2. Missing hyperparameter details: Table 5 lists hyperparameters as empty placeholders. Provide actual tuned values (e.g., learning rate, n_estimators, depth) for reproducibility. Without these, other researchers cannot replicate your optimization process.

**Response:** We thank the reviewer for this valuable comment. We would like to clarify that the hyperparameter values are already provided in the manuscript (Table 7), where the tuned configurations for all constituent models (e.g., learning rate, number of estimators, depth, etc.) are explicitly listed.

3. Overstated accuracy claims: 99.94% accuracy on upsampled data is unrealistic for clinical deployment. Emphasize the actual dataset results (92.79%) as more realistic, and clarify that synthetic augmentation inflates metrics beyond real-world expectations.

**Response:** We thank the reviewer for this valuable comment. We agree that the near-perfect accuracy (99.94%) obtained on the upsampled dataset is not representative of real-world clinical performance and may be influenced by synthetic data augmentation.

In the manuscript, we have explicitly addressed this concern by distinguishing between the performance across different dataset configurations. In particular, we note that while the upsampled dataset is useful for analyzing model behavior under increased data volume and balanced class distributions, it does not reflect real-world clinical conditions. Accordingly, we emphasize that the performance of the stacking model on the original dataset (93.53% accuracy) provides a more realistic estimate of its practical applicability.

We also discussed the limitations associated with synthetic data augmentation, including the potential for optimistic performance estimates and distortion of real-world data distributions. Furthermore, our learning curve analysis highlights that simpler models (e.g., voting) exhibit overfitting on the upsampled dataset, whereas the stacking model demonstrates relatively more stable generalization, reinforcing the need for cautious interpretation of results derived from synthetic data.

In Figures 15 and 20, we clearly distinguished experimental performance under augmented conditions from more realistic performance on the original dataset, and we avoid treating synthetic data results as indicative of clinical readiness in the discussion.

4. No statistical significance testing: Compare models using statistical tests (e.g., McNemar’s, paired t-tests) to confirm that observed performance differences are significant rather than due to random variation. Currently, claims of "outperformance" lack statistical support.

**Response:** We thank the reviewer for this important suggestion. We agree that statistical significance testing is essential to support claims of model outperformance.

To address this, we performed a non-parametric statistical analysis across cross-validation folds. Specifically, we conducted a multiple-group (one-vs-all) comparison, where the stacking model (with selected base learners) was evaluated against all base models and the voting ensemble. In addition, pairwise comparisons were carried out between the stacking and voting models.

These tests confirm that the observed performance improvements of the stacking model are statistically significant and not due to random variation.

5. Limited dataset generalizability: Single small dataset (309 samples) severely limits external validity. Acknowledge this more prominently and discuss plans for multi-center validation; otherwise, clinical applicability claims are premature.

**Response:** We thank the reviewer for this important observation. We fully agree that the use of a single, relatively small dataset (309 samples) limits the external validity and generalizability of our findings.

In the current study, our primary objective was to explore the effectiveness of ensemble learning methods and XAI techniques under constrained data conditions. While the results demonstrate consistent performance across different dataset variants, we acknowledge that this does not substitute for validation on independent or multi-center datasets.

We revised the manuscript to highlight this limitation and avoid overstating clinical applicability more prominently. In particular, we clarified that the proposed model should be considered as a preliminary framework rather than a deployment-ready solution.

As part of future work, we plan to evaluate the model on larger, multi-institutional datasets to assess its robustness across diverse populations and clinical settings.

6. XAI integration superficial: While SHAP/LIME are applied, clinical interpretation is shallow. Connect feature importance to actionable clinical workflows—how would a physician use these explanations for treatment decisions? Deeper clinical translation needed.

**Response:** We thank the reviewer for this valuable comment and suggestion. We agree that, beyond identifying important features, it is essential to clearly articulate how these explanations translate into actionable clinical workflows.

In the manuscript, we provide a detailed interpretability analysis using SHAP and LIME, linking model-derived feature importance to established clinical knowledge and critically examining both alignments and deviations. We also demonstrate that SHAP and LIME provide consistent and complementary insights, with strong agreement on key predictors such as fatigue and alcohol consumption, reinforcing the clinical relevance of the learned patterns.

We further discuss how these explanations can support clinical reasoning—for example, by enabling risk stratification, highlighting patient-specific contributing factors, and allowing clinicians to assess the plausibility of model predictions before acting on them. However, we acknowledge that the current study does not provide a dedicated user interface or deployment framework through which clinicians can directly interact with these explanations in a seamless and user-friendly manner.

This represents an important limitation in terms of practical usability. To address this, we have identified the development of an intuitive, clinician-oriented interface as part of our future work, where model outputs can be presented in a structured and actionable format suitable for real-world clinical settings.

7. Contradiction in recall discussion: Stacking recall (99.47%) is reported as inferior to some base models, yet conclusions claim superior overall performance. Address this trade-off transparently—high accuracy may mask clinically relevant recall differences.

**Response:** We thank the reviewer for highlighting this important point. We agree that the trade-off between evaluation metrics, particularly recall, should be discussed more transparently.

While the stacking model achieves the highest overall performance across most metrics (accuracy, precision, F1-score, MCC, and AUC), its recall (99.47%) is slightly lower than that of some individual base models. This reflects a typical trade-off in classification tasks, where optimizing for overall performance can lead to marginal reductions in sensitivity.

We acknowledge that, in a clinical context such as lung cancer detection, recall (sensitivity) is critically important, as false negatives can have serious consequences. Therefore, even small differences in recall should not be overlooked.

In the discussion section, we added one paragraph explicitly discussing this trade-off between recall and other performance metrics.

8. Runtime analysis irrelevant: Execution time comparisons (seconds) are irrelevant for non-time-sensitive cancer prediction. Remove or reframe to focus on computational complexity or scalability for deployment in resource-constrained settings.

**Response:** We thank the reviewer for this insightful comment. We agree that runtime is not a primary concern in clinical decision-making for lung cancer prediction, where accuracy and reliability are paramount.

However, we included runtime analysis to provide insight into the computational efficiency and scalability of the proposed models, particularly in scenarios involving larger datasets or deployment in resource-constrained environments. As shown in Fig. 28, while the stacking model achieves superior predictive performance, it incurs higher computational cost compared to simpler base models and voting ensembles.

We acknowledge that this aspect was not sufficiently contextualized in the manuscript. In the revised version, we have reframed the discussion to emphasize computational trade-offs and scalability, rather than clinical relevance. We clarified that runtime is a secondary consideration, relevant mainly for large-scale or real-time deployment settings

9. Acronym overload without mapping: Table 1 (acronyms) appears incomplete in the PDF. Ensure all acronyms are defined at first use and the table is fully legible; reviewers cannot assess readability with missing definitions.

**Response:** We thank the reviewer for highlighting this issue. We acknowledge that the acronym table in the original manuscript was not clearly presented, which may have affected readability.

To address this, we have removed the acronym table and ensured that all acronyms are explicitly defined at their first occurrence in the text. This approach improves clarity and allows readers to understand terminology in context without relying on a separate table.

10. Overly complex model selection: Justify why LR, CB, XGB, RF, ET were selected for final stacking. The "top 5" selection based on accuracy alone ignores diversity—ensemble benefits from different error patterns, not just individual performance.

**Response:** We thank the reviewer for this insightful comment. We agree that effective ensemble design should consider not only individual model performance but also diversity in learning behavior.

In our study, we initially evaluated fifteen models across three categories: (i) traditional machine learning (LR, NB, KNN, SVM, LDA), (ii) boosting methods (CB, GB, XGB, AB, LGBM), and (iii) bagging techniques (DT, ET, BDT, BME, RF).

For ensemble construction, we adopted a two-stage strategy. First, a top-5 selection based on performance was used to identify strong candidate models for each dataset. However, we did not rely solely on this criterion for the final design.

In the second stage, we explicitly incorporated model diversity by selecting a heterogeneous set of base learners—LR, CB, XGB, RF, and ET—for the final stacking model. These models represent fundamentally different learning paradigms: linear (LR), boosting-based (CB, XGB), and bagging-based ensembles (RF, ET). This cross-family selection was intended to capture diverse decision boundaries and complementary error patterns.

Empirically, this combination showed more stable and consistent performance across all dataset variants compared to purely performance-driven selections. The stacking framework was then able to effectively leverage this diversity through the meta-learner, leading to improved overall results.

We acknowledge that this rationale was not sufficiently emphasized in the manuscript. In the revised version, we explicitly clarified (in section 5.3.5) that the final model selection balances both individual performance and algorithmic diversity, rather than relying on accuracy alone.

11. Missing comparison with simple baselines: No comparison against logistic regression without ensemble or simple decision trees. Demonstrate added complexity yields meaningful clinical improvement; otherwise, parsimonious models may be preferable for deployment.

**Response:** We thank the reviewer for this important suggestion. We agree that comparison with simpler baseline models is essential to justify the added complexity of ensemble approaches.

In our study, we explicitly evaluated several baseline models, including logistic regression and decision tree-based methods, as part of the initial set of fifteen models. Their performance is reported and compared with ensemble models in the results section. While baseline models such as logistic regression achieved reasonable performance, the ensemble methods—particularly the stacking model—consistently outperformed them across multiple evaluation metrics (including F1-score, MCC, and AUC), indicating a meaningful and not merely marginal improvement.

In addition, we conducted statistical significance testing to validate these performance differences. Specifically, we performed a non-parametric multiple-group (one-vs-all) analysis comparing the stacking model (with selected base learners) against all base models and the voting ensemble, followed by pairwise comparisons between stacking and voting. The results confirm that the improvements achieved by the stacking model are statistically significant and not due to random variation.

At the same time, we acknowledge that simpler models offer advantages in terms of interpretability and ease of deployment. Therefore, model selection in practice should balance performance gains with simplicity, depending on clinical requirements.
