# Supplementary material for: Lung cancer risk prediction using interpretable ensemble models on lifestyle and clinical data
Source: PLoS One. 2026 Sep 3;21(9):e0357291. doi: 10.1371/journal.pone.0357291 (PMC13541198; doi:10.1371/journal.pone.0357291)
Supplement: Supplementary file 2 [file pone.0357291.s003.docx]

Manuscript ID: PONE-D-25-51253R1

Title: Lung Cancer Risk Prediction Using Interpretable Ensemble Models on Lifestyle and Clinical Data

We thank the reviewers and editor for valuable comments. We have carefully addressed these comments and revised the manuscript accordingly. For ease of evaluation, all modifications made in the revised manuscript have been highlighted in yellow. We thank the editor and reviewers for their constructive feedback, which has helped us improve the quality, clarity, and rigor of the manuscript. A detailed point-by-point response to all comments is provided below.

1. Add leakage‑free validation as a robustness check. Even with current limitations, perform one additional experiment: apply SMOTE strictly inside each training fold of a single train/test split. Report whether stacking’s performance drops significantly. If it does, acknowledge this openly; if not, it strengthens your claims.

**Response:** We thank the reviewer for this constructive suggestion. We agree that evaluating the models under a leakage-free resampling protocol provides a more rigorous assessment of their robustness.

Following the reviewer's recommendation, we conducted an additional experiment in which SMOTE was applied only after data splitting, with synthetic samples generated exclusively from the training data. The performance of the voting and stacking ensembles obtained under this protocol was then compared with the results from our original experimental setting. The results are presented in the newly added Table 9 and discussed in Section 5.4.2.

Interestingly, the performance of the proposed stacking model did not deteriorate under the stricter evaluation protocol. For the balanced dataset, the selected-5 stacking model improved from 93.78% to 95.67% accuracy, while its F1-score increased from 93.98% to 95.87%. A similar trend was observed for the voting models. For the upsampled dataset, the stacking ensemble continued to achieve the strongest overall performance, attaining 99.94% accuracy, 99.47% recall, and 99.74% AUC when SMOTE was applied after splitting.

An important observation is that the relative ranking of the models remained unchanged. Across both evaluation settings, stacking consistently outperformed voting and maintained its advantage over the constituent base learners. These results indicate that the performance gains reported in the manuscript are not dependent on a particular SMOTE application strategy and persist under a leakage-free resampling protocol.

We appreciate the reviewer for raising this point, as the additional experiment has strengthened the validation of the proposed framework and has been incorporated into the revised manuscript.

2. Remove or heavily downplay the upsampled‑dataset results. The 99.94% accuracy is unrealistic and harms credibility. Keep it only in a supplementary section or as a footnote. Emphasize original‑dataset performance (≈93%) as the primary finding in abstract, results, and conclusion.

**Response:** We thank the reviewer for this important observation. We agree that the near-perfect performance obtained on the upsampled dataset should not be interpreted as representative of real-world clinical deployment.

The purpose of including the balanced and upsampled datasets was to systematically examine the effect of class distribution and dataset size on model performance, rather than to establish deployment-level performance claims. For this reason, we prefer to retaine the results for all three dataset variants to provide a complete comparative evaluation framework.

At the same time, we have taken several steps to ensure that the findings are not overly centered on the upsampled results. In the revised manuscript, the discussion, limitations, and conclusions explicitly acknowledge that synthetic augmentation may lead to optimistic performance estimates and that the results obtained on the original dataset provide a more realistic assessment of practical applicability. We also expanded the state-of-the-art comparison (Table 10) to include results from all three dataset variants, whereas the previous version focused primarily on the upsampled dataset.

Accordingly, the manuscript distinguishes between experimental findings obtained under augmented-data conditions and those obtained on the original dataset, enabling readers to interpret the reported performance in the appropriate context. This update is reflected in the Abstract, Discussion and Conclusion sections.

3. Run a controlled experiment to explain the smoking paradox. Use SHAP on a simplified model (e.g., only smoking + yellow fingers) to show how multicollinearity redistributes importance. Present this as a small additional figure or table to convincingly demonstrate the effect.

**Response:** We thank the reviewer for this insightful observation and suggestion. We agree that the relatively low SHAP importance assigned to smoking, despite its established role as the dominant risk factor for lung cancer, warrants further investigation.

Rather than constructing a simplified two-feature model, we performed a broader analysis to examine whether this behavior persists across different ensemble architectures and dataset configurations. Specifically, we analyzed feature importance for both the voting and stacking models on the original, balanced, and upsampled datasets (Section 5.3.5). Interestingly, in all cases, smoking exhibited relatively low importance, while smoking-associated manifestations such as yellow fingers, chronic cough, and shortness of breath consistently received higher importance scores. This indicates that the observation is not specific to a particular model or dataset variant.

To further investigate this phenomenon, we conducted a multicollinearity analysis (Section 8) using the VIF. The results revealed substantial correlations among smoking-related predictors, with smoking (VIF = 11.16), yellow fingers (VIF = 19.08), chronic cough (VIF = 17.47), shortness of breath (VIF = 18.78), and alcohol consumption (VIF = 17.79) all exhibiting high VIF values. These findings suggest that the predictive information associated with smoking is distributed across multiple correlated variables.

Consequently, attribution methods such as SHAP tend to distribute explanatory importance among correlated predictors rather than assigning the entire contribution to a single feature. Therefore, the relatively lower importance assigned to smoking should not be interpreted as evidence of reduced clinical significance, but rather as a consequence of multicollinearity and the representation of smoking-related effects through correlated clinical manifestations. We have incorporated this analysis and discussion into the manuscript and added the corresponding VIF results to support this interpretation.

4. “Add a quantitative diversity metric for the stacking base learners. Compute pairwise Q‑statistics or correlation of errors among LR, CB, XGB, RF, ET. Show that diversity (not just individual accuracy) contributes to stacking’s gain. This directly addresses Reviewer #2’s concern about model selection.”

**Response:** We thank the reviewer for this valuable suggestion. We agree that the effectiveness of a stacking ensemble depends not only on the individual predictive strength of its constituent models but also on the diversity of their prediction behaviours.

To address this point, we performed an additional diversity analysis on the five selected base learners (LR, CB, XGB, RF, and ET) using pairwise Q-statistics and error-correlation measures. While our original statistical analysis demonstrated that the proposed stacking ensemble significantly outperformed many baseline models, it did not explicitly quantify the complementarity among the selected learners.

The diversity analysis revealed that the selected models exhibit a balanced degree of diversity despite their strong individual performance. For example, RF and ET showed the highest agreement (Q = 0.91, error correlation = 0.84), which is expected given their shared bagging-based architecture. In contrast, combinations involving LR and tree-based ensembles (e.g., LR–ET and LR–RF) exhibited substantially lower agreement, indicating greater diversity in decision boundaries and error patterns. Most remaining pairs demonstrated moderate diversity, suggesting that the selected learners provide complementary predictive information rather than redundant predictions.

These findings support our model-selection strategy and indicate that the performance gains of the stacking framework arise not only from the accuracy of the constituent learners but also from their diversity. We have incorporated this analysis into the manuscript and added a dedicated table reporting the pairwise diversity metrics.

5. Provide a concrete clinical workflow sketch. Add a short paragraph or a simple diagram showing: patient intake → model outputs (risk score + top 3 SHAP/LIME factors) → clinician review → recommended action (e.g., low‑dose CT referral). This makes XAI integration actionable.

**Response:** We thank the reviewer for this valuable suggestion. We agree that the practical use of explainable AI in a clinical setting should be more explicitly illustrated. To address this point, we have added a conceptual clinical workflow figure (Figure 35) and accompanying discussion describing how the proposed framework could be integrated into routine clinical practice. The workflow demonstrates how patient demographic, lifestyle, and symptom-related information is processed by the prediction model to generate both a risk score and SHAP/LIME-based explanations. These outputs are then reviewed by clinicians and can support decisions regarding additional assessment, low-dose CT screening, specialist referral, or routine follow-up.

6. Move runtime analysis to supplementary material. Since reviewers called it irrelevant for non‑time‑sensitive prediction, relocate Fig. 27 and related text to an appendix. Replace it with a brief statement on computational complexity (e.g., training vs. inference time) to keep focus on clinical utility.

**Response:** We thank the reviewer for this valuable suggestion. We agree that detailed runtime comparisons are not central to the primary objective of this study, which is to evaluate the predictive performance and interpretability of ensemble models for lung cancer risk prediction.

Accordingly, we have removed the runtime comparison figure and the associated detailed analysis from the manuscript. Instead, we briefly discuss the computational implications of the proposed models in the Discussion section. Specifically, we acknowledge the trade-off between predictive performance and computational cost, noting that the stacking ensemble achieves superior accuracy at the expense of increased computational complexity. However, since lung cancer risk prediction is not a real-time application, the additional computational overhead is unlikely to be a major limitation in typical clinical environments. We also note that computational efficiency may become relevant in large-scale screening programs or resource-constrained deployment settings.
