# Supplementary material for: Lung cancer risk prediction using interpretable ensemble models on lifestyle and clinical data
Source: PLoS One. 2026 Sep 3;21(9):e0357291. doi: 10.1371/journal.pone.0357291 (PMC13541198; doi:10.1371/journal.pone.0357291)
Supplement: Supplementary file 3 [file pone.0357291.s004.docx]

**Manuscript ID:** PONE-D-25-51253R2

**Title:** *Lung Cancer Risk Prediction Using Interpretable Ensemble Models on Lifestyle and Clinical Data*

We thank the Academic Editor for the careful evaluation of our revised manuscript and for the additional constructive comments. We have addressed each of the points raised and revised the manuscript accordingly. For ease of review, all modifications in the revised manuscript have been highlighted in yellow. A detailed point-by-point response to each comment is provided below.

**Additional Editor Comments:**

The manuscript has improved substantially and most of the technical concerns have been addressed. However, before acceptance, the authors should complete the following revisions to ensure that the claims are appropriately calibrated and the study is fully reproducible:

1. The 99.94% accuracy should be removed from the Abstract or clearly labeled as a synthetic-data sensitivity analysis, not as a clinically realistic performance estimate. The original-dataset performance of 93.53% should be emphasized as the primary result throughout the Abstract, Results, Discussion, and Conclusion.

**Response**: We appreciate the Editor's suggestion and have revised the manuscript accordingly. The explicit mention of the 99.94% accuracy has been removed from both the Abstract and the Conclusion to avoid presenting the performance on the synthetically upsampled dataset as a clinically realistic outcome. Throughout the manuscript, we now place greater emphasis on the model's performance on the original dataset (93.53% accuracy) as the most representative estimate of its practical applicability. The results obtained on the balanced and upsampled datasets are retained only in the Results (Section 5) for comparative analysis to illustrate the effect of different data augmentation strategies, and are discussed with appropriate caution as sensitivity analyses rather than indicators of expected real-world clinical performance.

2. The response states that SMOTE was applied after data splitting and only to the training data, but the manuscript should explicitly confirm that SMOTE and all preprocessing steps were applied strictly within the training fold only, with validation/test data left completely untouched during model fitting, tuning, and resampling. This is important because the original reviewer request specifically asked for SMOTE inside the training fold.

**Response:** Thank you for this valuable comment. We would like to clarify that the leakage-free SMOTE protocol was implemented as an additional robustness analysis in response to Reviewer 1's recommendation. Specifically, an additional experiment was conducted in which SMOTE was applied only after data splitting and exclusively to the training partition within each fold, while the corresponding validation fold remained completely untouched throughout resampling, model training, hyperparameter tuning, and performance evaluation. This protocol and the corresponding results are described in Section 5.4.2 and summarized in Table 9. The purpose of this additional experiment was to verify whether the performance of the proposed ensemble models remained stable under a stricter evaluation protocol. The results show that the stacking model did not exhibit any performance degradation. For the balanced dataset, the selected-5 stacking model improved from 93.78% to 95.67% accuracy, while its F1-score increased from 93.98% to 95.87%. Likewise, for the upsampled dataset, the stacking model continued to achieve the strongest overall performance, attaining 99.94% accuracy, 99.47% recall, and 99.74% AUC. These results indicate that the conclusions of the study remain unchanged under the leakage-free validation protocol.

3. The added workflow is useful, but the manuscript should clearly state that the model is not ready for clinical deployment or low-dose CT referral decisions without prospective and external validation. The workflow should be presented as a conceptual decision-support framework only, not a clinical recommendation. Please temper all clinical-implementation language.

**Response:** Thank you for this valuable observation. We agree that the proposed framework should not be presented as being ready for routine clinical use. Accordingly, we have revised the manuscript to consistently describe the proposed workflow as a **conceptual decision-**support framework rather than a clinical implementation pathway. The discussion has been updated to clarify that the workflow illustrates a potential future integration of explainable AI into clinical practice and should not be interpreted as a recommendation for current clinical deployment or referral decisions. To further reinforce this distinction, the title of Fig. 35 have also been revised to explicitly identify it as a conceptual decision-support workflow. In addition, the limitations section already states that prospective studies and independent external validation are required before the proposed framework can be considered for routine clinical use.

4. The authors should provide sufficient implementation details, including hyperparameters, random seeds, train/test split strategy, SMOTE settings. This will help ensure that the reported results can be independently reproduced.

**Response:** Thank you for this helpful suggestion. To improve the reproducibility of the study, we have expanded the implementation details in the revised manuscript. The manuscript now explicitly describes the 75%/25% train-test split strategy, the use of stratified 10-fold cross-validation for model optimization, the SMOTE configuration (sampling_strategy='auto', k_neighbors=5), and the leakage-free robustness experiment in which SMOTE was applied exclusively to the training data after data splitting. The optimized hyperparameter values of all models are reported in Table 4. In addition, to facilitate independent verification and reproduction of the results, we have made the complete implementation publicly available through a GitHub repository, the link to which is provided in the Declaration section of the manuscript.

5. The authors should explicitly state that the study relies on a single public Kaggle dataset, lacks external validation, may contain dataset-specific patterns, and that results from balanced/upsampled datasets may overestimate real-world performance.

**Response:** Thank you for this helpful suggestion. We have revised the limitations section to make these points more explicit. The manuscript now clearly states that the study is based on a single publicly available Kaggle dataset, which may contain dataset-specific characteristics that limit generalizability. We also reiterate that the proposed models have not undergone external validation and that the current findings should therefore be regarded as preliminary. In addition, we have strengthened the discussion on synthetic data augmentation by explicitly stating that the results obtained on the balanced and particularly the upsampled datasets should be interpreted as comparative analyses under augmented data conditions and not as estimates of expected real-world clinical performance.

6. Please conduct final language polishing.

**Response:** Thank you for the suggestion. The manuscript has been carefully proofread, and minor revisions have been made to improve grammar, clarity, and readability while preserving the technical content and intended meaning.
