# Supplementary material for: Lung cancer risk prediction using interpretable ensemble models on lifestyle and clinical data
Source: PLoS One. 2026 Sep 3;21(9):e0357291. doi: 10.1371/journal.pone.0357291 (PMC13541198; doi:10.1371/journal.pone.0357291)
Supplement: Peer review history

# Peer review: Decision Letter 0

**DOI:** 10.1371/journal.pone.0357291.r001

**Authors:** Amgad Muneer

25 Mar 2026

Dear Dr. Dutta Pramanik,

Thank you for submitting your manuscript to PLOS ONE. After careful consideration, we feel that it has merit but does not fully meet PLOS ONE’s publication criteria as it currently stands. Therefore, we invite you to submit a revised version of the manuscript that addresses the points raised during the review process.

Please submit your revised manuscript by May 09 2026 11:59PM. If you will need more time than this to complete your revisions, please reply to this message or contact the journal office at plosone@plos.org. When you're ready to submit your revision, log on to https://www.editorialmanager.com/pone/ and select the 'Submissions Needing Revision' folder to locate your manuscript file.

- A letter that responds to each point raised by the academic editor and reviewer(s). You should upload this letter as a separate file labeled 'Response to Reviewers'.
- A marked-up copy of your manuscript that highlights changes made to the original version. You should upload this as a separate file labeled 'Revised Manuscript with Track Changes'.
- An unmarked version of your revised paper without tracked changes. You should upload this as a separate file labeled 'Manuscript'.

If you would like to make changes to your financial disclosure, please include your updated statement in your cover letter. Guidelines for resubmitting your figure files are available below the reviewer comments at the end of this letter.

If applicable, we recommend that you deposit your laboratory protocols in protocols.io to enhance the reproducibility of your results. Protocols.io assigns your protocol its own identifier (DOI) so that it can be cited independently in the future. For instructions see: https://journals.plos.org/plosone/s/submission-guidelines#loc-laboratory-protocols. Additionally, PLOS ONE offers an option for publishing peer-reviewed Lab Protocol articles, which describe protocols hosted on protocols.io. Read more information on sharing protocols at https://plos.org/protocols?utm_medium=editorial-email&utm_source=authorletters&utm_campaign=protocols.

As the corresponding author, your ORCID iD is verified in the submission system and will appear in the published article. PLOS supports the use of ORCID, and we encourage all coauthors to register for an ORCID iD and use it as well. Please encourage your coauthors to verify their ORCID iD within the submission system before final acceptance, as unverified ORCID iDs will not appear in the published article. *Only*  the individual author can complete the verification step; PLOS staff *cannot*  verify ORCID iDs on behalf of authors.

We look forward to receiving your revised manuscript.

Kind regards,

Amgad Muneer

Academic Editor

PLOS One

Journal Requirements:

When submitting your revision, we need you to address these additional requirements.

1. Please ensure that your manuscript meets PLOS ONE's style requirements, including those for file naming. The PLOS ONE style templates can be found at

https://journals.plos.org/plosone/s/file?id=wjVg/PLOSOne_formatting_sample_main_body.pdf and

https://journals.plos.org/plosone/s/file?id=ba62/PLOSOne_formatting_sample_title_authors_affiliations.pdf

2. Please note that PLOS One has specific guidelines on code sharing for submissions in which author-generated code underpins the findings in the manuscript. In these cases, we expect all author-generated code to be made available without restrictions upon publication of the work. Please review our guidelines at https://journals.plos.org/plosone/s/materials-and-software-sharing#loc-sharing-code and ensure that your code is shared in a way that follows best practice and facilitates reproducibility and reuse.

3. We note that the grant information you provided in the ‘Funding Information’ and ‘Financial Disclosure’ sections do not match.

When you resubmit, please ensure that you provide the correct grant numbers for the awards you received for your study in the ‘Funding Information’ section.

4. Thank you for stating in your Funding Statement:

ZZ is partially funded by his startup at The University of Texas Health Science Center at Houston, Houston, Texas, USA.

Please provide an amended statement that declares *all* the funding or sources of support (whether external or internal to your organization) received during this study, as detailed online in our guide for authors at http://journals.plos.org/plosone/s/submit-now.  Please also include the statement “There was no additional external funding received for this study.” in your updated Funding Statement.

Please include your amended Funding Statement within your cover letter. We will change the online submission form on your behalf.

5. Thank you for stating the following financial disclosure:

ZZ is partially funded by his startup at The University of Texas Health Science Center at Houston, Houston, Texas, USA.

Please state what role the funders took in the study. If the funders had no role, please state: "The funders had no role in study design, data collection and analysis, decision to publish, or preparation of the manuscript."

If this statement is not correct you must amend it as needed.

Please include this amended Role of Funder statement in your cover letter; we will change the online submission form on your behalf.

6. Thank you for uploading your study's underlying data set. Unfortunately, the repository you have noted in your Data Availability statement does not qualify as an acceptable data repository according to PLOS's standards.

At this time, please upload the minimal data set necessary to replicate your study's findings to a stable, public repository (such as figshare or Dryad) and provide us with the relevant URLs, DOIs, or accession numbers that may be used to access these data. For a list of recommended repositories and additional information on PLOS standards for data deposition, please see https://journals.plos.org/plosone/s/recommended-repositories.

7. If the reviewer comments include a recommendation to cite specific previously published works, please review and evaluate these publications to determine whether they are relevant and should be cited. There is no requirement to cite these works unless the editor has indicated otherwise.

Reviewers' comments:

Reviewer's Responses to Questions

**Comments to the Author**

1. Is the manuscript technically sound, and do the data support the conclusions?

Reviewer #1: Yes

Reviewer #2: Yes

**********

2. Has the statistical analysis been performed appropriately and rigorously?

Reviewer #1: No

Reviewer #2: Yes

**********

3. Have the authors made all data underlying the findings in their manuscript fully available?

The PLOS Data policy

Reviewer #1: Yes

Reviewer #2: Yes

**********

4. Is the manuscript presented in an intelligible fashion and written in standard English?

Reviewer #1: Yes

Reviewer #2: Yes

**********

Reviewer #1: Summary: -

This paper develops and evaluates ensemble learning models, particularly stacking, for lung cancer risk prediction using lifestyle and clinical data. Multiple base and ensemble methods are compared across original, balanced, and upsampled datasets to assess predictive performance. SHAP and LIME are applied to interpret the models and understand the contribution of individual factors.

Major Concerns:-

-The manuscript does not clearly explain whether SMOTE was applied strictly within each training fold during k-fold cross-validation. If synthetic samples were generated before splitting or shared across folds, this could introduce data leakage and artificially inflate performance.

-Some models achieve near-perfect performance on the upsampled dataset, which raises concerns about overfitting.

-Adding to the previous point, the models are evaluated on variations of a single dataset without testing on an independent external cohort, limiting generalizability.

-The strategy of upsampling both minority and majority classes to substantially enlarge the dataset is unconventional and may distort real-world class distributions, affecting generalizability

-Although performance differences between models are reported, no statistical tests (e.g. paired tests across folds) are provided to show whether differences are statistically significant.

-SHAP results indicate relatively low importance for smoking compared to symptoms, which contradicts epidemiological evidence and may reflect dataset bias that requires further clarification.

-The paper has a clear objective and is technically solid, but it feels slightly overextended. It covers a lot of concepts. Tightening the focus around the main goal would improve overall impact.

-How is the confidence score defined here?

Reviewer #2: I have following concerNS :

Data leakage risk: Synthetic data (SMOTE) was applied before splitting, potentially causing data leakage. Perform augmentation after train-test split to ensure synthetic samples don't influence validation folds. This undermines reported performance generalizability.

Missing hyperparameter details: Table 5 lists hyperparameters as empty placeholders. Provide actual tuned values (e.g., learning rate, n_estimators, depth) for reproducibility. Without these, other researchers cannot replicate your optimization process.

Overstated accuracy claims: 99.94% accuracy on upsampled data is unrealistic for clinical deployment. Emphasize the actual dataset results (92.79%) as more realistic, and clarify that synthetic augmentation inflates metrics beyond real-world expectations.

No statistical significance testing: Compare models using statistical tests (e.g., McNemar’s, paired t-tests) to confirm that observed performance differences are significant rather than due to random variation. Currently, claims of "outperformance" lack statistical support.

Limited dataset generalizability: Single small dataset (309 samples) severely limits external validity. Acknowledge this more prominently and discuss plans for multi-center validation; otherwise, clinical applicability claims are premature.

XAI integration superficial: While SHAP/LIME are applied, clinical interpretation is shallow. Connect feature importance to actionable clinical workflows—how would a physician use these explanations for treatment decisions? Deeper clinical translation needed.

Contradiction in recall discussion: Stacking recall (99.47%) is reported as inferior to some base models, yet conclusions claim superior overall performance. Address this trade-off transparently—high accuracy may mask clinically relevant recall differences.

Runtime analysis irrelevant: Execution time comparisons (seconds) are irrelevant for non-time-sensitive cancer prediction. Remove or reframe to focus on computational complexity or scalability for deployment in resource-constrained settings.

Acronym overload without mapping: Table 1 (acronyms) appears incomplete in the PDF. Ensure all acronyms are defined at first use and the table is fully legible; reviewers cannot assess readability with missing definitions.

Overly complex model selection: Justify why LR, CB, XGB, RF, ET were selected for final stacking. The "top 5" selection based on accuracy alone ignores diversity—ensemble benefits from different error patterns, not just individual performance.

Missing comparison with simple baselines: No comparison against logistic regression without ensemble or simple decision trees. Demonstrate added complexity yields meaningful clinical improvement; otherwise, parsimonious models may be preferable for deployment.

**********

what does this mean?). If published, this will include your full peer review and any attached files.

If you choose “no”, your identity will remain anonymous but your review may still be made public.

**Do you want your identity to be public for this peer review?**  For information about this choice, including consent withdrawal, please see our Privacy Policy

Reviewer #1: No

Reviewer #2: No

**********

[NOTE: If reviewer comments were submitted as an attachment file, they will be attached to this email and accessible via the submission site. Please log into your account, locate the manuscript record, and check for the action link "View Attachments". If this link does not appear, there are no attachment files.]

To ensure your figures meet our technical requirements, please review our figure guidelines: https://journals.plos.org/plosone/s/figures

You may also use PLOS’s free figure tool, NAAS, to help you prepare publication quality figures: https://journals.plos.org/plosone/s/figures#loc-tools-for-figure-preparation.

NAAS will assess whether your figures meet our technical requirements by comparing each figure against our figure specifications.

# Author comment: Author response to Decision Letter 1

**DOI:** 10.1371/journal.pone.0357291.r002

13 Apr 2026

Manuscript ID: PONE-D-25-51253

Title: Lung cancer risk prediction using interpretable ensemble models on lifestyle and clinical data

We thank the editor and two reviewers for the valuable and constructive comments on our work. We tried our best to address these points during the revision. The major changes are highlighted in red text in the revised manuscript. Below, we summarize our point-to-point response.

Editorial comments:

When submitting your revision, we need you to address these additional requirements.

1. Please ensure that your manuscript meets PLOS ONE's style requirements, including those for file naming. The PLOS ONE style templates can be found at

https://journals.plos.org/plosone/s/file?id=wjVg/PLOSOne_formatting_sample_main_body.pdf and

https://journals.plos.org/plosone/s/file?id=ba62/PLOSOne_formatting_sample_title_authors_affiliations.pdf

Response: We have revised the manuscript to comply with all PLOS ONE formatting and style requirements, including file structure and naming conventions, as per the provided templates.

2. Please note that PLOS One has specific guidelines on code sharing for submissions in which author-generated code underpins the findings in the manuscript. In these cases, we expect all author-generated code to be made available without restrictions upon publication of the work. Please review our guidelines at https://journals.plos.org/plosone/s/materials-and-software-sharing#loc-sharing-code and ensure that your code is shared in a way that follows best practice and facilitates reproducibility and reuse.

Response: We have created a publicly accessible GitHub repository containing all author-generated code used in this study. The repository link has been included in the Code Availability section of the manuscript to ensure reproducibility and reuse.

3. We note that the grant information you provided in the ‘Funding Information’ and ‘Financial Disclosure’ sections do not match.

When you resubmit, please ensure that you provide the correct grant numbers for the awards you received for your study in the ‘Funding Information’ section.

Response: We have carefully reviewed and corrected the Funding Information and Financial Disclosure sections to ensure consistency. The updated funding details now accurately reflect the support received for this study.

4. Thank you for stating in your Funding Statement:

ZZ is partially funded by his startup at The University of Texas Health Science Center at Houston, Houston, Texas, USA.

Please provide an amended statement that declares *all* the funding or sources of support (whether external or internal to your organization) received during this study, as detailed online in our guide for authors at http://journals.plos.org/plosone/s/submit-now. Please also include the statement “There was no additional external funding received for this study.” in your updated Funding Statement.

Please include your amended Funding Statement within your cover letter. We will change the online submission form on your behalf.

Response: We have updated the Funding Statement in both the manuscript and cover letter to include all sources of support. The statement now explicitly includes: “There was no additional external funding received for this study.”

5. Thank you for stating the following financial disclosure:

ZZ is partially funded by his startup at The University of Texas Health Science Center at Houston, Houston, Texas, USA.

Please state what role the funders took in the study. If the funders had no role, please state: "The funders had no role in study design, data collection and analysis, decision to publish, or preparation of the manuscript."

If this statement is not correct, you must amend it as needed.

Please include this amended Role of Funder statement in your cover letter; we will change the online submission form on your behalf.

Response: We have included the Role of Funder statement in the manuscript and cover letter. Specifically, we state: “The funders had no role in study design, data collection and analysis, decision to publish, or preparation of the manuscript.”

6. Thank you for uploading your study's underlying data set. Unfortunately, the repository you have noted in your Data Availability statement does not qualify as an acceptable data repository according to PLOS's standards.

At this time, please upload the minimal data set necessary to replicate your study's findings to a stable, public repository (such as figshare or Dryad) and provide us with the relevant URLs, DOIs, or accession numbers that may be used to access these data. For a list of recommended repositories and additional information on PLOS standards for data deposition, please see https://journals.plos.org/plosone/s/recommended-repositories.

Response: The dataset used in this study is publicly available from Kaggle. As per PLoS guidelines, we have provided direct access details and clarified that no restrictions apply.

7. If the reviewer comments include a recommendation to cite specific previously published works, please review and evaluate these publications to determine whether they are relevant and should be cited. There is no requirement to cite these works unless the editor has indicated otherwise.

Response: Thanks for this reminder. No, the reviewers have not suggested any references to cite.

Reviewers' comments:

Reviewer #1:

This paper develops and evaluates ensemble learning models, particularly stacking, for lung cancer risk prediction using lifestyle and clinical data. Multiple base and ensemble methods are compared across original, balanced, and upsampled datasets to assess predictive performance. SHAP and LIME are applied to interpret the models and understand the contribution of individual factors.

Major Concerns:-

1. The manuscript does not clearly explain whether SMOTE was applied strictly within each training fold during k-fold cross-validation. If synthetic samples were generated before splitting or shared across folds, this could introduce data leakage and artificially inflate performance.

Response: We appreciate the reviewer’s careful observation regarding the potential for data leakage when applying SMOTE in conjunction with cross-validation. This is indeed a critical methodological concern.

In our current implementation, SMOTE-based resampling was applied prior to cross-validation, resulting in three static datasets (original, balanced, and fully upsampled), on which stratified 10-fold cross-validation was subsequently performed. We clarify that SMOTE was applied to balanced and fully upsampled versions. While in balanced set, SMOTE was used to upsample only the minor class, in the fully upsampled set, SMOTE was used to upsample both classes. We agree that this differs from the strict “within-fold” resampling protocol typically recommended to completely eliminate any possibility of information leakage.

Our decision was driven by a practical constraint: the original dataset is extremely small and highly imbalanced, with only 39 samples in the minority class. Applying SMOTE strictly within each fold would lead to folds with very few minority samples, making both model training and validation unstable and highly sensitive to sampling variance. To address this, we constructed augmented datasets to enable more reliable learning and comparative evaluation across ensemble strategies.

That said, the reviewer’s concern is valid: performing resampling before cross-validation can, in principle, introduce optimistic bias due to shared synthetic structure across folds. We have taken several steps to mitigate and assess this risk:

• We evaluated models across three dataset regimes (original, SMOTE-balanced, and fully upsampled), rather than relying on a single augmented dataset.

• We conducted learning curve analysis, which showed that simpler models (e.g., voting) exhibited overfitting on the upsampled data, while the stacking model maintained stable training–validation alignment.

• Performance improvements were consistent across datasets, not confined to the augmented versions.

However, we acknowledge that these checks do not fully substitute for a leakage-free evaluation protocol. We have also added a discussion of the limitations of pre-CV resampling, including its potential impact on performance estimates.

2. Some models achieve near-perfect performance on the upsampled dataset, which raises concerns about overfitting.

Response: We thank the reviewer for this important observation. We agree that the near-perfect performance on the upsampled dataset warrants careful scrutiny for potential overfitting.

To investigate this, we analyzed the learning curves of both voting and stacking models (Fig. 14). On the original and SMOTE-balanced datasets, both models exhibited closely aligned training and validation curves, indicating stable learning and good generalization.

On the fully upsampled dataset, however, a clear difference emerged: the voting model showed noticeable divergence between training and validation performance, suggesting overfitting to the synthetic data. In contrast, the stacking model maintained close alignment between the curves, indicating more stable generalization even under aggressive upsampling.

We also relied on stratified cross-validation to reduce variance in performance estimates. Importantly, the strong performance of the stacking model was not limited to the upsampled dataset—it remained consistently high across the original and balanced datasets as well.

That said, we acknowledge that performance on heavily upsampled data can be optimistic. We have added an explicit discussion on this in Section 8.

3. Adding to the previous point, the models are evaluated on variations of a single dataset without testing on an independent external cohort, limiting generalizability.

Response: We thank the reviewer for highlighting this important limitation. We agree that evaluation on an independent external cohort would provide a stronger assessment of generalizability. However, we could not obtain an external dataset with similar parameters for validation. Parameter matching is crucial in our study, as we identified the contribution of specific predicate variables to lung cancer.

As an alternative approach, this study evaluated all models on variations of a single dataset (original, SMOTE-balanced, and fully upsampled). These variants were designed to test model behavior under different sample distributions and data volumes, rather than to serve as true external validation. While the consistent performance of the stacking model across these settings suggests robustness to data imbalance and sampling variations, it does not fully establish generalizability to unseen populations.

We acknowledge that the absence of an external or multi-institutional dataset limits the clinical applicability of the current findings. We will revise the manuscript to explicitly state this limitation.

As part of future work, we plan to explore extensively for similar datasets and validate the proposed model on independent datasets from different sources to assess its real-world generalization capability.

4. The strategy of upsampling both minority and majority classes to substantially enlarge the dataset is unconventional and may distort real-world class distributions, affecting generalizability

Response: We thank the reviewer for this insightful observation. We agree that upsampling both minority and majority classes is unconventional and does not reflect real-world class distributions.

Our intention with the fully upsampled dataset was not to model realistic prevalence, but to create a controlled setting to examine how different ensemble methods behave when sufficient data volume is available. Given the very small size of the original dataset, this approach allowed us to study model capacity, stability, and comparative performance at higher sample densities.

This approach resulted in overall improved efficacy for the ensemble models in terms of not only standard evaluation metrics but also statistical significance, and XAI-based interpretability.

However, we emphasize that this upsampled dataset was used strictly for methodological exploration, alongside the original and SMOTE-balanced datasets, rather than as a proxy for real-world deployment. Importantly, the models were also evaluated on the original dataset, which preserves the natural class distribution.

Nevertheless, we are not denying that training on artificially expanded data may distort the underlying data distributions and lead to overly optimistic performance estimates. We have added this aspect in the limitation section of the revised manuscript.

5. Although performance differences between models are reported, no statistical tests (e.g. paired tests across folds) are provided to show whether differences are statistically significant.

Response: We thank the reviewer for this important suggestion. We agree that statistical evaluation is necessary to support the significance of performance differences between models.

To address this, we conducted a non-parametric statistical analysis. Specifically, we performed a multiple-group (one-vs-all) comparison, where the stacking and voting models (with selected base learners) were evaluated against all base models. In addition, pairwise comparisons were carried out between the stacking and voting models.

These tests were performed across cross-validation folds, and the results confirm that the performance improvements achieved by the stacking model are statistically significant rather than due to random variation. The statistical significance is included in Section 6.

6. SHAP results indicate relatively low importance for smoking compared to symptoms, which contradicts epidemiological evidence and may reflect dataset bias that requires further clarification.

Response: We thank the reviewer for this important and valid observation. We agree that the relatively low importance of smoking in the SHAP analysis appears counterintuitive given its well-established role as the primary risk factor for lung cancer.

We would like to clarify that this behaviour has been explicitly analysed and discussed in the revised manuscript. Our findings suggest that this apparent discrepancy is primarily driven by the nature of the dataset and feature interactions rather than a contradiction of established epidemiological evidence. Specifically, the dataset is more symptom-oriented and reflects patient-level clinical manifestations rather than long-term exposure history. As a result, the model tends to assign higher importance to features that directly capture the physiological consequences of smoking (e.g., yellow fingers, shortness of breath) rather than the smoking variable itself.

This effect is further reinforced by multicollinearity, where the predictive signal of smoking is distributed across highly correlated downstream features. Consequently, SHAP—being a model-specific attribution method—allocates importance to the features that the model directly relies on for prediction, rather than to causal risk factors. This explains why smoking appears less prominent despite its underlying causal role.

Importantly, our interpretability analysis does not treat these findings in isolation. We provide a detailed clinical discussion, supported by relevant literature, showing that several top-ranked features (e.g., fatigue, alcohol consumption, swallowing difficulty) have plausible biological or clinical associations with lung cancer, while also critically examining deviations such as the reduced importance of smoking and age. These deviations are explicitly acknowledged as potential indicators of dataset bias and feature representation limitations.

We have further clarified in the manuscript that model-derived feature importance should not be interpreted as a direct reflection of epidemiological causality, but rather as a representation of patterns learned from the available data. This distinction is essential for responsible clinical interpretation.

7. The paper has a clear objective and is technically solid, but it feels slightly overextended. It covers a lot of concepts. Tightening the focus around the main goal would improve overall impact.

Response: W

Attachment Submitted filename: Response to reviewers comments.docx

# Peer review: Decision Letter 1

**DOI:** 10.1371/journal.pone.0357291.r003

**Authors:** Amgad Muneer

1 Jun 2026

Dear Dr. Dutta Pramanik,

Thank you for submitting your manuscript to PLOS ONE. After careful consideration, we feel that it has merit but does not fully meet PLOS ONE’s publication criteria as it currently stands. Therefore, we invite you to submit a revised version of the manuscript that addresses the points raised during the review process.

Please submit your revised manuscript by **Jul 16 2026 11:59PM** . If you will need more time than this to complete your revisions, please reply to this message or contact the journal office at plosone@plos.org. When you're ready to submit your revision, log on to https://www.editorialmanager.com/pone/ and select the 'Submissions Needing Revision' folder to locate your manuscript file.

- A letter that responds to each point raised by the academic editor and reviewer(s). You should upload this letter as a separate file labeled 'Response to Reviewers'.
- A marked-up copy of your manuscript that highlights changes made to the original version. You should upload this as a separate file labeled 'Revised Manuscript with Track Changes'.
- An unmarked version of your revised paper without tracked changes. You should upload this as a separate file labeled 'Manuscript'.

If you would like to make changes to your financial disclosure, please include your updated statement in your cover letter. Guidelines for resubmitting your figure files are available below the reviewer comments at the end of this letter.

If applicable, we recommend that you deposit your laboratory protocols in protocols.io to enhance the reproducibility of your results. Protocols.io assigns your protocol its own identifier (DOI) so that it can be cited independently in the future. For instructions see: https://journals.plos.org/plosone/s/submission-guidelines#loc-laboratory-protocols. Additionally, PLOS ONE offers an option for publishing peer-reviewed Lab Protocol articles, which describe protocols hosted on protocols.io. Read more information on sharing protocols at https://plos.org/protocols?utm_medium=editorial-email&utm_source=authorletters&utm_campaign=protocols.

As the corresponding author, your ORCID iD is verified in the submission system and will appear in the published article. PLOS supports the use of ORCID, and we encourage all coauthors to register for an ORCID iD and use it as well. Please encourage your coauthors to verify their ORCID iD within the submission system before final acceptance, as unverified ORCID iDs will not appear in the published article. *Only*  the individual author can complete the verification step; PLOS staff *cannot*  verify ORCID iDs on behalf of authors.

We look forward to receiving your revised manuscript.

Kind regards,

Amgad Muneer

Academic Editor

PLOS One

Journal Requirements:

If the reviewer comments include a recommendation to cite specific previously published works, please review and evaluate these publications to determine whether they are relevant and should be cited. There is no requirement to cite these works unless the editor has indicated otherwise.

Reviewers' comments:

Reviewer's Responses to Questions

**Comments to the Author**

Reviewer #2: (No Response)

**********

2. Is the manuscript technically sound, and do the data support the conclusions?

Reviewer #2: Yes

**********

3. Has the statistical analysis been performed appropriately and rigorously?

Reviewer #2: Yes

**********

4. Have the authors made all data underlying the findings in their manuscript fully available?

The PLOS Data policy

Reviewer #2: Yes

**********

5. Is the manuscript presented in an intelligible fashion and written in standard English?

Reviewer #2: Yes

**********

Reviewer #2: I have following suggestions:

Add leakage‑free validation as a robustness check. Even with current limitations, perform one additional experiment: apply SMOTE strictly inside each training fold of a single train/test split. Report whether stacking’s performance drops significantly. If it does, acknowledge this openly; if not, it strengthens your claims.

Remove or heavily downplay the upsampled‑dataset results. The 99.94% accuracy is unrealistic and harms credibility. Keep it only in a supplementary section or as a footnote. Emphasize original‑dataset performance (≈93%) as the primary finding in abstract, results, and conclusion.

Run a controlled experiment to explain the smoking paradox. Use SHAP on a simplified model (e.g., only smoking + yellow fingers) to show how multicollinearity redistributes importance. Present this as a small additional figure or table to convincingly demonstrate the effect.

Add a quantitative diversity metric for the stacking base learners. Compute pairwise Q‑statistics or correlation of errors among LR, CB, XGB, RF, ET. Show that diversity (not just individual accuracy) contributes to stacking’s gain. This directly addresses Reviewer #2’s concern about model selection.

Provide a concrete clinical workflow sketch. Add a short paragraph or a simple diagram showing: patient intake → model outputs (risk score + top 3 SHAP/LIME factors) → clinician review → recommended action (e.g., low‑dose CT referral). This makes XAI integration actionable.

Move runtime analysis to supplementary material. Since reviewers called it irrelevant for non‑time‑sensitive prediction, relocate Fig. 27 and related text to an appendix. Replace it with a brief statement on computational complexity (e.g., training vs. inference time) to keep focus on clinical utility.

**********

what does this mean?). If published, this will include your full peer review and any attached files.

If you choose “no”, your identity will remain anonymous but your review may still be made public.

**Do you want your identity to be public for this peer review?**  For information about this choice, including consent withdrawal, please see our Privacy Policy

Reviewer #2: No

**********

[NOTE: If reviewer comments were submitted as an attachment file, they will be attached to this email and accessible via the submission site. Please log into your account, locate the manuscript record, and check for the action link "View Attachments". If this link does not appear, there are no attachment files.]

To ensure your figures meet our technical requirements, please review our figure guidelines: https://journals.plos.org/plosone/s/figures

You may also use PLOS’s free figure tool, NAAS, to help you prepare publication quality figures: https://journals.plos.org/plosone/s/figures#loc-tools-for-figure-preparation.

NAAS will assess whether your figures meet our technical requirements by comparing each figure against our figure specifications.

# Author comment: Author response to Decision Letter 2

**DOI:** 10.1371/journal.pone.0357291.r004

26 Jun 2026

Manuscript ID: PONE-D-25-51253R1

Title: Lung Cancer Risk Prediction Using Interpretable Ensemble Models on Lifestyle and Clinical Data

We thank the reviewers and editor for valuable comments. We have carefully addressed these comments and revised the manuscript accordingly. For ease of evaluation, all modifications made in the revised manuscript have been highlighted in yellow. We thank the editor and reviewers for their constructive feedback, which has helped us improve the quality, clarity, and rigor of the manuscript. A detailed point-by-point response to all comments is provided below.

1. Add leakage free validation as a robustness check. Even with current limitations, perform one additional experiment: apply SMOTE strictly inside each training fold of a single train/test split. Report whether stacking’s performance drops significantly. If it does, acknowledge this openly; if not, it strengthens your claims.

Response: We thank the reviewer for this constructive suggestion. We agree that evaluating the models under a leakage-free resampling protocol provides a more rigorous assessment of their robustness.

Following the reviewer's recommendation, we conducted an additional experiment in which SMOTE was applied only after data splitting, with synthetic samples generated exclusively from the training data. The performance of the voting and stacking ensembles obtained under this protocol was then compared with the results from our original experimental setting. The results are presented in the newly added Table 9 and discussed in Section 5.4.2.

Interestingly, the performance of the proposed stacking model did not deteriorate under the stricter evaluation protocol. For the balanced dataset, the selected-5 stacking model improved from 93.78% to 95.67% accuracy, while its F1-score increased from 93.98% to 95.87%. A similar trend was observed for the voting models. For the upsampled dataset, the stacking ensemble continued to achieve the strongest overall performance, attaining 99.94% accuracy, 99.47% recall, and 99.74% AUC when SMOTE was applied after splitting.

An important observation is that the relative ranking of the models remained unchanged. Across both evaluation settings, stacking consistently outperformed voting and maintained its advantage over the constituent base learners. These results indicate that the performance gains reported in the manuscript are not dependent on a particular SMOTE application strategy and persist under a leakage-free resampling protocol.

We appreciate the reviewer for raising this point, as the additional experiment has strengthened the validation of the proposed framework and has been incorporated into the revised manuscript.

2. Remove or heavily downplay the upsampled dataset results. The 99.94% accuracy is unrealistic and harms credibility. Keep it only in a supplementary section or as a footnote. Emphasize original dataset performance (≈93%) as the primary finding in abstract, results, and conclusion.

Response: We thank the reviewer for this important observation. We agree that the near-perfect performance obtained on the upsampled dataset should not be interpreted as representative of real-world clinical deployment.

The purpose of including the balanced and upsampled datasets was to systematically examine the effect of class distribution and dataset size on model performance, rather than to establish deployment-level performance claims. For this reason, we prefer to retaine the results for all three dataset variants to provide a complete comparative evaluation framework.

At the same time, we have taken several steps to ensure that the findings are not overly centered on the upsampled results. In the revised manuscript, the discussion, limitations, and conclusions explicitly acknowledge that synthetic augmentation may lead to optimistic performance estimates and that the results obtained on the original dataset provide a more realistic assessment of practical applicability. We also expanded the state-of-the-art comparison (Table 10) to include results from all three dataset variants, whereas the previous version focused primarily on the upsampled dataset.

Accordingly, the manuscript distinguishes between experimental findings obtained under augmented-data conditions and those obtained on the original dataset, enabling readers to interpret the reported performance in the appropriate context. This update is reflected in the Abstract, Discussion and Conclusion sections.

3. Run a controlled experiment to explain the smoking paradox. Use SHAP on a simplified model (e.g., only smoking + yellow fingers) to show how multicollinearity redistributes importance. Present this as a small additional figure or table to convincingly demonstrate the effect.

Response: We thank the reviewer for this insightful observation and suggestion. We agree that the relatively low SHAP importance assigned to smoking, despite its established role as the dominant risk factor for lung cancer, warrants further investigation.

Rather than constructing a simplified two-feature model, we performed a broader analysis to examine whether this behavior persists across different ensemble architectures and dataset configurations. Specifically, we analyzed feature importance for both the voting and stacking models on the original, balanced, and upsampled datasets (Section 5.3.5). Interestingly, in all cases, smoking exhibited relatively low importance, while smoking-associated manifestations such as yellow fingers, chronic cough, and shortness of breath consistently received higher importance scores. This indicates that the observation is not specific to a particular model or dataset variant.

To further investigate this phenomenon, we conducted a multicollinearity analysis (Section 8) using the VIF. The results revealed substantial correlations among smoking-related predictors, with smoking (VIF = 11.16), yellow fingers (VIF = 19.08), chronic cough (VIF = 17.47), shortness of breath (VIF = 18.78), and alcohol consumption (VIF = 17.79) all exhibiting high VIF values. These findings suggest that the predictive information associated with smoking is distributed across multiple correlated variables.

Consequently, attribution methods such as SHAP tend to distribute explanatory importance among correlated predictors rather than assigning the entire contribution to a single feature. Therefore, the relatively lower importance assigned to smoking should not be interpreted as evidence of reduced clinical significance, but rather as a consequence of multicollinearity and the representation of smoking-related effects through correlated clinical manifestations. We have incorporated this analysis and discussion into the manuscript and added the corresponding VIF results to support this interpretation.

4. “Add a quantitative diversity metric for the stacking base learners. Compute pairwise Q statistics or correlation of errors among LR, CB, XGB, RF, ET. Show that diversity (not just individual accuracy) contributes to stacking’s gain. This directly addresses Reviewer #2’s concern about model selection.”

Response: We thank the reviewer for this valuable suggestion. We agree that the effectiveness of a stacking ensemble depends not only on the individual predictive strength of its constituent models but also on the diversity of their prediction behaviours.

To address this point, we performed an additional diversity analysis on the five selected base learners (LR, CB, XGB, RF, and ET) using pairwise Q-statistics and error-correlation measures. While our original statistical analysis demonstrated that the proposed stacking ensemble significantly outperformed many baseline models, it did not explicitly quantify the complementarity among the selected learners.

The diversity analysis revealed that the selected models exhibit a balanced degree of diversity despite their strong individual performance. For example, RF and ET showed the highest agreement (Q = 0.91, error correlation = 0.84), which is expected given their shared bagging-based architecture. In contrast, combinations involving LR and tree-based ensembles (e.g., LR–ET and LR–RF) exhibited substantially lower agreement, indicating greater diversity in decision boundaries and error patterns. Most remaining pairs demonstrated moderate diversity, suggesting that the selected learners provide complementary predictive information rather than redundant predictions.

These findings support our model-selection strategy and indicate that the performance gains of the stacking framework arise not only from the accuracy of the constituent learners but also from their diversity. We have incorporated this analysis into the manuscript and added a dedicated table reporting the pairwise diversity metrics.

5. Provide a concrete clinical workflow sketch. Add a short paragraph or a simple diagram showing: patient intake → model outputs (risk score + top 3 SHAP/LIME factors) → clinician review → recommended action (e.g., low dose CT referral). This makes XAI integration actionable.

Response: We thank the reviewer for this valuable suggestion. We agree that the practical use of explainable AI in a clinical setting should be more explicitly illustrated. To address this point, we have added a conceptual clinical workflow figure (Figure 35) and accompanying discussion describing how the proposed framework could be integrated into routine clinical practice. The workflow demonstrates how patient demographic, lifestyle, and symptom-related information is processed by the prediction model to generate both a risk score and SHAP/LIME-based explanations. These outputs are then reviewed by clinicians and can support decisions regarding additional assessment, low-dose CT screening, specialist referral, or routine follow-up.

6. Move runtime analysis to supplementary material. Since reviewers called it irrelevant for non time sensitive prediction, relocate Fig. 27 and related text to an appendix. Replace it with a brief statement on computational complexity (e.g., training vs. inference time) to keep focus on clinical utility.

Response: We thank the reviewer for this valuable suggestion. We agree that detailed runtime comparisons are not central to the primary objective of this study, which is to evaluate the predictive performance and interpretability of ensemble models for lung cancer risk prediction.

Accordingly, we have removed the runtime comparison figure and the associated detailed analysis from the manuscript. Instead, we briefly discuss the computational implications of the proposed models in the Discussion section. Specifically, we acknowledge the trade-off between predictive performance and computational cost, noting that the stacking ensemble achieves superior accuracy at the expense of increased computational complexity. However, since lung cancer risk prediction is not a real-time application, the additional computational overhead is unlikely to be a major limitation in typical clinical environments. We also note that computational efficiency may become relevant in large-scale screening programs or resource-constrained deployment settings.

Attachment Submitted filename: Response to reviewers comments (2nd revision).docx

# Editor report: Decision Letter 2

**DOI:** 10.1371/journal.pone.0357291.r005

**Authors:** Amgad Muneer

10 Jul 2026

Dear Dr. Dutta Pramanik,

Thank you for submitting your manuscript to PLOS ONE. After careful consideration, we feel that it has merit but does not fully meet PLOS ONE’s publication criteria as it currently stands. Therefore, we invite you to submit a revised version of the manuscript that addresses the following points raised during the review process.

Please submit your revised manuscript by Aug 24 2026 11:59PM. If you will need more time than this to complete your revisions, please reply to this message or contact the journal office at plosone@plos.org. When you're ready to submit your revision, log on to https://www.editorialmanager.com/pone/ and select the 'Submissions Needing Revision' folder to locate your manuscript file.

- A letter that responds to each point raised by the academic editor and reviewer(s). You should upload this letter as a separate file labeled 'Response to Reviewers'.
- A marked-up copy of your manuscript that highlights changes made to the original version. You should upload this as a separate file labeled 'Revised Manuscript with Track Changes'.
- An unmarked version of your revised paper without tracked changes. You should upload this as a separate file labeled 'Manuscript'.

If you would like to make changes to your financial disclosure, please include your updated statement in your cover letter. Guidelines for resubmitting your figure files are available below the reviewer comments at the end of this letter.

If applicable, we recommend that you deposit your laboratory protocols in protocols.io to enhance the reproducibility of your results. Protocols.io assigns your protocol its own identifier (DOI) so that it can be cited independently in the future. For instructions see: https://journals.plos.org/plosone/s/submission-guidelines#loc-laboratory-protocols. Additionally, PLOS ONE offers an option for publishing peer-reviewed Lab Protocol articles, which describe protocols hosted on protocols.io. Read more information on sharing protocols at https://plos.org/protocols?utm_medium=editorial-email&utm_source=authorletters&utm_campaign=protocols.

As the corresponding author, your ORCID iD is verified in the submission system and will appear in the published article. PLOS supports the use of ORCID, and we encourage all coauthors to register for an ORCID iD and use it as well. Please encourage your coauthors to verify their ORCID iD within the submission system before final acceptance, as unverified ORCID iDs will not appear in the published article. *Only*  the individual author can complete the verification step; PLOS staff *cannot*  verify ORCID iDs on behalf of authors.

We look forward to receiving your revised manuscript.

Kind regards,

Amgad Muneer

Academic Editor

PLOS One

**Journal Requirements:**

1. If the reviewer comments include a recommendation to cite specific previously published works, please review and evaluate these publications to determine whether they are relevant and should be cited. There is no requirement to cite these works unless the editor has indicated otherwise.

2. Please review your reference list to ensure that it is complete and correct. If you have cited papers that have been retracted, please include the rationale for doing so in the manuscript text, or remove these references and replace them with relevant current references. Any changes to the reference list should be mentioned in the rebuttal letter that accompanies your revised manuscript. If you need to cite a retracted article, indicate the article’s retracted status in the References list and also include a citation and full reference for the retraction notice.

**Additional Editor Comments:**

The manuscript has improved substantially and most of the technical concerns have been addressed. However, before acceptance, the authors should complete the following revisions to ensure that the claims are appropriately calibrated and the study is fully reproducible:

1. The 99.94% accuracy should be removed from the Abstract or clearly labeled as a synthetic-data sensitivity analysis, not as a clinically realistic performance estimate. The original-dataset performance of 93.53% should be emphasized as the primary result throughout the Abstract, Results, Discussion, and Conclusion.
2. The response states that SMOTE was applied after data splitting and only to the training data, but the manuscript should explicitly confirm that SMOTE and all preprocessing steps were applied strictly within the training fold only, with validation/test data left completely untouched during model fitting, tuning, and resampling. This is important because the original reviewer request specifically asked for SMOTE inside the training fold.
3. The added workflow is useful, but the manuscript should clearly state that the model is not ready for clinical deployment or low-dose CT referral decisions without prospective and external validation. The workflow should be presented as a conceptual decision-support framework only, not a clinical recommendation. Please temper all clinical-implementation language.
4. The authors should provide sufficient implementation details, including hyperparameters, random seeds, train/test split strategy, SMOTE settings. This will help ensure that the reported results can be independently reproduced.
5. The authors should explicitly state that the study relies on a single public Kaggle dataset, lacks external validation, may contain dataset-specific patterns, and that results from balanced/upsampled datasets may overestimate real-world performance.
6. Please conduct final language polishing.

[NOTE: If reviewer comments were submitted as an attachment file, they will be attached to this email and accessible via the submission site. Please log into your account, locate the manuscript record, and check for the action link "View Attachments". If this link does not appear, there are no attachment files.]

To ensure your figures meet our technical requirements, please review our figure guidelines: https://journals.plos.org/plosone/s/figures

You may also use PLOS’s free figure tool, NAAS, to help you prepare publication quality figures: https://journals.plos.org/plosone/s/figures#loc-tools-for-figure-preparation.

NAAS will assess whether your figures meet our technical requirements by comparing each figure against our figure specifications.

# Author comment: Author response to Decision Letter 3

**DOI:** 10.1371/journal.pone.0357291.r006

13 Jul 2026

1. The 99.94% accuracy should be removed from the Abstract or clearly labeled as a synthetic-data sensitivity analysis, not as a clinically realistic performance estimate. The original-dataset performance of 93.53% should be emphasized as the primary result throughout the Abstract, Results, Discussion, and Conclusion.

Response: We appreciate the Editor's suggestion and have revised the manuscript accordingly. The explicit mention of the 99.94% accuracy has been removed from both the Abstract and the Conclusion to avoid presenting the performance on the synthetically upsampled dataset as a clinically realistic outcome. Throughout the manuscript, we now place greater emphasis on the model's performance on the original dataset (93.53% accuracy) as the most representative estimate of its practical applicability. The results obtained on the balanced and upsampled datasets are retained only in the Results (Section 5) for comparative analysis to illustrate the effect of different data augmentation strategies, and are discussed with appropriate caution as sensitivity analyses rather than indicators of expected real-world clinical performance.

2. The response states that SMOTE was applied after data splitting and only to the training data, but the manuscript should explicitly confirm that SMOTE and all preprocessing steps were applied strictly within the training fold only, with validation/test data left completely untouched during model fitting, tuning, and resampling. This is important because the original reviewer request specifically asked for SMOTE inside the training fold.

Response: Thank you for this valuable comment. We would like to clarify that the leakage-free SMOTE protocol was implemented as an additional robustness analysis in response to Reviewer 1's recommendation. Specifically, an additional experiment was conducted in which SMOTE was applied only after data splitting and exclusively to the training partition within each fold, while the corresponding validation fold remained completely untouched throughout resampling, model training, hyperparameter tuning, and performance evaluation. This protocol and the corresponding results are described in Section 5.4.2 and summarized in Table 9. The purpose of this additional experiment was to verify whether the performance of the proposed ensemble models remained stable under a stricter evaluation protocol. The results show that the stacking model did not exhibit any performance degradation. For the balanced dataset, the selected-5 stacking model improved from 93.78% to 95.67% accuracy, while its F1-score increased from 93.98% to 95.87%. Likewise, for the upsampled dataset, the stacking model continued to achieve the strongest overall performance, attaining 99.94% accuracy, 99.47% recall, and 99.74% AUC. These results indicate that the conclusions of the study remain unchanged under the leakage-free validation protocol.

3. The added workflow is useful, but the manuscript should clearly state that the model is not ready for clinical deployment or low-dose CT referral decisions without prospective and external validation. The workflow should be presented as a conceptual decision-support framework only, not a clinical recommendation. Please temper all clinical-implementation language.

Response: Thank you for this valuable observation. We agree that the proposed framework should not be presented as being ready for routine clinical use. Accordingly, we have revised the manuscript to consistently describe the proposed workflow as a conceptual decision-support framework rather than a clinical implementation pathway. The discussion has been updated to clarify that the workflow illustrates a potential future integration of explainable AI into clinical practice and should not be interpreted as a recommendation for current clinical deployment or referral decisions. To further reinforce this distinction, the title of Fig. 35 have also been revised to explicitly identify it as a conceptual decision-support workflow. In addition, the limitations section already states that prospective studies and independent external validation are required before the proposed framework can be considered for routine clinical use.

4. The authors should provide sufficient implementation details, including hyperparameters, random seeds, train/test split strategy, SMOTE settings. This will help ensure that the reported results can be independently reproduced.

Response: Thank you for this helpful suggestion. To improve the reproducibility of the study, we have expanded the implementation details in the revised manuscript. The manuscript now explicitly describes the 75%/25% train-test split strategy, the use of stratified 10-fold cross-validation for model optimization, the SMOTE configuration (sampling_strategy='auto', k_neighbors=5), and the leakage-free robustness experiment in which SMOTE was applied exclusively to the training data after data splitting. The optimized hyperparameter values of all models are reported in Table 4. In addition, to facilitate independent verification and reproduction of the results, we have made the complete implementation publicly available through a GitHub repository, the link to which is provided in the Declaration section of the manuscript.

5. The authors should explicitly state that the study relies on a single public Kaggle dataset, lacks external validation, may contain dataset-specific patterns, and that results from balanced/upsampled datasets may overestimate real-world performance.

Response: Thank you for this helpful suggestion. We have revised the limitations section to make these points more explicit. The manuscript now clearly states that the study is based on a single publicly available Kaggle dataset, which may contain dataset-specific characteristics that limit generalizability. We also reiterate that the proposed models have not undergone external validation and that the current findings should therefore be regarded as preliminary. In addition, we have strengthened the discussion on synthetic data augmentation by explicitly stating that the results obtained on the balanced and particularly the upsampled datasets should be interpreted as comparative analyses under augmented data conditions and not as estimates of expected real-world clinical performance.

6. Please conduct final language polishing.

Response: Thank you for the suggestion. The manuscript has been carefully proofread, and minor revisions have been made to improve grammar, clarity, and readability while preserving the technical content and intended meaning.

Attachment Submitted filename: Response to editors comments.docx

# Editor report: Decision Letter 3

**DOI:** 10.1371/journal.pone.0357291.r007

**Authors:** Amgad Muneer

16 Aug 2026

Lung cancer risk prediction using interpretable ensemble models on lifestyle and clinical data

PONE-D-25-51253R3

Dear Dr. Pramanik,

We’re pleased to inform you that your manuscript has been judged scientifically suitable for publication and will be formally accepted for publication once it meets all outstanding technical requirements.

Within one week, you’ll receive an e-mail detailing the required amendments. When these have been addressed, you’ll receive a formal acceptance letter and your manuscript will be scheduled for publication.

An invoice will be generated when your article is formally accepted. Please note, if your institution has a publishing partnership with PLOS and your article meets the relevant criteria, all or part of your publication costs will be covered. Please make sure your user information is up-to-date by logging into Editorial Manager at Editorial Manager® and clicking the ‘Update My Information' link at the top of the page. For questions related to billing, please contact billing support.

If your institution or institutions have a press office, please notify them about your upcoming paper to help maximize its impact. If they’ll be preparing press materials, please inform our press team as soon as possible -- no later than 48 hours after receiving the formal acceptance. Your manuscript will remain under strict press embargo until 2 pm Eastern Time on the date of publication. For more information, please contact onepress@plos.org.

Kind regards,

Amgad Muneer

Academic Editor

PLOS One

# Editor report: Acceptance letter

**DOI:** 10.1371/journal.pone.0357291.r008

**Authors:** Amgad Muneer

PONE-D-25-51253R3

PLOS One

Dear Dr. Dutta Pramanik,

I'm pleased to inform you that your manuscript has been deemed suitable for publication in PLOS One. Congratulations! Your manuscript is now being handed over to our production team.

At this stage, our production department will prepare your paper for publication. This includes ensuring the following:

* All references, tables, and figures are properly cited

* All relevant supporting information is included in the manuscript submission,

* There are no issues that prevent the paper from being properly typeset

You will receive further instructions from the production team, including instructions on how to review your proof when it is ready. Please keep in mind that we are working through a large volume of accepted articles, so please give us a few days to review your paper and let you know the next and final steps.

Lastly, if your institution or institutions have a press office, please let them know about your upcoming paper now to help maximize its impact. If they'll be preparing press materials, please inform our press team within the next 48 hours. Your manuscript will remain under strict press embargo until 2 pm Eastern Time on the date of publication. For more information, please contact onepress@plos.org.

You will receive an invoice from PLOS for your publication fee after your manuscript has reached the completed accept phase. If you receive an email requesting payment before acceptance or for any other service, this may be a phishing scheme. Learn how to identify phishing emails and protect your accounts at https://explore.plos.org/phishing.

If we can help with anything else, please email us at customercare@plos.org.

Thank you for submitting your work to PLOS One and supporting open access.

Kind regards,

PLOS One Editorial Office Staff

on behalf of

Dr. Amgad Muneer

Academic Editor

PLOS One
